# Development of Sulfamoylated 4-(1-Phenyl-1*H*-1,2,3-triazol-4-yl)phenol Derivatives as Potent Steroid Sulfatase Inhibitors for Efficient Treatment of Breast Cancer

**DOI:** 10.1021/acs.jmedchem.1c02220

**Published:** 2022-03-02

**Authors:** Karol Biernacki, Olga Ciupak, Mateusz Daśko, Janusz Rachon, Witold Kozak, Janusz Rak, Konrad Kubiński, Maciej Masłyk, Aleksandra Martyna, Magdalena Śliwka-Kaszyńska, Joanna Wietrzyk, Marta Świtalska, Alessio Nocentini, Claudiu T. Supuran, Sebastian Demkowicz

**Affiliations:** † Department of Organic Chemistry, Faculty of Chemistry, Gdańsk University of Technology, Narutowicza 11/12, 80-233 Gdansk, Poland; ‡ Department of Inorganic Chemistry, Faculty of Chemistry, Gdańsk University of Technology, Narutowicza 11/12, 80-233 Gdansk, Poland; § Department of Physical Chemistry, Faculty of Chemistry, University of Gdańsk, Wita Stwosza 63, 80-308 Gdansk, Poland; ∥ Department of Molecular Biology, Faculty of Biotechnology and Environment Sciences, The John Paul II Catholic University of Lublin, Konstantynów 1i, 20-708 Lublin, Poland; ⊥ Department of Experimental Oncology, Hirszfeld Institute of Immunology and Experimental Therapy, Rudolfa Weigla 12, 53-114 Wrocław, Poland; # Department of NEUROFARBA, Pharmaceutical and Nutraceutical Section, University of Florence, Via U. Schiff 6, Sesto Fiorentino, 50019 Firenze, Italy

## Abstract

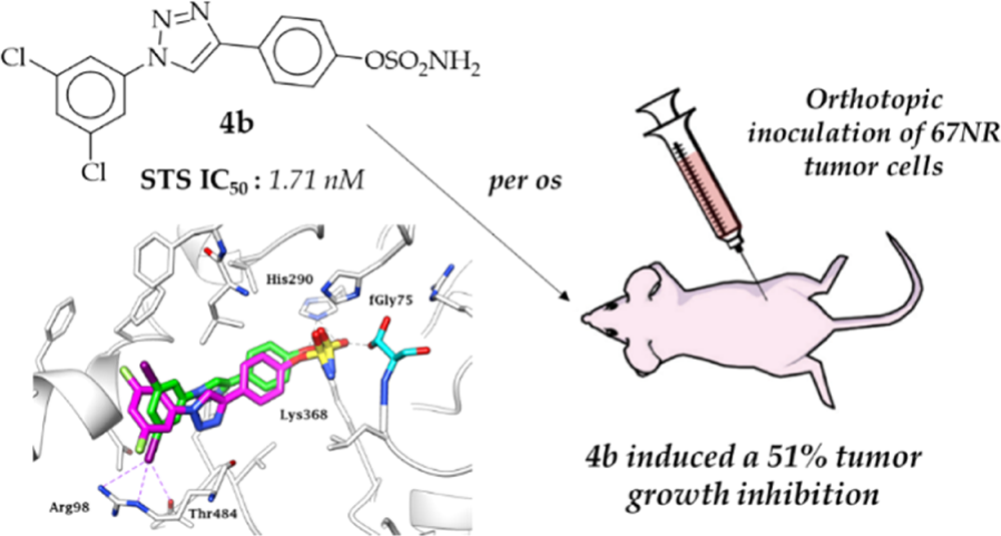

We present here the advances achieved in the development of new sulfamoylated 4-(1-phenyl-1*H*-1,2,3-triazol-4-yl)phenol derivatives as potent steroid sulfatase (STS) inhibitors for the treatment of breast cancer. Prompted by promising biological results and in silico analysis, the initial series of similar compounds were extended, appending a variety of m-substituents at the outer phenyl ring. The inhibition profiles of the newly synthesized compounds were evaluated using a radioisotope enzymatic assay and, together with the preceding reported derivatives, using a radioisotope assay in MCF-7 cells. The most active compound, **5l**, demonstrated an extraordinary STS inhibitory potency in MCF-7 cells with an IC_50_ value improved 5-fold compared to that of the reference **Irosustat** (0.21 vs 1.06 nM). The five most potent compounds were assessed in vivo in a 67NR mouse mammary gland cancer model, with **4b** measured to induce up to 51% tumor growth inhibition at 50 mg/kg with no evidence of side effects and toxicity.

## Introduction

A multitude of cancers show a hormone-dependent nature in their early stages, with a 95% correlation evidenced for breast cancer cases.^1^ Modern therapy tackles these tumors using pharmaceuticals that effectively reduce the availability of active hormones for cancer cells. However, current chemotherapeutic breast cancer therapies using inhibitors of the aromatase enzyme complex or selective estrogen receptor modulators (SERMs) often turn out to be unsatisfactory, resulting in high cancer relapse rates in patients.^2−4^ Notably, aromatase expression has been found only in 60% of breast cancer cases, while the expression of steroid sulfatase (STS) has been detected in 90% of breast tumors.^5^ STS is a crucial enzyme for steroidogenesis. It acts by hydrolyzing inactive steroid sulfates [including estrone-3-sulfate (E1S) and dehydroepiandrosterone-3-sulfate (DHEAS)],^6,7^ which are the precursors for the biosynthesis of active estrogens and androgens.^8^ Recent evidence prompted STS as an extremely important new molecular target in the development of novel and effective cancer therapies.^9^ STS inhibition may also be of relevance in the treatment of other hormone-dependent types of tumors, for example, endometrial and prostate cancers.^10^

**Figure 1:**
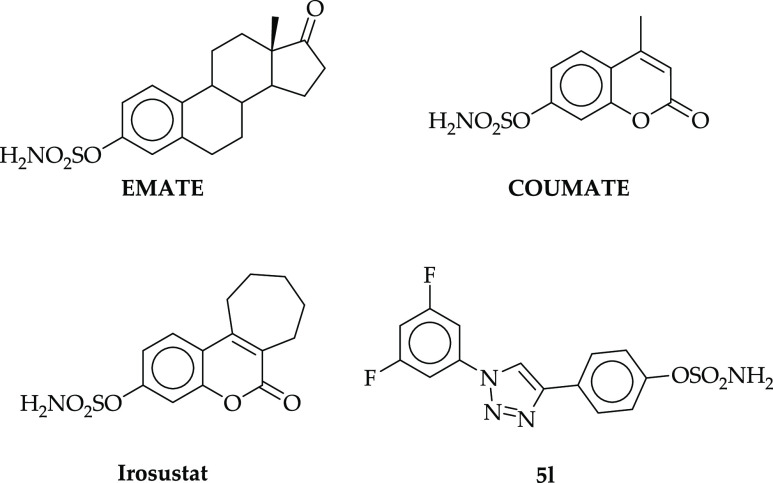
Chemical structures of STS inhibitors **EMATE**, **COUMATE**, **Irosustat**, and **5l**.

As a result, in the last few decades, scientists have been intensively dedicated to finding novel and effective STS inhibitors. The latter can be basically divided into steroidal and nonsteroidal derivatives.^9,11^ Among the steroidal STS inhibitors, **EMATE** (Figure 1) stood out as the most promising compound, exhibiting a great inhibitory effect with an IC_50_ value of 65 pM upon evaluation in MCF-7 cells.^12^ However, in some cases, the presence of the steroidal core resulted to be associated with the induction of side effects that limit clinical use, which include the estrogenic properties of metabolites leading to stimulation of tumor growth. Among nonsteroidal compounds, coumarin derivatives exhibited potent STS inhibition properties and reported fewer adverse effects and weaker estrogenic properties. Coumarin analogues, for example, **COUMATE** (Figure 1), are classified as irreversible, time-dependent, and concentration-dependent inhibitors. **COUMATE** exhibited a good activity with an IC_50_ value of 380 nM when evaluated in placental microsomes.^13^ Chemical modification of **COUMATE** led to the development of tricyclic coumarin derivatives series, such as **Irosustat** (Figure 1). The tricyclic core mimics the ABC rings occurring in natural substrates. **Irosustat** demonstrated a very potent STS inhibitory effect (IC_50_ value of 8 nM) with no in vivo and in vitro estrogenic properties. **Irosustat** resulted to be orally active and, as such, reached clinical trials,^14−16^ showing great therapeutic potential in several clinical studies.^17−21^ To date, other coumarin derivatives with sulfamate,^22^ phosphate,^23,24^ and thiophosphate^25−27^ moieties as well as fluorinated compounds^28,29^ have been reported as potent STS inhibitors.

Recently, the introduction of fluorine atoms into the structure of new STS inhibitors has been significantly pursued to increase the compound drug-like profiles, such as with piperazinyl-ureido sulfamates^30^ and *N*-acylated tyramine sulfamates.^31^ In 2020, we reported a new series of STS inhibitors based on the fluorinated 4-(1-phenyl-1*H*-1,2,3-triazol-4-yl)phenyl sulfamate core,^32^ considering the efficacy of 1,2,3-triazole derivatives for many biomedical applications such as antiviral, antibacterial, antitubercular, antimalarial, antileishmanial, or anticancer applications.^33,34^ Moreover, the structure of 1,4-diphenyl-substituted 1,2,3-triazole ring resembles the steroidal structure of natural STS substrates, which is one of the crucial aspects for designing potent STS inhibitors. We showed that derivatives bearing fluorine atoms at the meta position of the terminal aromatic ring exhibited the greatest inhibitory properties. The most active compound, namely, 4-(1-(3,5-difluorophenyl)-1*H*-1,2,3-triazol-4-yl)phenyl sulfamate, **5l** (Figure 1), inhibited STS with an IC_50_ value of 36.78 nM, as detected by the enzymatic assay. On the basis of these findings, we report here a new group of 4-(1-phenyl-1*H*-1,2,3-triazol-4-yl)phenyl sulfamates containing various substituents at the meta position of the terminal aromatic ring (including chlorine, bromine, and iodine atoms as well as methyl, ethyl, isopropyl, methoxy, and nitro groups). The newly synthesized compounds were evaluated for their STS inhibitory potency using a radioisotope enzymatic assay and, together with a previously described series of 4-(1-phenyl-1*H*-1,2,3-triazol-4-yl)phenyl sulfamates (ref (32)), using a radioisotope cellular assay in MCF-7 cells. The five most active compounds in vitro (**4a**, **4b**, **5e**, **5g**, and **5l**) were selected for in vivo antitumor studies in a 67NR mouse mammary gland carcinoma model.

## Results and Discussion

### Synthesis

**Scheme 1:**
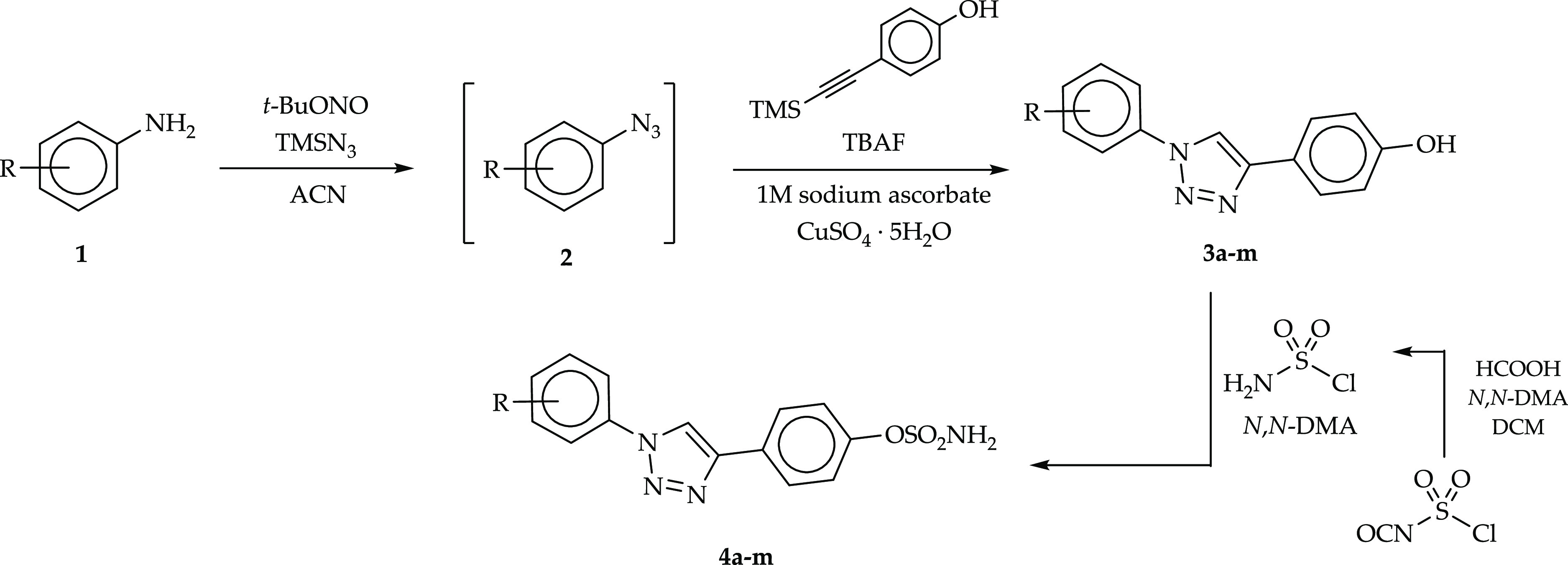
Synthetic Pathway for 4-(1-Phenyl-1*H*-1,2,3-triazol-4-yl)phenyl Sulfamate Derivatives **4a–m** [R = H, Cl, Br, I, CH_3_, OCH_3_, C_2_H_5_, CH(CH_3_)_2_, and NO_2_]

Promising results for molecular modeling studies and biological assays with the sulfamoylated 4-(1-phenyl-1*H*-1,2,3-triazol-4-yl)phenol analogues **5a–m** prompted us to expand the library of such active compounds with a variety of substituents appended at the meta position of the outer phenyl ring. The newly designed compounds **4a–m** were synthesized according to the synthetic protocol shown in Scheme 1. The first step of the synthetic pathway consisted in the conversion of appropriate aniline derivatives **1** into corresponding azides **2** with *tert*-butyl nitrite (*t*-BuONO) and azidotrimethylsilane (TMSN_3_) in acetonitrile (ACN). 4-(1-Phenyl-1*H*-1,2,3-triazol-4-yl)phenol derivatives **3a–m** were thus prepared using an azide–alkyne Huisgen cycloaddition reaction by adding in situ to the azide reaction mixture 4-[(trimethylsilyl)ethynyl]phenol and a 1 M solution of tetrabutylammonium fluoride (TBAF) in tetrahydrofuran and, successively, copper(II) sulfate pentahydrate (CuSO_4_·5H_2_O) and a 1 M aqueous solution of sodium ascorbate. The final products **4a–m** were obtained by treating 4-(1-phenyl-1*H*-1,2,3-triazol-4-yl)phenol derivatives **3a–m** with sulfamoyl chloride (generated in situ) under anhydrous conditions. Furthermore, the fluorinated 4-(1-phenyl-1*H*-1,2,3-triazol-4-yl)phenyl sulfamates **5a-m** were resynthesized in a larger scale according to a previously described procedure (ref (32)) to perform additional biological assays.

### In Vitro Enzymatic Assay

Initially, the inhibitory properties of the newly synthesized compounds **4a–m** were determined through the radioisotope enzymatic assay using STS isolated from the human placenta and radiolabeled substrate [^3^H]E1S. This screening research was carried out to assess the inhibitory potential of new STS inhibitor candidates and to select the most active compounds for further cellular investigations as well as for in vivo studies. The level of STS inhibition was compared with that of our previously synthesized derivatives **5g** and **5l**. The obtained results indicated that all newly synthesized compounds **4a–m** inhibited the STS enzyme in the submicromolar range (residual STS activity from 11.78 to 55.11% at a 0.5 μM inhibitor concentration) (Table 1). The most potent inhibitory effects were measured with both iodine-substituted compounds **4d** and **4m** and 3,5-diCl-substituted **4b**. The inhibitory properties of alkyl-substituted derivatives **4i** and **4j** were also relevant when compared to those of the previously described inhibitor **5l** (residual STS activity of 17.34%).

**Table 1:**
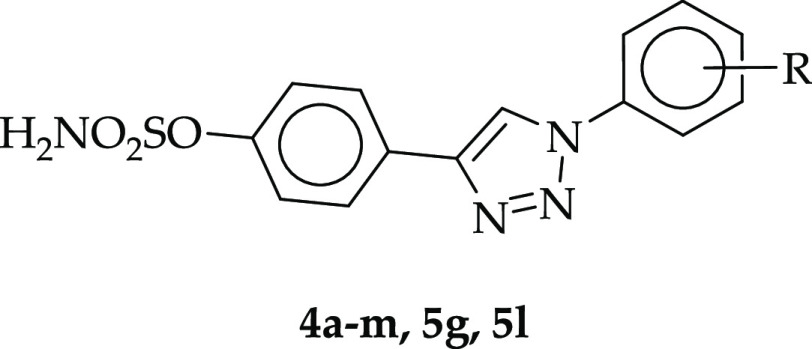
STS Inhibitory Effect of Compounds **4a–m** and Reference Inhibitors **5g** and **5l** Using the Radioisotope Enzymatic Assay at a 0.5 μM Inhibitor Concentration

no.	R	residual STS activity [%]^a^
**4a**	3-Cl	19.49 ± 0.97
**4b**	3,5-diCl	13.32 ± 0.67
**4c**	3-Br	24.05 ± 1.20
**4d**	3-I	13.23 ± 0.66
**4e**	3-CH_3_	34.23 ± 1.71
**4f**	3,5-diCH_3_	52.45 ± 2.62
**4g**	3-OCH_3_	43.35 ± 2.17
**4h**	3,5-diOCH_3_	55.11 ± 2.76
**4i**	3-CH_2_CH_3_	14.51 ± 0.73
**4j**	3-CH(CH_3_)_2_	18.10 ± 0.90
**4k**	3-NO_2_	28.88 ± 1.44
**4l**	3,5-diBr	28.88 ± 1.44
**4m**	3,5-diI	11.78 ± 0.59
**5g**	3-F	37.92 ± 1.90
**5l**	3,5-diF	17.34 ± 0.87

a Substrate: [3H]E1S, 3 nM; experiments were carried out in triplicate.

**Figure 2:**
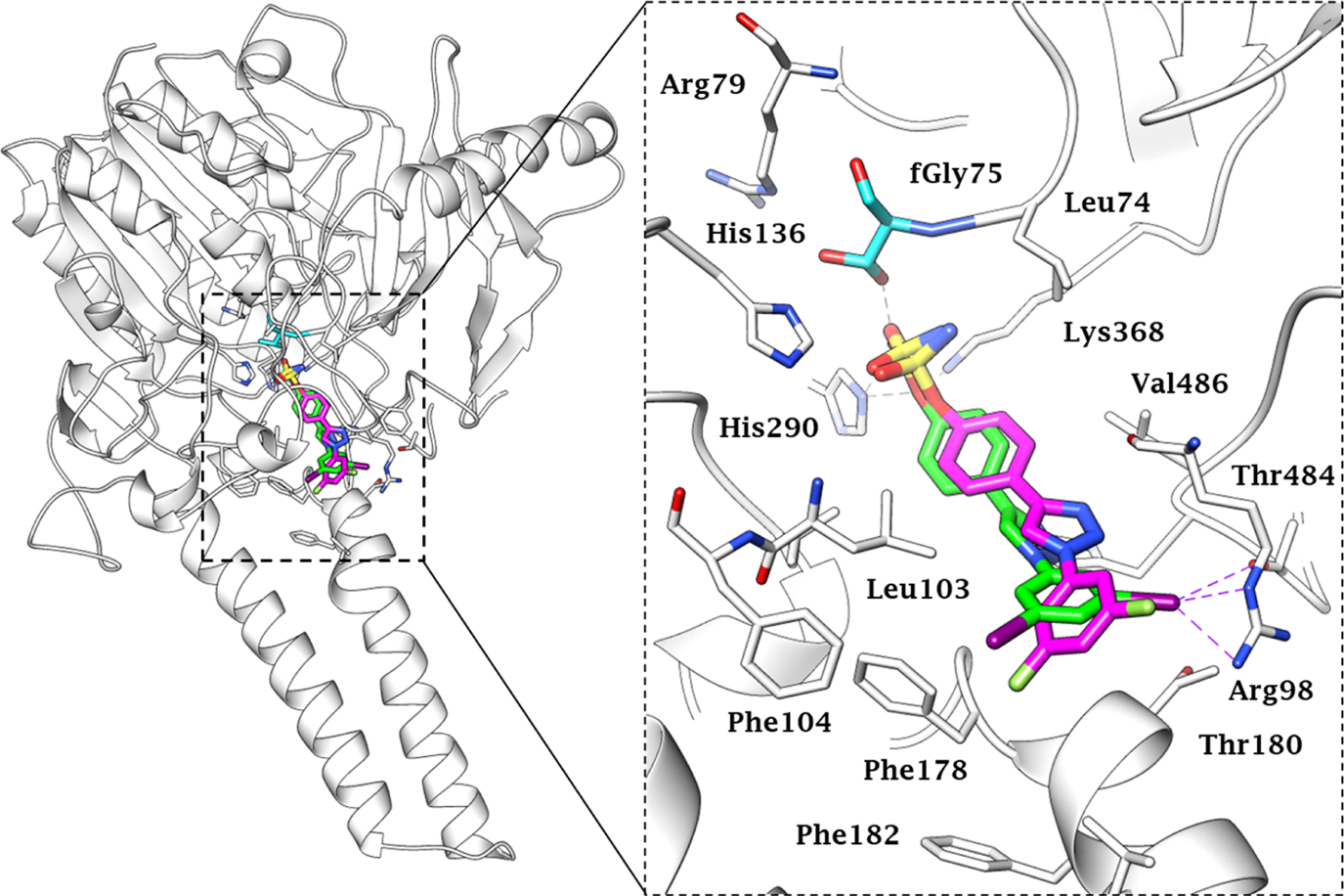
Predicted binding mode of compounds **4m** (green) and **5l** (magenta) in the STS active site (PDB 1P49), shown as an overall ribbon view (left) and an active site view (right). fGly residue is colored cyan. H-bonds and halogen bonds are represented as black and purple dashed lines, respectively.

Analysis of the structure–activity relationship (SAR) suggests that the capability of new compounds to inhibit STS depends on two main parameters, that is, hydrophobicity and the type of the halogen substituent. In fact, the introduction of an *m*-halogen substituent increases the hydrophobic nature of the outer core, making greater the contribution of hydrophobic interactions in the stabilization of the inhibitor–enzyme complex. Indeed, the obtained results showed that compounds bearing iodine atoms produced the greatest STS inhibition. As a matter of fact, molecular modeling studies showed that the iodine substituents are located close to residues Arg98 and Thr484 in the STS active site (Figure 2), giving rise to a halogen bond (X-bond) network in which the residues act as acceptors. Evidence exists that halogen bonds are actively implicated in the stabilization of inhibitor–enzyme complexes, though they are still the subject of scientific debates.

Overall, compound **4m** showed a very similar binding conformation to analogue **5l** in the STS active site. The sulfamate functional group, which is mainly responsible for the inactivation of the enzyme, binds in the enzyme catalytic region close to the formylglycine residue coordinated to the Ca^2+^ ion (not shown) by a H-bond network. Although the inhibition mechanism has not been validated so far, the sulfamate group (sulfate mimic) is speculated to undergo a nucleophilic substitution reaction with the fGly residue that results in the sulfamoylation and inactivation of the catalytic site.^17^ The triazole moieties as well as the triazole-linked aromatic rings of the ligands fit in the STS active site stabilized by a multitude of van der Waals interactions with Leu103, Leu167, Phe178, Phe182, Phe237, Val486, Phe488, and Phe553.

### Radioisotope Cellular Assay

As a second step, the inhibitory properties of compounds **4a–m** were assessed using a radioisotope assay with the radiolabeled substrate [^3^H]E1S in MCF-7 cells. The previously reported compounds **5a–m** were included in such a biological evaluation as well. **COUMATE** and **Irosustat** were used as reference inhibitors (Table 2). All compounds were initially tested for their in cell inhibitory action at a 100 nM concentration. Most inhibitors showed the capability to almost completely block the STS enzymatic activity. Only a 1% residual enzymatic activity was measured in the presence of a 100 nM concentration of **4–am**, **5i**, and **5l**, while activity levels below 5% were observed with compounds **5a–b**, **5h**, **5j**, **5m**, **4a–e**, and **4i–j**. While compound **4m** bearing two *meta*-iodine substituents showed the greatest inhibitory activity in the enzymatic assay using isolated STS, it turned out to be a weaker STS inhibitor (residual STS activity of 18.7% at 100 nM). It can be speculated that **4m** has a lower cell membrane permeability, which hinders its efficient inhibition. References **COUMATE** and **Irosustat** showed STS residual activities of 51.8 and 2.4%, respectively, at a 100 nM concentration.

**Table 2:**
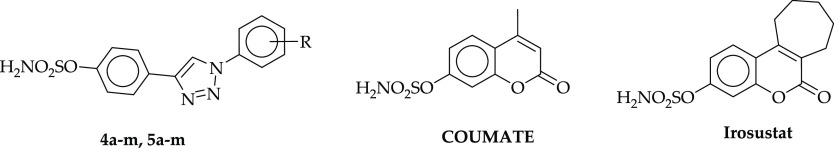
Residual STS Activity in MCF-7 Cells after Incubation with Compounds **4a–m**, **5-Am**, **COUMATE**, and **Irosustat** at 100, 10, and 1 nM Inhibitor Concentrations

		residual STS activity [%]^a^			
no.	R	100 nM	10 nM	1 nM	IC_50_ [nM]
**4a**	3-Cl	2.4 ± 0.07	28.1 ± 1.12	38.2 ± 1.34	1.90 ± 0.06
**4b**	3,5-diCl	2.0 ± 0.1	17.8 ± 0.62	63.7 ± 2.55	1.71 ± 0.05
**4c**	3-Br	2.1 ± 0.07	39.9 ± 2.19	79.4 ± 4.76	
**4d**	3-I	2.0 ± 0.11	31.8 ± 1.59	67.5 ± 3.71	
**4e**	3-CH_3_	3.1 ± 0.12			
**4f**	3,5-diCH_3_	10.2 ± 0.61			
**4g**	3-OCH_3_	6.8 ± 0.24			
**4h**	3,5-diOCH_3_	34.3 ± 1.88			
**4i**	3-CH_2_CH_3_	2.6 ± 0.08			
**4j**	3-CH(CH_3_)_2_	2.4 ± 0.12	73.4 ± 4.77		
**4k**	3-NO_2_	5.9 ± 0.32			
**4l**	3,5-diBr	8.3 ± 0.4			
**4m**	3,5-diI	18.7 ± 0.93			
**5a**	4-F	1.5 ± 0.05	60.2 ± 2.7		
**5b**	H	1.5 ± 0.05	46.6 ± 1.63		
**5c**	2-CF_3_	5.5 ± 0.3			
**5d**	3,5-diCF_3_	14.7 ± 0.88			
**5e**	2,3,4-triF	1.0 ± 0.04	24.2 ± 1.45	38.9 ± 1.95	2.95 ± 0.13
**5f**	3,4-diF	3.0 ± 0.14			
**5g**	3-F	1.0 ± 0.04	1.0 ± 0.05	57.3 ± 3.44	1.69 ± 0.08
**5h**	2-CF_3_-4-F	2.4 ± 0.13	48.2 ± 3.37		
**5i**	4-OCF_3_	1.0 ± 0.03	9.5 ± 0.48	71.8 ± 4.67	
**5j**	4-CF_3_	2.9 ± 0.15			
**5k**	2-OCF_3_	15.5 ± 0.93			
**5l**	3,5-diF	1.0 ± 0.05	1.0 ± 0.06	13.6 ± 0.48	0.21 ± 0.01
**5m**	3-CF_3_	1.3 ± 0.06	59.9 ± 3.29		
**COUMATE**		51.8 ± 3.36			
**Irosustat**		2.4 ± 0.07	12.9 ± 0.77	16.8 ± 0.5	1.06 ± 0.03

a Substrate: [3H]E1S, 3 nM; experiments were carried out in triplicate.

Thus, the compounds most potent in MCF-7 cells at the initial concentration were assessed in the same assay at lower concentrations, which are 10 and 1 nM. At the 10 nM inhibitor concentration, the STS residual activity spanned from 1.0% (for compounds **5g** and **5l**) to 73.4% (**4j**). In comparison, 10 nM concentration **Irosustat** led to a 12.9% residual enzymatic action. The experiment at a 1 nM inhibitor concentration showed a notable STS residual activity of 13.6% after incubation with **5l**, even lower than the 16.8% produced by reference **Irosustat**. **5e** and **4a** also showed significant efficacy at 1 nM, with residual STS activities of 38.9 and 38.2%, respectively. The IC_50_ parameters were thus determined for the most potent derivatives. Relevantly, **4a**, **4b**, **5e**, **5g**, and **5l** demonstrated STS inhibitory potency comparable to or greater than that of **Irosustat**. In fact, **4a**, **4b**, **5e**, and **5g** showed IC_50_ values of 1.90, 1.71, 2.95, and 1.69 nM, respectively, that are comparable to that of 1.06 nM detected for *Irosustat*. **5l** Exhibited instead a 5-fold greater inhibitory potency than the reference, with an IC_50_ value of 0.21 nM. The results presented above indicate that the newly synthesized inhibitors were able to penetrate the cancer cells efficiently and inhibit STS. Additionally, a dependence between inhibitory efficacy and the type of halo-substituents was detected. Unlike results obtained from the radioisotope assay with isolated enzymes, derivatives including fluorine atoms in their structure demonstrated the greatest inhibitory action in cells.

### In Vivo Studies

#### Determination of the Maximum Tolerated Dose

Five among the most active and representative compounds, namely, **4a**, **4b**, **5e**, **5g**, and **5l**, were selected for in vivo studies. To determine the maximum tolerated dose (MTD), Balb/c mice (female, three mice for each dose of the compound) received *per os* (PO) compounds **4a**, **4b**, **5e**, **5g**, and **5l** at the doses of 10–20–50 mg/kg/day for 5 days a week for 2 weeks. The mice were thus weighed, and their general health was observed. No toxic effects of the compounds were observed, as well as no weight loss, with only slight changes in the consistency of the feces at higher doses (Figures S1–S3 and Table S1, Supporting Information). At the end of the study, the mice were sacrificed by dislocation of the cervical vertebrae, and the internal organs were examined macroscopically. During necropsy, no macroscopic changes in organs (liver, kidneys, intestines, and spleen) or in the weight of selected organs were observed (Figure S4, Supporting Information). For all tested compounds, the MTD was set to 50 mg/kg, administered *per os*, five times a week.

#### Antitumor Activity of STS Inhibitors in a 67NR Mouse Breast Carcinoma Model

**Figure 3:**
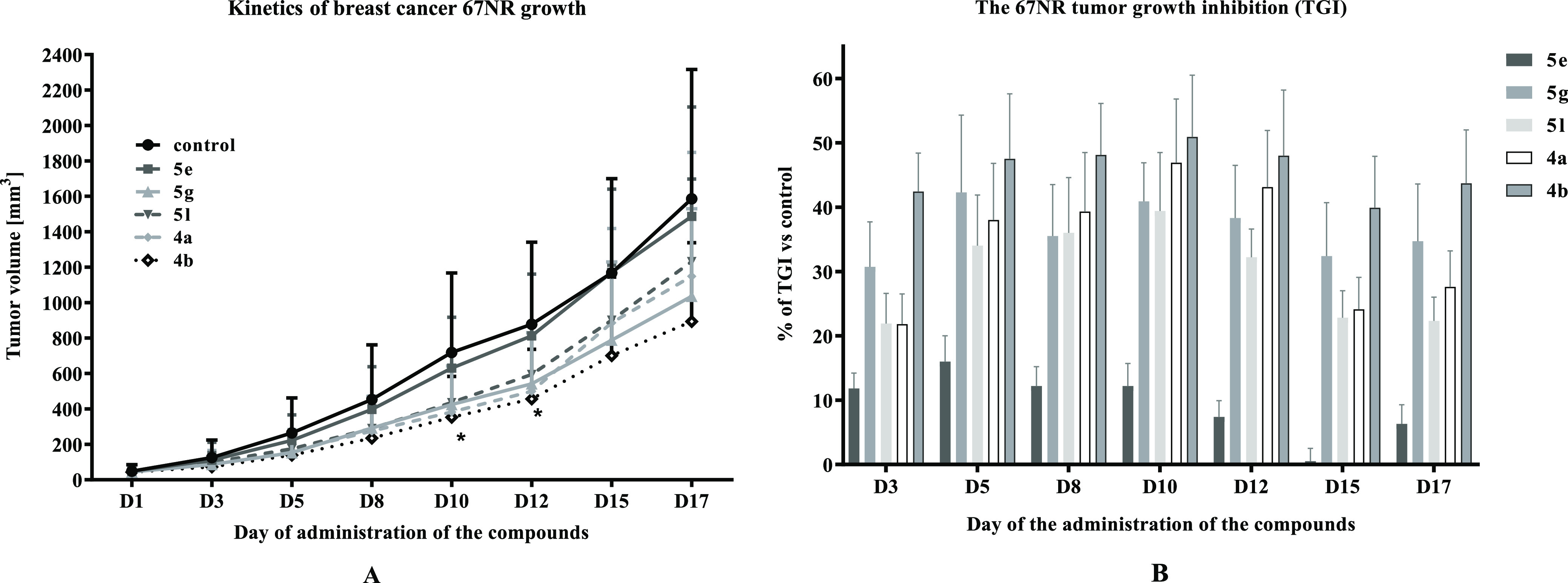
Kinetics of 67NR tumor growth (A) and TGI (B) in mice treated *per os* with tested compounds at the dose of 50 mg/kg b.w. *N* = 9; statistical analysis: one-way ANOVA and Dunnett multiple comparisons test. *p < 0.05 vs control group.

**Figure 4:**
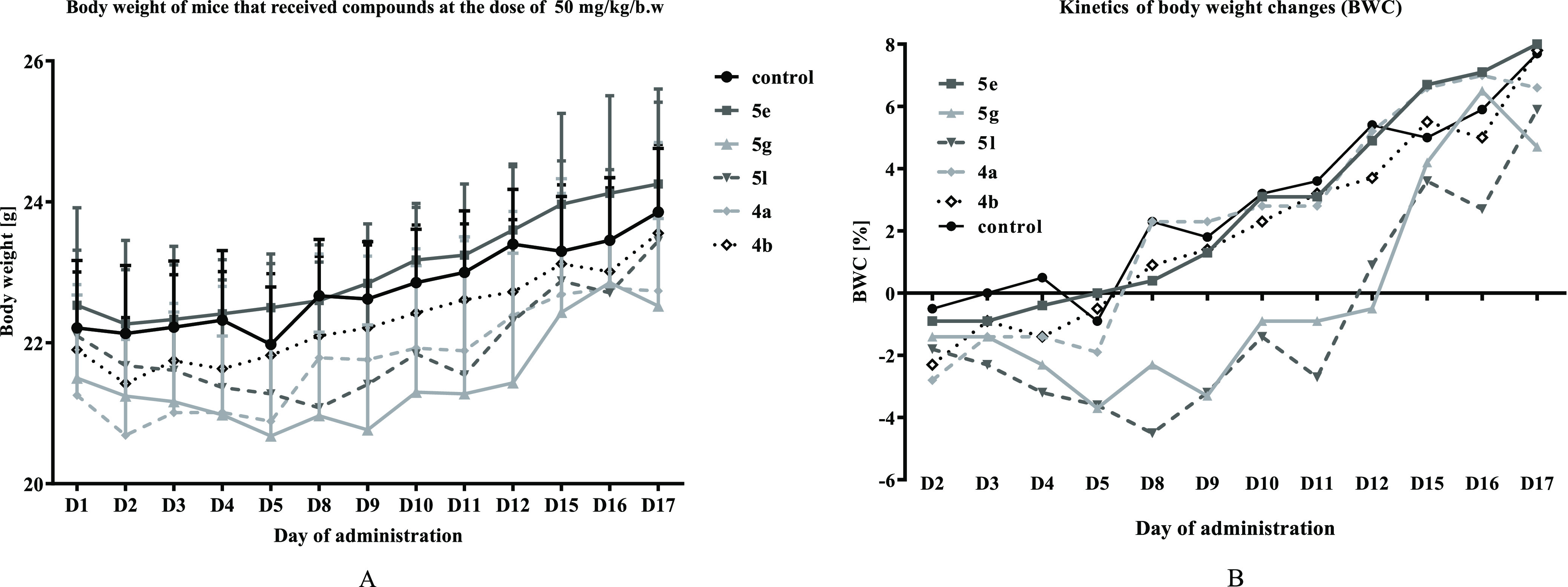
Body weight (A) and BWCs (B) of mice with breast 67NR tumors treated *per os* with tested compounds at the dose of 50 mg/kg b.w.

Mice were inoculated *orthotopically* (in the mammary gland fat pad) with 67NR mouse mammary tumor cells derived from in vitro culture. After the tumor growth to the average volume of 50 mm^3^, the mice were randomized into six groups, nine mice/group, and the *per os* administration of the tested compounds at the dose 50 mg/kg b.w. was started. The tumor volume (TV) and body weight were measured three times a week. Results for individual groups are reported in Figure S5, Supporting Information. Based on TV data, the tumor growth inhibition (TGI) was calculated for groups that received compounds and compared to that of the control group. **5e** did not show any significant effect on the growth of breast 67NR tumors. On the other hand, compounds **4a**, **4b**, **5g**, and **5l** showed significant antitumor activity, leading to TGI values of 47, 51, 42, and 39%, respectively (Figure 3). The analysis of individual tumors compared to the mean kinetics of the control group is summarized in Table 3. The body weight of the treated animals was monitored during the study, and the body weight change (BWC) index was calculated (Figure 4). Groups receiving **5g** and **5l** compounds showed small decrease of body weight, but only at the beginning of the administration. Body weight loss was observed between D2 and D11 and reached 4.5% at most.

**Table 3:**
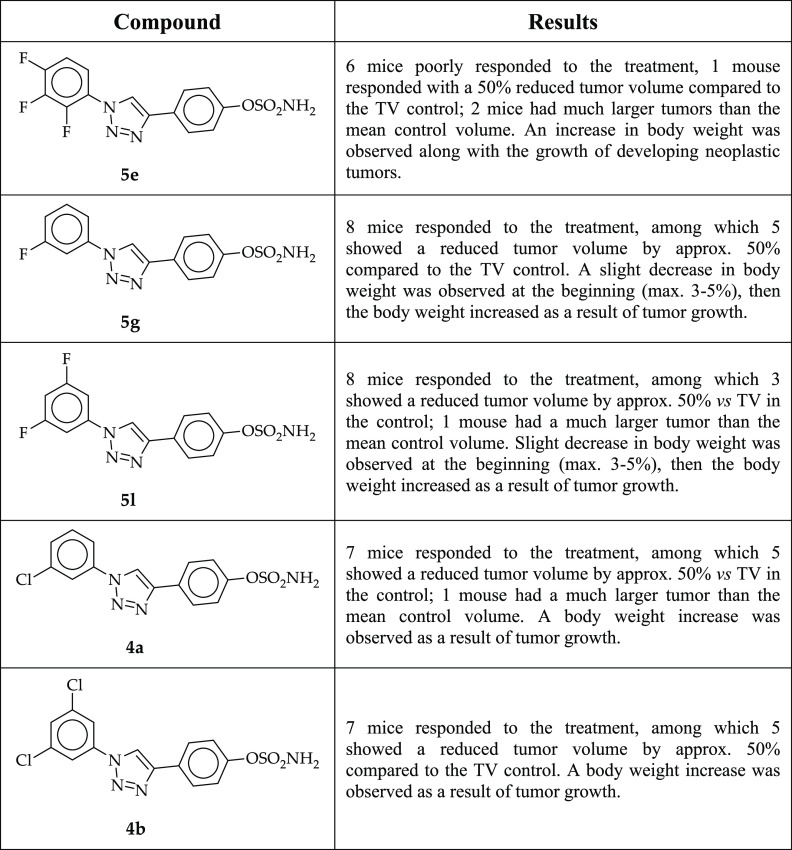
Summary of Results for TGI by STS Inhibitors in a 67NR Orthotropic Mouse Breast Carcinoma Model

At the end of the study, the autopsy of the animals was performed: blood was collected for further analyses of the morphology (Table S2, Supporting Information) and biochemistry (Table S3 and Figure S6, Supporting Information) and for the determination of the plasma estradiol level using the ELISA method. The internal organs were weighed and macroscopically assessed (Figure S7, Supporting Information). Tumors and liver tissue were also collected for the determination of STS activity.

Enlarged livers (not statistically significant) were observed in animals receiving *per os* compounds at the dose of 50 mg/kg b.w. (except for **4a**), which was associated with the increased levels of alanine aminotransferase (ALT) (significant in the **5g** and **5l** groups) and aspartate aminotransferase (AST) in the **5l** group (Table S3, Supporting Information). Significantly smaller spleens weights were observed in mice treated with **4a**, **4b**, **5g**, and **5l**, which was associated with a decrease in the total white blood cell (WBC) count, in particular, lymphocyte count (compared to that of control and tumor-free normal mice). All mice with tumor showed an increase in the number and percentage of monocytes and granulocytes (which is most often seen in inflammation and neoplastic diseases). Control mice had an increased total WBC count, which was associated with the developing inflammation that accompanied the neoplastic process. Slightly lower numbers of erythrocytes (RBCs) and slightly reduced levels of hemoglobin (HGB) and hematocrit (HCT) were observed in all mice compared to levels seen in normal mice. There were no differences between the control group and the treated groups, which may indicate that the tested compounds did not significantly affect the red blood cell system. Upon **5e** administration in the blood of mice, an increased level of platelets (PLT) was observed as compared to that of healthy and control mice. Blood biochemical tests also showed an increase in the urea level in the groups receiving the tested compounds.

**Figure 5:**
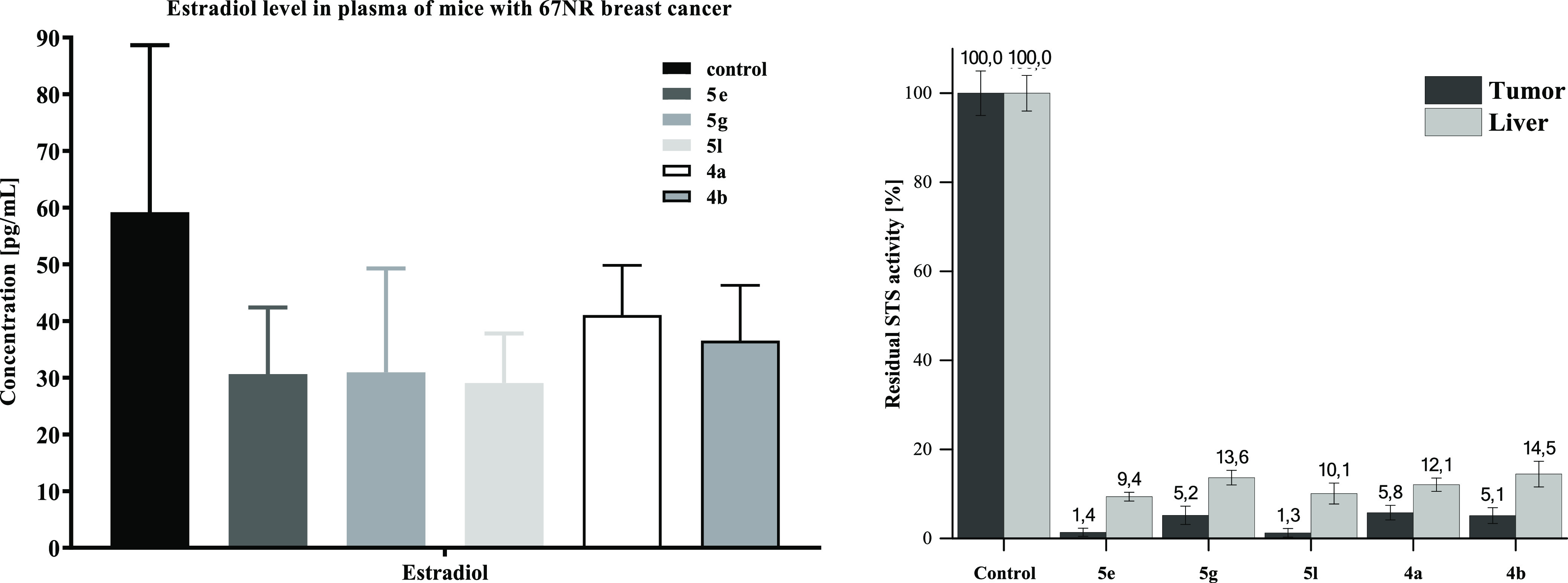
Level of estradiol in plasma of mice with 67NR tumor treated *per os* with tested compounds at the dose of 50 mg/kg b.w. (left chart) *N* = 9; statistical analysis: Mann–Whitney *U* test, *p < 0.05. Level of STS inhibition in collected tissues (right chart).

The analysis of blood plasma proved a reduction of the estrogen level (Figure 5) in all groups receiving tested compounds. Relevantly, higher levels of STS inhibition were measured in the collected tissues (tumor and liver), suggesting a main role of STS inhibition as a mechanism of action of such a beneficial therapeutic effect.

## Conclusions

In this work, we reported the development of a new series of 4-(1-phenyl-1*H*-1,2,3-triazol-4-yl)phenyl sulfamates as STS inhibitors. We had previously reported a set of analogues of such derivatives, among which compounds bearing fluorine atoms at the meta position of the outer aromatic ring exhibited the greatest STS inhibitory properties, prompting the series extension here reported by incorporation of a variety of m-substituents and guided by in silico analysis.

Primarily, the newly reported derivatives were assessed for their inhibition profile through a radioisotope enzymatic assay using STS isolated from human placenta. Chloro- (**4b**) and iodo-derivatives (**4d** and **4m**) exhibited the greatest in vitro inhibitory effect with residual STS activities of 13.32, 13.23, and 11.78%, respectively, at a 0.5 μM ligand concentration. Therefore, the STS inhibitory properties of compounds **4a–m** were assessed using a radioisotope assay in MCF-7 cells, which also included the previously reported derivatives **5a–m** and **COUMATE** and **Irosustat** as reference drugs. **5e**, **5g**, and **4a** demonstrated STS inhibitory potency comparable to that of **Irosustat**. Instead, **5l** was approximately 5-fold more potent than the standard drug reference in the cellular radioisotope assay. The five most active compounds, that are **4a**, **4b**, **5e**, **5g**, and **5l**, were subjected to in vivo studies for further evaluation, including (i) the MTD and (ii) their antitumor therapeutic action in a 67NR mouse breast carcinoma model. **5g**, **5l**, **4a**, and **4b** induced 42, 39, 47, and 51% inhibition of the tumor growth, respectively, at the dose of 50 mg/kg b.w. No side effects and toxicity were observed. The analysis of blood plasma proved a significant reduction of the estrogen level. Moreover, higher levels of STS inhibition were measured in the collected tissues (tumor and liver), suggesting a main role of STS inhibition as a mechanism of action of such a beneficial therapeutic effect.

## Methods

### Chemistry

Melting points (uncorrected) were determined using a Stuart Scientific SMP30 apparatus. Infrared (IR) spectra were recorded using a Nicolet 8700 spectrometer. ^1^H and ^13^C NMR spectra were recorded on a Bruker Avance III HD 400 MHz spectrometer. Chemical shifts (δ) are expressed in parts per million; coupling constants (*J*) are given in hertz. Mass spectra were recorded using an Agilent 6540 Accurate Mass quadrupole time-of-flight liquid chromatography mass spectrometry (LC/MS) system. Elemental analysis was performed using a CHNS-Carlo Erba EA-1108. Thin-layer chromatography (TLC) was performed using plates with Polygram SIL G/UV_254_ silica gel (Macherey–Nagel GmbH & Co. KG, Düren, Germany). Column chromatography was performed using silica gel 60 (230–400 mesh, Merck). Chromatographic analysis was performed using an Agilent liquid chromatograph series 1290 (Agilent Technology, Waldbronn, Germany) consisting of binary pump G4220A, autosampler G4226A, thermostated column compartment G1316C, and diode-array detector G1315C. The chromatographic system was controlled using Agilent MassHunter software B 06.01. All compounds are >95% pure according to high-performance LC (HPLC) analysis except for compound **4k** (not evaluated in vivo). The samples (2 μL) were injected onto a Poroshell EC-C18 2.7 μm (3.0 mm × 150 mm) column thermostated at 40 °C. The mobile phase flow rate was 0.4 mL min^–1^, and elution was performed using 0.1% (v/v) formic acid in water (solvent A) and ACN/MeOH (1:1; v/v) (solvent B) in the gradient mode: 10% B to 100% B in 30 min. The UV signal was registered at 254 nm. HPLC traces are reported in the Supporting Information.

Substrates for synthesis [the appropriate aniline derivatives, *t*-BuONO, TMSN_3_, 1 M solution of TBAF in THF, sodium ascorbate, CuSO_4_·5H_2_O, chlorosulfonyl isocyanate, *N*,*N*-dimethylacetamide (*N*,*N*-DMA), and formic acid] were commercially acquired from Sigma-Aldrich. Solvents [ACN, dichloromethane (DCM), ethyl acetate (AcOEt)] were dried and distilled using standard procedures. 4-(Trimethylsilyl)ethynyl)phenol was obtained according to the previously described synthetic procedure (ref (32)).

### General Procedure for the Synthesis of 4-(1-Phenyl-1*H*-1,2,3-triazol-4-yl)phenol Derivatives **3a–m**

The corresponding amine **1** (2.63 mmol) was dissolved in ACN (6.1 mL), and the obtained solution was cooled in an ice water bath. Then, *t*-BuONO (0.325 g, 3.16 mmol) was added dropwise, followed by the addition of TMSN_3_ (0.333 g, 2.89 mmol). The solution was stirred at room temperature (RT) for 4 h. In the next step, 4-(trimethylsilyl)ethynyl)phenol (0.5 g, 2.63 mmol) and 1 M solution of TBAF in THF (2.89 mL) were added, and the reaction mixture was stirred at 0 °C for 30 min. Then, CuSO_4_·5H_2_O (65.7 mg, 0.263 mmol) and a freshly prepared aqueous solution (0.526 mL) of sodium ascorbate (0.104 g, 0.526 mmol) were added, and the obtained solution was stirred for 24 h under an argon atmosphere at RT. The next day, the reaction mixture was concentrated under vacuum. The crude product was dissolved in AcOEt (30 mL), and the solution was washed with 0.1 M hydrochloric acid. After separation, the organic layer was dried, and the solvent was evaporated. The resulting residue was recrystallized from ACN to give the desired products **3a–m**.

#### 4-(1-(3-Chlorophenyl)-1*H*-1,2,3-triazol-4-yl)phenol **3a**

Yield: 70%; mp: 208–209 °C; ν_max_ (KBr)/cm^–1^: 3458, 1616, 1591, 1466, 1222, 1175, 1040, 839, 681; ^1^H NMR δ_H_ (400 MHz, DMSO): 9.70 (1H, s, OH), 9.19 (1H, s, CH), 8.07 (1H, t, *J* = 2.0 Hz, Ar–H), 7.98–7.93 (1H, m, Ar–H), 7.75 (2H, d, *J* = 8.7 Hz, Ar–H), 7.66 (1H, t, *J* = 8.1 Hz, Ar–H), 7.60–7.55 (1H, m, Ar–H), 6.89 (2H, d, *J* = 8.7 Hz, Ar–H); ^13^C NMR δ_C_ (101 MHz, DMSO): 158.2, 148.3, 138.2, 134.7, 132.1, 128.8, 127.3, 121.4, 120.1, 118.9, 118.7, 116.2. Anal. Calcd for: C_14_H_10_ClN_3_O: C, 61.89; H, 3.71; N, 15.47%. Found: C, 61.97; H, 3.60; N, 15.51%. HRMS (*m*/*z*): [M – H]^−^ calcd, 270.0434; found, 270.0547.

#### 4-(1-(3,5-Dichlorophenyl)-1*H*-1,2,3-triazol-4-yl)phenol **3b**

Yield: 54%; mp: 241–244 °C; ν_max_ (KBr)/cm^–1^: 3126, 1614, 1591, 1471, 1226, 1177, 1057, 841, 662; ^1^H NMR δ_H_ (400 MHz, DMSO): 9.72 (1H, s, OH), 9.24 (1H, s, CH), 8.08 (2H, d, *J* = 1.8 Hz, Ar–H), 7.76 (1H, t, *J* = 1.8 Hz, Ar–H), 7.73 (2H, d, *J* = 8.7 Hz, Ar–H), 6.89 (2H, d, *J* = 8.7 Hz, Ar–H); ^13^C NMR δ_C_ (101 MHz, DMSO): 158.3, 148.4, 138.8, 138.7, 135.7, 128.3, 127.3, 121.2, 118.9, 116.3. Anal. Calcd for: C_14_H_9_Cl_2_N_3_O: C, 54.92; H 2.96; N, 13.73%. Found: C, 54.85; H, 2.91; N, 13.86%. HRMS (*m*/*z*): [M – H]^−^ calcd, 304.0044; found, 304.0156.

#### 4-(1-(3-Bromophenyl)-1*H*-1,2,3-triazol-4-yl)phenol **3c**

Yield: 78%; mp: 200–202 °C; ν_max_ (KBr)/cm^–1^: 3457, 1616, 1594, 1480, 1221, 1176, 1035, 839, 682; ^1^H NMR δ_H_ (400 MHz, DMSO): 9.69 (1H, s, OH), 9.20 (1H, s, CH), 8.20 (1H, t, *J* = 1.9 Hz, Ar–H), 8.03–7.97 (1H, m, Ar–H), 7.75 (2H, d, *J* = 8.6 Hz, Ar–H), 7.73–7.68 (1H, m, Ar–H), 7.59 (1H, t, *J* = 8.1 Hz, Ar–H), 6.89 (2H, d, *J* = 8.7 Hz, Ar–H); ^13^C NMR δ_C_ (101 MHz, DMSO): 158.2, 148.3, 138.3, 132.4, 131.7, 127.3, 122.9, 122.8, 121.4, 119.3, 118.7, 116.2. Anal. Calcd for: C_14_H_10_BrN_3_O: C, 53.19; H 3.19; N, 13.29%. Found: C, 53.25; H, 3.12; N, 13.22%. HRMS (*m*/*z*): [M – H]^−^ calcd, 313.9929; found, 314.0044.

#### 4-(1-(3,5-Dibromophenyl)-1*H*-1,2,3-triazol-4-yl)phenol **3d**

Yield: 48%; mp: 222–226 °C (with decomposition); ν_max_ (KBr)/cm^–1^: 3120, 1616, 1586, 1502, 1231, 1173, 1056, 839, 662; ^1^H NMR δ_H_ (400 MHz, DMSO): 9.72 (1H, s, OH), 9.25 (1H, s, CH), 8.24 (2H, d, *J* = 1.7 Hz, Ar–H), 7.99 (1H, t, *J* = 1.6 Hz, Ar–H), 7.73 (2H, d, *J* = 8.7 Hz, Ar–H), 6.89 (2H, d, *J* = 8.7 Hz, Ar–H); ^13^C NMR δ_C_ (101 MHz, DMSO): 158.2, 148.3, 139.0, 133.6, 127.3, 123.9, 122.0, 121.2, 118.9, 116.3. Anal. Calcd for: C_14_H_9_Br_2_N_3_O: C, 42.56; H 2.30; N, 10.64%. Found: C, 42.49; H, 2.21; N, 10.78%. HRMS (*m*/*z*): [M – H]^−^ calcd, 393.9014; found, 393.9142.

#### 4-(1-(3-Iodophenyl)-1*H*-1,2,3-triazol-4-yl)phenol **3e**

Yield: 73%; mp: 190–194 °C; ν_max_ (KBr)/cm^–1^: 3064, 1616, 1583, 1480, 1224, 1171, 1055, 841, 671; ^1^H NMR δ_H_ (400 MHz, DMSO): 9.69 (1H, s, OH), 9.18 (1H, s, CH), 8.33 (1H, t, *J* = 1.8 Hz, Ar–H), 8.02–7.97 (1H, m, Ar–H), 7.89–7.84 (1H, m, Ar–H), 7.75 (2H, d, *J* = 8.6 Hz, Ar–H), 7.41 (1H, t, *J* = 8.0 Hz, Ar–H), 6.89 (2H, d, *J* = 8.7 Hz, Ar–H); ^13^C NMR δ_C_ (101 MHz, DMSO): 158.1, 148.2, 138.1, 137.6, 132.2, 128.3, 127.3, 121.5, 119.6, 118.7, 116.2, 95.9. Anal. Calcd for: C_14_H_10_IN_3_O: C, 46.30; H 2.78; N, 11.57%. Found: C, 46.37; H, 2.69; N, 11.42%. HRMS (*m*/*z*): [M – H]^−^ calcd, 361.9790; found, 361.9930.

#### 4-(1-(3,5-Diiodophenyl)-1*H*-1,2,3-triazol-4-yl)phenol **3f**

Yield: 56%; mp: 241–243 °C (with decomposition); ν_max_ (KBr)/cm^–1^: 3122, 1614, 1572, 1500, 1225, 1169, 1050, 838, 663; ^1^H NMR δ_H_ (400 MHz, DMSO): 9.71 (1H, s, OH), 9.22 (1H, s, CH), 8.35 (2H, d, *J* = 1.4 Hz, Ar–H), 8.21 (1H, t, *J* = 1.4 Hz, Ar–H), 7.73 (2H, d, *J* = 8.7 Hz, Ar–H), 6.88 (2H, d, *J* = 8.7 Hz, Ar–H); ^13^C NMR δ_C_ (101 MHz, DMSO): 158.2, 148.3, 144.5, 138.5, 127.7, 127.3, 121.3, 118.8, 116.2, 97.3. Anal. Calcd for: C_14_H_9_I_2_N_3_O: C, 34.38; H 1.85; N, 8.59%. Found: C, 34.27; H, 1.94; N, 8.72%. HRMS (*m*/*z*): [M – H]^−^ calcd, 487.8757; found, 487.8908.

#### 4-(1-(3-Methylphenyl)-1*H*-1,2,3-triazol-4-yl)phenol **3g**

Yield: 70%; mp: 215–218 °C; ν_max_ (KBr)/cm^–1^: 3071, 1614, 1592, 1486, 1220, 1174, 1064, 842, 678; ^1^H NMR δ_H_ (400 MHz, DMSO): 9.67 (1H, s, OH), 9.09 (1H, s, CH), 7.80–7.71 (4H, m, Ar–H), 7.50 (1H, t, *J* = 7.8 Hz, Ar–H), 7.32 (1H, d, *J* = 7.6 Hz, Ar–H), 6.88 (2H, d, *J* = 8.7 Hz, Ar–H), 2.44 (3H, s, CH_3_); ^13^C NMR δ_C_ (101 MHz, DMSO): 158.0, 148.0, 140.1, 137.2, 130.2, 129.6, 127.2, 121.7, 120.7, 118.5, 117.4, 116.2, 21.4. Anal. Calcd for: C_15_H_13_N_3_O: C, 71.70; H 5.21; N, 16.72%. Found: C, 71.85; H, 5.14; N, 16.79%. HRMS (*m*/*z*): [M – H]^−^ calcd, 250.0980; found, 250.1126.

#### 4-(1-(3,5-Dimethylphenyl)-1*H*-1,2,3-triazol-4-yl)phenol **3h**

Yield: 70%; mp: 235–238 °C; ν_max_ (KBr)/cm^–1^: 3021, 1618, 1592, 1487, 1213, 1172, 1069, 838, 676; ^1^H NMR δ_H_ (400 MHz, DMSO): 9.66 (1H, s, OH), 9.06 (1H, s, CH), 7.75 (2H, d, *J* = 8.6 Hz, Ar–H), 7.57 (2H, s, Ar–H), 7.13 (1H, s, Ar–H), 6.88 (2H, d, *J* = 8.7 Hz, Ar–H), 2.39 (6H, s, CH_3_); ^13^C NMR δ_C_ (101 MHz, DMSO): 158.0, 148.0, 139.8, 137.1, 130.3, 127.2, 121.7, 118.5, 117.9, 116.2, 21.3. Anal. Calcd for: C_16_H_15_N_3_O: C, 72.43; H 5.70; N, 15.84%. Found: C, 72.33; H, 5.63; N, 15.99%. HRMS (*m*/*z*): [M – H]^−^ calcd, 264.1137; found, 264.1283.

#### 4-(1-(3-Methoxyphenyl)-1*H*-1,2,3-triazol-4-yl)phenol **3i**

Yield: 76%; mp: 231–233 °C; ν_max_ (KBr)/cm^–1^: 3076, 1610, 1594, 1489, 1225, 1167, 1064, 837, 679; ^1^H NMR δ_H_ (400 MHz, DMSO): 9.67 (1H, s, OH), 9.13 (1H, s, CH), 7.76 (2H, d, *J* = 8.6 Hz, Ar–H), 7.55–7.50 (3H, m, Ar–H), 7.10–7.04 (1H, m, Ar–H), 6.89 (2H, d, *J* = 8.7 Hz, Ar–H), 3.88 (3H, s, CH_3_); ^13^C NMR δ_C_ (101 MHz, DMSO): 160.7, 158.1, 148.1, 138.2, 131.3, 127.3, 121.6, 118.7, 116.2, 114.7, 112.3, 105.9, 56.1. Anal. Calcd for: C_15_H_13_N_3_O_2_: C, 67.40; H 4.90; N, 15.72%. Found: C, 67.25; H, 4.99; N, 15.79%. HRMS (*m*/*z*): [M – H]^−^ calcd, 266.0930; found, 266.1076.

#### 4-(1-(3,5-Dimethoxyphenyl)-1*H*-1,2,3-triazol-4-yl)phenol **3j**

Yield: 35%; mp: 190–193 °C; ν_max_ (KBr)/cm^–1^: 3120, 1615, 1593, 1493, 1231, 1153, 1062, 830, 676; ^1^H NMR δ_H_ (400 MHz, DMSO): 9.67 (1H, s, OH), 9.13 (1H, s, CH), 7.75 (2H, d, *J* = 8.7 Hz, Ar–H), 7.13 (2H, d, *J* = 2.2 Hz, Ar–H), 6.89 (2H, d, *J* = 8.7 Hz, Ar–H), 6.62 (1H, t, *J* = 2.2 Hz, Ar–H), 3.86 (6H, s, CH_3_); ^13^C NMR δ_C_ (101 MHz, DMSO): 161.7, 158.1, 148.0, 138.7, 127.3, 121.6, 118.7, 116.2, 100.6, 98.6, 56.2. Anal. Calcd for: C_16_H_15_N_3_O_3_: C, 64.64; H 5.09; N, 14.13%. Found: C, 64.77; H, 5.05; N, 14.06%. HRMS (*m*/*z*): [M – H]^−^ calcd, 296.1035; found, 296.1189.

#### 4-(1-(3-Ethylphenyl)-1*H*-1,2,3-triazol-4-yl)phenol **3k**

Yield: 70%; mp: 188–189 °C; ν_max_ (KBr)/cm^–1^: 3148, 1611, 1591, 1498, 1233, 1177, 1069, 833, 689; ^1^H NMR δ_H_ (400 MHz, DMSO): 9.67 (1H, s, OH), 9.11 (1H, s, CH), 7.82–7.72 (4H, m, Ar–H), 7.52 (1H, t, *J* = 7.8 Hz, Ar–H), 7.35 (1H, d, *J* = 7.6 Hz, Ar–H), 6.89 (2H, d, *J* = 8.7 Hz, Ar–H), 2.74 (2H, q, *J* = 7.6 Hz, CH_2_), 1.25 (3H, t, *J* = 7.6 Hz, CH_3_); ^13^C NMR δ_C_ (101 MHz, DMSO): 158.0, 148.0, 146.4, 137.2, 130.1, 128.4, 127.3, 121.7, 119.6, 118.6, 117.7, 116.2, 28.5, 15.9. Anal. Calcd for: C_16_H_15_N_3_O: C, 72.43; H 5.70; N, 15.84%. Found: C, 72.50 H, 5.79; N, 15.67%. HRMS (*m*/*z*): [M – H]^−^ calcd, 264.1137; found, 264.1285.

#### 4-(1-(3-Isopropylphenyl)-1*H*-1,2,3-triazol-4-yl)phenol **3l**

Yield: 78%; mp: 153–155 °C; ν_max_ (KBr)/cm^–1^: 3118, 1613, 1591, 1495, 1224, 1173, 1064, 845, 664; ^1^H NMR δ_H_ (400 MHz, DMSO): 9.67 (1H, s, OH), 9.13 (1H, s, CH), 7.83–7.73 (4H, m, Ar–H), 7.53 (1H, t, *J* = 7.9 Hz, Ar–H), 7.38 (1H, d, *J* = 7.7 Hz, Ar–H), 6.89 (2H, d, *J* = 8.7 Hz, Ar–H), 3.03 (1H, hept, *J* = 6.9 Hz, CH), 1.29 (6H, d, *J* = 6.9 Hz, CH_3_); ^13^C NMR δ_C_ (101 MHz, DMSO): 158.0, 151.0, 148.0, 137.3, 130.3, 127.3, 127.1, 121.7, 118.6, 118.3, 117.9, 116.2, 33.9, 24.2. Anal. Calcd for: C_17_H_17_N_3_O: C, 73.10; H 6.13; N, 15.04%. Found: C, 72.99; H, 6.17; N, 15.15%. HRMS (*m*/*z*): [M – H]^−^ calcd, 278.1293; found, 278.1444.

#### 4-(1-(3-Nitrophenyl)-1*H*-1,2,3-triazol-4-yl)phenol **3m**

Yield: 62%; mp: 267–268 °C (with decomposition); ν_max_ (KBr)/cm^–1^: 3265, 1615, 1592, 1493, 1229, 1173, 1048, 836, 663; ^1^H NMR δ_H_ (400 MHz, DMSO): 9.71 (1H, s, OH), 9.37 (1H, s, CH), 8.76 (1H, t, *J* = 2.1 Hz, Ar–H), 8.44 (1H, dd, *J* = 8.1, 2.1 Hz, Ar–H), 8.33 (1H, dd, *J* = 8.3, 2.2 Hz, Ar–H), 7.93 (1H, t, *J* = 8.2 Hz, Ar–H), 7.77 (2H, d, *J* = 8.6 Hz, Ar–H), 6.90 (2H, d, *J* = 8.7 Hz, Ar–H); ^13^C NMR δ_C_ (101 MHz, DMSO): 158.2, 149.0, 148.5, 137.8, 132.0, 127.3, 126.2, 123.4, 121.3, 119.0, 116.3, 114.8. Anal. Calcd for: C_14_H_10_N_4_O_3_: C, 59.57; H 3.57; N, 19.85%. Found: C, 59.66; H, 3.46; N, 19.91%. HRMS (*m*/*z*): [M – H]^−^ calcd, 281.0675; found, 281.0821.

### General Procedure for the Synthesis of 4-(1-Phenyl-1*H*-1,2,3-triazol-4-yl)phenyl Sulfamate Derivatives **4a–m**

To a solution of chlorosulfonyl isocyanate (212 mg, 1.50 mmol) in dry DCM (0.5 mL), a mixture of formic acid (70.9 mg, 1.54 mmol) and *N*,*N*-DMA (1.4 mg, 0.016 mmol) was added, and the obtained solution was stirred at 40 °C for 3.5 h. In the next step, a solution of the corresponding derivative **3a–m** (1.00 mmol) in *N*,*N*-DMA (3.4 mL) was added, and the obtained solution was stirred at RT overnight. The next day, the mixture was poured into water (50 mL). The precipitated solid was filtered, washed with water, dried, and purified using preparative column chromatography with DCM/AcOEt (1:1) as an eluent to give the desired products **4a–m**.

#### 4-(1-(3-Chlorophenyl)-1*H*-1,2,3-triazol-4-yl)phenyl Sulfamate **4a**

Yield: 80%; mp: 224–225 °C (with decomposition); ν_max_ (KBr)/cm^–1^: 3354, 1597, 1489, 1377, 1177, 1153, 1060, 936, 864, 729, 676; ^1^H NMR δ_H_ (400 MHz, DMSO): 9.42 (1H, s, CH), 8.13–8.05 (3H, m, NH_2_, Ar–H),8.05–7.96 (3H, m, Ar–H), 7.69 (1H, t, *J* = 8.1 Hz, Ar–H), 7.64–7.57 (1H, m, Ar–H), 7.43 (2H, d, *J* = 8.8 Hz, Ar–H); ^13^C NMR δ_C_ (101 MHz, DMSO): 150.5, 147.1, 138.1, 134.7, 132.2, 129.1, 128.9, 127.2, 123.4, 120.5, 120.3, 119.1. Anal. Calcd for: C_14_H_11_ClN_4_O_3_S: C, 47.94; H, 3.16; N, 15.97; S, 9.14%. Found: C, 48.01; H, 3.22; N, 15.85; S, 9.09%. HRMS (*m*/*z*): [M – H]^−^ calcd, 349.0162; found, 349.0268.

#### 4-(1-(3,5-Dichlorophenyl)-1*H*-1,2,3-triazol-4-yl)phenyl Sulfamate **4b**

Yield: 57%; mp: 237–238 °C (with decomposition); ν_max_ (KBr)/cm^–1^: 3336, 1586, 1477, 1373, 1178, 1158, 1058, 949, 872, 728, 664; ^1^H NMR δ_H_ (400 MHz, DMSO): 9.46 (1H, s, CH), 8.13–8.05 (4H, m, NH_2_, Ar–H), 7.99 (2H, d, *J* = 8.7 Hz, Ar–H), 7.81 (1H, t, *J* = 1.8 Hz, Ar–H), 7.44 (2H, d, *J* = 8.7 Hz, Ar–H); ^13^C NMR δ_C_ (101 MHz, DMSO): 150.6, 147.1, 138.6, 135.8, 128.7, 128.6, 127.2, 123.4, 120.7, 119.1. Anal. Calcd for C_14_H_10_Cl_2_N_4_O_3_S: C, 43.65; H 2.62; N, 14.54; S, 8.32%. Found: C, 43.52; H, 2.69; N, 14.64; S, 8.40%. HRMS (*m*/*z*): [M – H]^−^ calcd, 382.9772; found, 382.9878.

#### 4-(1-(3-Bromophenyl)-1*H*-1,2,3-triazol-4-yl)phenyl Sulfamate **4c**

Yield: 83%; mp: 212–213 °C (with decomposition); ν_max_ (KBr)/cm^–1^: 3319, 1589, 1484, 1371, 1177, 1156, 1053, 951, 874, 730, 674; ^1^H NMR δ_H_ (400 MHz, DMSO): 9.42 (1H, s, CH), 8.22 (1H, t, *J* = 1.9 Hz, Ar–H), 8.09 (2H, s, NH_2_), 8.04–7.99 (3H, m, Ar–H), 7.76–7.71 (1H, m, Ar–H), 7.61 (1H, t, *J* = 8.1 Hz, Ar–H), 7.43 (2H, d, *J* = 8.8 Hz, Ar–H); ^13^C NMR δ_C_ (101 MHz, DMSO): 150.5, 147.1, 138.1, 132.4, 132.0, 128.9, 127.2, 123.4, 123.0, 122.9, 120.5, 119.4. Anal. Calcd for C_14_H_11_BrN_4_O_3_S: C, 42.54; H 2.81; N, 14.18; S, 8.11%. Found: C, 42.63; H, 2.87; N, 14.22; S, 8.02%. HRMS (*m*/*z*): [M – H]^−^ calcd, 392.9657; found, 392.9766.

#### 4-(1-(3-Iodophenyl)-1*H*-1,2,3-triazol-4-yl)phenyl Sulfamate **4d**

Yield: 73%; mp: 228–229 °C (with decomposition); ν_max_ (KBr)/cm^–1^: 3319, 1584, 1480, 1371, 1178, 1155, 1050, 951, 876, 759, 676; ^1^H NMR δ_H_ (400 MHz, DMSO): 9.40 (1H, s, CH), 8.35 (1H, t, *J* = 1.8 Hz, Ar–H), 8.09 (2H, s, NH_2_), 8.05–7.97 (3H, m, Ar–H), 7.92–7.86 (1H, m, Ar–H), 7.47–7.39 (3H, m, Ar–H); ^13^C NMR δ_C_ (101 MHz, DMSO): 150.5, 147.0, 137.9, 137.8, 132.3, 129.0, 128.5, 127.2, 123.3, 120.4, 119.8, 96.0. Anal. Calcd for: C_14_H_11_IN_4_O_3_S: C, 38.02; H 2.51; N, 12.67; S, 7.25%. Found: C, 37.89; H, 2.55; N, 12.61; S, 7.41%. HRMS (*m*/*z*): [M – H]^−^ calcd, 440.9518; found, 440.9654.

#### 4-(1-(3-Methylphenyl)-1*H*-1,2,3-triazol-4-yl)phenyl Sulfamate **4e**

Yield: 52%; mp: 212–213 °C (with decomposition); ν_max_ (KBr)/cm^–1^: 3340, 1595, 1494, 1373, 1174, 1152, 1039, 947, 867, 759, 686; ^1^H NMR δ_H_ (400 MHz, DMSO): 9.32 (1H, s, CH), 8.08 (2H, s, NH_2_), 8.03 (2H, d, *J* = 8.7 Hz, Ar–H), 7.80 (1H, s, Ar–H), 7.76 (1H, d, *J* = 8.3 Hz, Ar–H), 7.52 (1H, t, *J* = 7.8 Hz, Ar–H), 7.42 (2H, d, *J* = 8.7 Hz, Ar–H), 7.35 (1H, d, *J* = 7.6 Hz, Ar–H), 2.45 (3H, s, CH_3_); ^13^C NMR δ_C_ (101 MHz, DMSO): 150.4, 146.9, 140.2, 137.0, 130.2, 129.9, 129.2, 127.1, 123.3, 120.9, 120.3, 117.6, 21.4. Anal. Calcd for: C_15_H_14_N_4_O_3_S: C, 54.53; H 4.27; N, 16.96; S, 9.71%. Found: C, 54.44; H, 4.19; N, 17.08; S, 9.83%. HRMS (*m*/*z*): [M – H]^−^ calcd, 329.0708; found, 329.0851.

#### 4-(1-(3,5-Dimethylphenyl)-1*H*-1,2,3-triazol-4-yl)phenyl Sulfamate **4f**

Yield: 76%; m:p 234–238 °C (with decomposition); ν_max_ (KBr)/cm^–1^: 3332, 1591, 1489, 1366, 1176, 1153, 1061, 948, 871, 758, 679; ^1^H NMR δ_H_ (400 MHz, DMSO): 9.30 (1H, s, CH), 8.08 (2H, s, NH_2_), 8.02 (2H, d, *J* = 8.8 Hz, Ar–H), 7.59 (2H, s, Ar–H), 7.42 (2H, d, *J* = 8.8 Hz, Ar–H), 7.16 (1H, s, Ar–H), 2.40 (6H, s, CH_3_); ^13^C NMR δ_C_ (101 MHz, DMSO): 150.4, 146.8, 139.9, 137.0, 130.5, 129.2, 127.1, 123.3, 120.2, 118.0, 21.4. Anal. Calcd for: C_16_H_16_N_4_O_3_S: C, 55.80; H 4.68; N, 16.27; S, 9.31%. Found: C, 55.87; H, 4.75; N, 16.13; S, 9.19%. HRMS (*m*/*z*): [M – H]^−^ calcd, 343.0865; found, 343.1014.

#### 4-(1-(3-Methoxyphenyl)-1*H*-1,2,3-triazol-4-yl)phenyl Sulfamate **4g**

Yield: 64%; mp: 210–212 °C (with decomposition); ν_max_ (KBr)/cm^–1^: 3298, 1608, 1483, 1370, 1177, 1153, 1061, 953, 871, 760, 681; ^1^H NMR δ_H_ (400 MHz, DMSO): 9.35 (1H, s, CH), 8.08 (2H, s, NH_2_), 8.02 (2H, d, *J* = 8.7 Hz, Ar–H), 7.57–7.51 (3H, m, Ar–H), 7.43 (2H, d, *J* = 8.7 Hz, Ar–H), 7.13–7.07 (1H, m, Ar–H), 3.89 (3H, s, CH_3_); ^13^C NMR δ_C_ (101 MHz, DMSO): 160.7, 150.4, 146.9, 138.1, 131.4, 129.1, 127.1, 123.3, 120.4, 114.9, 112.4, 106.1, 56.1. Anal. Calcd for: C_15_H_14_N_4_O_4_S: C, 52.02; H 4.07; N, 16.18; S, 9.26%. Found: C, 51.96 H, 3.99; N, 16.30; S, 9.39%. HRMS (*m*/*z*): [M – H]^−^ calcd, 345.0658; found, 345.0810.

#### 4-(1-(3,5-Dimethoxyphenyl)-1*H*-1,2,3-triazol-4-yl)phenyl Sulfamate **4h**

Yield: 59%; mp: 225–227 °C (with decomposition); ν_max_ (KBr)/cm^–1^: 3331, 1597, 1480, 1373, 1178, 1152, 1068, 949, 874, 759, 676; ^1^H NMR δ_H_ (400 MHz, DMSO): 9.35 (1H, s, CH), 8.08 (2H, s, NH_2_), 8.01 (2H, d, *J* = 8.7 Hz, Ar–H), 7.43 (2H, d, *J* = 8.7 Hz, Ar–H), 7.15 (2H, d, *J* = 2.2 Hz, Ar–H), 6.65 (1H, t, *J* = 2.2 Hz, Ar–H), 3.87 (6H, s, CH_3_); ^13^C NMR δ_C_ (101 MHz, DMSO): 161.7, 150.4, 146.8, 138.5, 129.1, 127.1, 123.3, 120.4, 100.8, 98.7, 56.2. Anal. Calcd for: C_16_H_16_N_4_O_5_S: C, 51.06; H 4.28; N, 14.89; S, 8.52%. Found: C, 51.17; H, 4.19; N, 14.98; S, 8.67%. HRMS (*m*/*z*): [M – H]^−^ calcd, 375.0763; found, 375.0919.

#### 4-(1-(3-Ethylphenyl)-1*H*-1,2,3-triazol-4-yl)phenyl Sulfamate **4i**

Yield: 68%; mp: 223–225 °C (with decomposition); ν_max_ (KBr)/cm^–1^: 3314, 1589, 1484, 1371, 1178, 1154, 1053, 951, 874, 760, 692; ^1^H NMR δ_H_ (400 MHz, DMSO): 9.33 (1H, s, CH), 8.08 (2H, s, NH_2_), 8.03 (2H, d, *J* = 8.8 Hz, Ar–H), 7.83–7.74 (2H, m, Ar–H), 7.55 (1H, t, *J* = 7.8 Hz, Ar–H), 7.43 (2H, d, *J* = 8.8 Hz, Ar–H), 7.38 (1H, d, *J* = 8.2 Hz, Ar–H), 2.75 (2H, q, *J* = 7.6 Hz, CH_2_), 1.27 (3H, t, *J* = 7.6 Hz, CH_3_); ^13^C NMR δ_C_ (101 MHz, DMSO): 150.4, 146.9, 146.4, 137.1, 130.3, 129.2, 128.7, 127.1, 123.3, 120.3, 119.8, 117.9, 28.5, 15.9. Anal. Calcd for: C_16_H_16_N_4_O_3_S: C, 55.80; H 4.68; N, 16.27; S, 9.31%. Found: C, 55.66; H, 4.81; N, 16.33; S, 9.24%. HRMS (*m*/*z*): [M – H]^−^ calcd, 343.0865; found, 343.1020.

#### 4-(1-(3-Isopropylphenyl)-1*H*-1,2,3-triazol-4-yl)phenyl Sulfamate **4j**

Yield: 65%; mp: 202–204 °C (with decomposition); ν_max_ (KBr)/cm^–1^: 3335, 1584, 1486, 1372, 1175, 1153, 1055, 942, 869, 758, 692; ^1^H NMR δ_H_ (400 MHz, DMSO): 9.34 (1H, s, CH), 8.08 (2H, s, NH_2_), 8.04 (2H, d, *J* = 8.8 Hz, Ar–H), 7.83 (1H, t, *J* = 1.9 Hz, Ar–H), 7.80–7.75 (1H, m, Ar–H), 7.55 (1H, t, *J* = 7.9 Hz, Ar–H), 7.46–7.38 (3H, m, Ar–H), 3.04 (1H, hept, *J* = 6.9 Hz, CH), 1.29 (6H, d, *J* = 6.9 Hz, CH_3_); ^13^C NMR δ_C_ (101 MHz, DMSO): 151.1, 150.4, 146.9, 137.1, 130.4, 129.2, 127.3, 127.1, 123.3, 120.3, 118.4, 118.1, 33.9, 24.2. Anal. Calcd for: C_17_H_18_N_4_O_3_S: C, 56.97; H 5.06; N, 15.63; S, 8.95%. Found: C, 56.89; H, 5.01; N, 15.75; S, 9.07%. HRMS (*m*/*z*): [M – H]^−^ calcd, 357.1021; found, 357.1176.

#### 4-(1-(3-Nitrophenyl)-1*H*-1,2,3-triazol-4-yl)phenyl Sulfamate **4k**

Yield: 63%; mp: 225–226 °C (with decomposition); ν_max_ (KBr)/cm^–1^: 3342, 1530, 1484, 1352, 1181, 1161, 1054, 925, 869, 749, 666; ^1^H NMR δ_H_ (400 MHz, DMSO): 9.59 (1H, s, CH), 8.79 (1H, t, *J* = 2.1 Hz, Ar–H), 8.47 (1H, dd, *J* = 8.1, 2.1 Hz, Ar–H), 8.37 (1H, dd, *J* = 8.3, 2.2 Hz, Ar–H), 8.09 (2H, s, NH_2_), 8.04 (2H, d, *J* = 8.8 Hz, Ar–H), 7.95 (1H, t, *J* = 8.2 Hz, Ar–H), 7.44 (2H, d, *J* = 8.8 Hz, Ar–H); ^13^C NMR δ_C_ (101 MHz, DMSO): 150.6, 149.0, 147.3, 137.6, 132.1, 128.8, 127.2, 126.4, 123.7, 123.4, 120.7, 115.1. Anal. Calcd for: C_14_H_11_N_5_O_5_S: C, 46.54; H 3.07; N, 19.38; S, 8.87%. Found: C, 46.68; H, 3.01; N, 19.31; S, 8.98%. HRMS (*m*/*z*): [M – H]^−^ calcd, 360.0403; found, 360.0553.

#### 4-(1-(3,5-Dibromophenyl)-1*H*-1,2,3-triazol-4-yl)phenyl Sulfamate **4l**

Yield: 50%; mp: 228–229 °C (with decomposition); ν_max_ (KBr)/cm^–1^: 3334, 1578, 1497, 1373, 1178, 1155, 1053, 943, 872, 750, 663; ^1^H NMR δ_H_ (400 MHz, DMSO): 9.46 (1H, s, CH), 8.26 (2H, d, *J* = 1.6 Hz, Ar–H), 8.09 (2H, s, NH_2_), 8.02 (1H, t, *J* = 1.6 Hz, Ar–H), 7.99 (2H, d, *J* = 8.7 Hz, Ar–H), 7.44 (2H, d, *J* = 8.7 Hz, Ar–H); ^13^C NMR δ_C_ (101 MHz, DMSO): 150.6, 147.1, 138.8, 133.9, 128.7, 127.2, 123.9, 123.4, 122.2, 120.7. Anal. Calcd for: C_14_H_10_Br_2_N_4_O_3_S: C, 35.47; H 2.13; N, 11.82; S, 6.76%. Found: C, 35.57; H, 2.96; N, 11.73; S, 6.88%. HRMS (*m*/*z*): [M – H]^−^ calcd, 472.8742; found, 472.8868.

#### 4-(1-(3,5-Diiodophenyl)-1*H*-1,2,3-triazol-4-yl)phenyl Sulfamate **4m**

Yield: 60%; mp: 213–216 °C (with decomposition); ν_max_ (KBr)/cm^–1^: 3296, 1574, 1494, 1364, 1175, 1156, 1045, 955, 864, 761, 665; ^1^H NMR δ_H_ (400 MHz, DMSO): 9.43 (1H, s, CH), 8.37 (2H, d, *J* = 1.4 Hz, Ar–H), 8.24 (1H, t, *J* = 1.3 Hz, Ar–H), 8.08 (2H, s, NH_2_), 7.99 (2H, d, *J* = 8.7 Hz, Ar–H), 7.43 (2H, d, *J* = 8.8 Hz, Ar–H); ^13^C NMR δ_C_ (101 MHz, DMSO): 150.5, 147.1, 144.8, 138.4, 128.8, 127.9, 127.1, 123.4, 120.6, 97.4. Anal. Calcd for: C_14_H_10_I_2_N_4_O_3_S: C, 29.60; H 1.77; N, 9.86; S, 5.64%. Found: C, 29.74; H, 1.69; N, 9.91; S, 5.77%. HRMS (*m*/*z*): [M – H]^−^ calcd, 566.8485; found, 566.8643.

### Molecular Modeling

#### Ligands and Molecular Target Preparation

The 3D structures of the potential STS inhibitors (ligands) were prepared using Portable HyperChem 8.0.7 Release (Hypercube, Inc., Gainesville, FL, USA). Prior to docking calculations, the structure of each ligand was optimized using an MM + force field and the Polak–Ribière conjugate gradient algorithm (terminating at a gradient of 0.05 kcal mol^–1^ Å^–1^).

The X-ray structure of human STS was obtained from the Protein Data Bank (1P49). Prior to docking analysis, the structure of the protein was prepared using the protocol described below. Initially, the water molecules from crystallization were removed, and catalytic amino acid fGly75 was converted to the *gem*-diol form using the Maestro Protein Preparation Wizard module (Schrödinger, LLC, New York, NY, USA). Then, hydrogen atoms were introduced, and a prepared model of the protein was optimized using the OPLS-AA force field.

#### Molecular Docking

Docking calculations were carried out using AutoDock Vina 1.1.2 software (The Molecular Graphic Laboratory, The Scripps Research Institute, La Jolla, CA, USA).^35^ The grid box was centered on the Cβ atom of amino acid 75 of the prepared STS structure (the size of the grid box was 24 Å × 24 Å × 24 Å). After the docking procedure, the best poses for each individual ligand were inspected visually. The graphical 3D model was prepared using VMD 1.9 (University of Illinois at Urbana–Champaign, Urbana, IL, USA).

### Biological Assays

The inhibitory potency of the synthesized compounds was examined in two ways, including an enzymatic assay and the radioisotope cellular test. The enzymatic assay was performed using the STS enzyme isolated from human placenta and using radiolabeled [^3^H]E1S as a substrate. The radioisotope cellular assay was performed using the MCF-7 cell line in the presence of radiolabeled [^3^H]E1S.

#### In Vitro Enzymatic Assay

Evaluation of the inhibitory property of each compound was performed in the reaction mixture containing 20 mM Tris-HCl, pH 7.4, [^3^H]E1S (4 × 10^4^ Bq, 3 nM), 500 μM inhibitor, and 5 U of the purified enzyme (1 U is the amount of enzyme that hydrolyzes 100 μM NPS at 37 °C in 1 h).^36^ The total volume of the reaction mixture was 100 μL. The experiments were performed for 3 h at 37 °C. After incubation, the reaction mixture (90 μL) was collected from each well, and the product formed by STS hydrolysis was extracted with toluene (0.5 mL). STS activity was measured using a MicroBeta radioluminometer (PerkinElmer). Enzymatic assays were carried out in triplicate.

#### In Vitro Cellular Assay

The evaluation of the inhibitory effect of each compound with breast cancer cells was performed using a previously described method (27) with some modifications. MCF-7 cells were maintained in Dulbecco’s modified Eagle medium supplemented with 10% fetal bovine serum and cultured in the above medium until 80% confluence. For the measurement of STS inhibitory potency, cells were seeded in 24-well microplates (Nest Biotechnology) at a density of 1 × 10^5^ cells/well (the number of cells was determined using a Bürker Counting Chamber). Incubation of the cells was performed for 20 h at 37 °C in a 5% CO_2_ humidified incubator in a serum-free medium (0.5 mL) with the addition of [^3^H]E1S (4 × 10^4^ Bq, 3 nM) in the absence or presence of the inhibitor at an appropriate concentration: 100, 10, or 1 nM. After incubation, the medium (0.45 mL) was collected from each well, and the product formed by STS hydrolysis was extracted with toluene (4 mL). STS activity was measured using a MicroBeta radioluminometer (PerkinElmer). Assays with MCF-7 cells were carried out in triplicate.

#### Determination of STS Activity in Murine Livers and Tumors

Tumors and livers of mice treated with inhibitors (**4a**, **4b**, **5e**, **5g**, and **5l**) were homogenized with the CelLytic MT cell lysis reagent for mammalian tissues (Sigma) according to the manufacturer’s protocol. Briefly, the tissue samples were weighed, and then, the appropriate amount of the extraction buffer was added, maintaining the ratio of 20 mL of reagent per 1 g of tissue. Then, the samples were subjected to sonication in five cycles of 10 s each and centrifuged for 10 min at 14 000*g* to pellet the tissue debris. Total protein concentrations were determined in the obtained lysates using the Bradford method, and 100 μg of the total protein was used for each reaction as a source of STS activity. The reactions were performed for 3 h at 37 °C with the addition of [^3^H] E1S (4 × 10^4^ Bq, 3 nM) and 20 mM Tris-HCl, pH 7.4. The volume of the reaction mixtures was adjusted to 100 μL with water. After incubation, 60 μL of each reaction mixture was collected, and the product formed by STS hydrolysis was extracted with toluene (0.5 mL). STS activity was measured using a MicroBeta radioluminometer (PerkinElmer). Assays were carried out in triplicate.

### In Vivo Studies of Antitumor Activity

#### Cell Line

The mouse breast carcinoma 67NR cell line was obtained from Barbara Ann Karmanos Cancer Institute (Detroit, Michigan, USA) and is maintained at the Hirszfeld Institute of Immunology and Experimental Therapy (HIIET), PAS, Wroclaw, Poland. The cells were cultured in DMEM (Gibco, UK) with 10% calf bovine serum, iron-fortified (ATCC) and supplemented with 2 mM l-glutamine, 1% (v/v) minimum essential medium–non-essential amino acid solution 100×, 100 μg/mL streptomycin (all from Sigma-Aldrich), and 100 units/mL penicillin (from Polfa Tarchomin S.A., Poland). The cells were grown at 37 °C in a 5% CO_2_ humidified atmosphere.

#### Mice

Experiments were carried out on 7–8 weeks old female BALB/c mice with the approval of the Local Ethical Committee for Animal Experiments in Wroclaw (permission number: 77/2018) according to Directive 2010/63/EU of the European Parliament and Council on the protection of laboratory animals used for scientific purposes. Mice were purchased from the University of Bialystok (Poland). Animals were housed under the specific pathogen-free conditions of a 12 h day/night cycle with access to feed and water *ad libitum* at the Animal Facility HIIET PAS, Wroclaw, Poland. Experiments involving animals have been reported according to ARRIVE guidelines.^37^ All efforts were made to minimize animal suffering and to reduce the number of animals used.

#### Maximum Tolerated Dose

For the determination of the MTD, in the first step, Balb/c mice (female, three mice for each dose of the compound) received *per os* (PO) the tested compounds **4a**, **4b**, **5e**, **5g**, and **5l** at a dose of 10 mg/kg/day for 5 days a week for 2 weeks. The mice were weighed, and their general health was observed. In the next step, subsequent mice were administered with higher doses: 20 and 50 mg/kg. At the end of the MTD study, the autopsy was performed, and the internal organs (liver, kidney, and spleen) were weighed and macroscopically assessed.

#### Antitumor Activity

Mice were injected *orthotopically* (in the mammary gland fat pad) with 67NR mouse mammary tumor cells derived from in vitro culture (1.5 × 10^5^ cells/0.05 mL Hanks fluid/mouse). The growth of tumors has been observed. When the average volume of tumors was about 50 mm^3^, the mice were randomized into six groups with nine mice/group, and the *per os* administration of the tested compounds at the dose of 50 mg/kg/b.w. was started (5 days a week). Animals were observed during the next 17 days and euthanized. During observation, body weight and tumor growth were monitored three times a week. The volume of the tumors was calculated according to the following formula: TV = *a*^2^·*b*/2 [mm^3^], (where: *a*—width and *b*—length of the tumor). Blood, tumor tissue, and liver were harvested during autopsy for further analyses. Blood aliquots (about 500 μL) were collected in EDTA containing vials for morphology analysis (Mythic 18 analyzer, Orphee), and then, the plasma was collected (centrifuged at 2500*g* for 15 min at 4 °C within 1 h after collection) for biochemical parameter analysis (Cobas c111, Roche). Tumors and livers were kept frozen at −80 °C until further processing. The internal organs (liver, kidney, spleen, and uterus) were weighed and macroscopically assessed.

#### Determination of the Estradiol Level in Plasma

In the plasma of mice with 67NR tumors, the level of estradiol was determined by using an enzyme-linked immunosorbent assay (estradiol ELISA, Demedic) according to the manufacturer’s protocol. Absorbance (at 450 nm) was recorded using a BioTek Synergy H4 (Biokom, Poland).

#### Statistical Analysis

Statistical analysis was performed using STATISTICA version 10.1 (StatSoft Inc., USA). Mann–Whitney *U* test or one-way ANOVA was performed using GraphPad Prism 7, with *p* values below 0.05 considered as significant.

## Supporting Information Available

SMILES representation for compounds **4a**–**4m** and **5a**–**5m** (CSV)
Body weight and BWC after administration of tested STS inhibitors at doses of 10, 20, and 50 mg/kg; mice organ weight after administration of compounds at doses of 10, 20, and 50 mg/kg body weight; blood morphology of mice receiving tested compounds at the dose of 50 mg/kg; antitumor activity of STS inhibitors at the dose of 50 mg/kg; blood morphology and blood biochemistry of mice receiving tested compounds at the dose of 50 mg/kg; weight of internal organs of mice with 67NR tumor treated per os with tested compounds at the dose of 50 mg/kg body weight; and ^1^H NMR, ^13^C NMR, IR, HRMS, and HPLC trace data for compounds **4a**–**4m**, **5e**, **5g**, and **5l** (PDF)
Predicted binding mode of compound **4m** in the STS active site (PDB)
Predicted binding mode of compound **5l** in the STS active site (PDB)

## Supplementary Material

jm1c02220_si_002.pdf

jm1c02220_si_003.pdb

jm1c02220_si_004.pdb

## Notes

The atomic coordinates of the docking complexes are included in the Supporting Information.

## Abbreviations

ACN: acetonitrile
STS: steroid sulfatase
E1S: estrone-3-sulfate
DHEAS: dehydroepiandrosterone-3-sulfate
*t*-BuONO: *tert*-butyl nitrite
TMSN3: azidotrimethylsilane
TV: tumor volume
TGI: tumor growth inhibition
BWC: body weight change
ALT: alanine aminotransferase
AST: aspartate aminotransferase
WBC: white blood cell
RBC: red blood cell
HGB: hemoglobin
HCT: hematocrit
PLT: platelets
AcOEt: ethyl acetate
