# Supplementary material for: Development of Sulfamoylated 4-(1-Phenyl-1H-1,2,3-triazol-4-yl)phenol Derivatives as Potent Steroid Sulfatase Inhibitors for Efficient Treatment of Breast Cancer
Source: J Med Chem. 2022 Mar 2;65(6):5044–56. doi: 10.1021/acs.jmedchem.1c02220 (PMC8958511; doi:10.1021/acs.jmedchem.1c02220)

Supporting Information

Development of sulfamoylated 4-(1-phenyl-1*H*-1,2,3-triazol-4-yl)phenol derivatives as potent steroid sulfatase inhibitors for efficient treatment of breast cancer

Karol Biernacki [1], Olga Ciupak [1], Mateusz Daśko [2], Janusz Rachon [1], Witold Kozak [3], Janusz Rak [3], Konrad Kubiński [4], Maciej Masłyk [4], Aleksandra Martyna [4], Magdalena Śliwka-Kaszyńska [1], Joanna Wietrzyk [5], Marta Świtalska [5], Alessio Nocentini [6*], Claudiu T. Supuran [6] and Sebastian Demkowicz [1*]

[1] Department of Organic Chemistry, Faculty of Chemistry, Gdańsk University of Technology, Narutowicza 11/12, 80-233 Gdansk, Poland,

[2] Department of Inorganic Chemistry, Faculty of Chemistry, Gdańsk University of Technology, Narutowicza 11/12, 80-233 Gdansk, Poland,

[3] Department of Physical Chemistry, Faculty of Chemistry, University of Gdańsk, Wita Stwosza 63, 80-308 Gdansk, Poland,

[4] Department of Molecular Biology, Faculty of Biotechnology and Environment Sciences, The John Paul II Catholic University of Lublin, Konstantynów 1i, 20-708 Lublin, Poland

[5] Department of Experimental Oncology, Hirszfeld Institute of Immunology and Experimental Therapy, Rudolfa Weigla 12, 53-114 Wrocław, Poland

[6] Department of NEUROFARBA, Pharmaceutical and Nutraceutical Section, University of Florence, Via U. Schiff 6, 50019, Sesto Fiorentino, Firenze, Italy

* Correspondence: alessio.nocentini@unifi.it (A.N.); sebastian.demkowicz@pg.edu.pl (S.D.)

Table of contents

Table of contents	P
**Figure S1**. Body weight (a) and changes in body weight (b) after administration of tested STS inhibitors at the dose of 10 mg/kg.	**S2**
**Figure S2**. Body weight (a) and changes in body weight (b) after administration of tested STS inhibitors at the dose of 20 mg/kg.	**S2**
**Figure S3**. Body weight (a) and changes in body weight (b) after administration of tested STS inhibitors at the dose of 50 mg/kg.	**S2**
**Figure S4**. Mice organ weight after compounds administration at the doses of 10, 20 or 50 mg/kg b.w. – MTD study.	**S3**
**Table S1**. Blood morphology of mice receiving tested compounds at a dose of 50 mg/kg – MTD study.	**S3**
**Figure S5**. Antitumor activity of STS inhibitors at a dose of 50 mg / kg: individual plots for groups	**S4**
**Table S2.** Blood morphology of mice receiving tested compounds at a dose of 50 mg/kg	**S4**
**Table S3.** Blood biochemistry of mice receiving tested compounds at a dose of 50 mg/kg.	**S5**
**Figure S6.** Blood biochemistry of mice receiving tested compounds at a dose of 50 mg/kg. Hepatic (a) and renal (b) parameters.	**S5**
**Figure S7**. Weight of internal organs of mice with 67NR tumor treated per os with tested compounds at the dose 50 mg/kg b.w.	**S6**
^{1}H NMR, ^{13}C NMR, IR, HRMS, HPLC trace for compounds **4a-m**, **5e**, **5g**, and **5l**	**S7-S48**

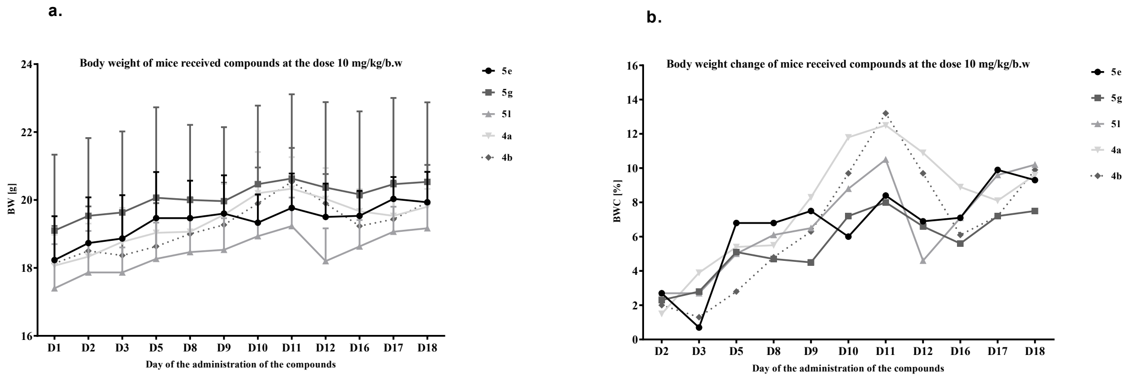

Figure S1. Body weight (a) and changes in body weight (b) after administration of tested STS inhibitors at the dose of 10 mg/kg.

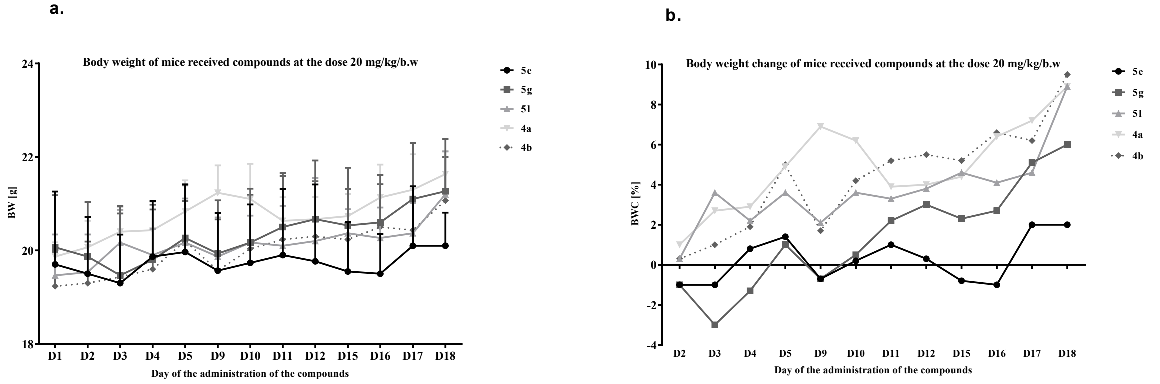

Figure S2. Body weight (a) and changes in body weight (b) after administration of tested STS inhibitors at the dose of 20 mg/kg.

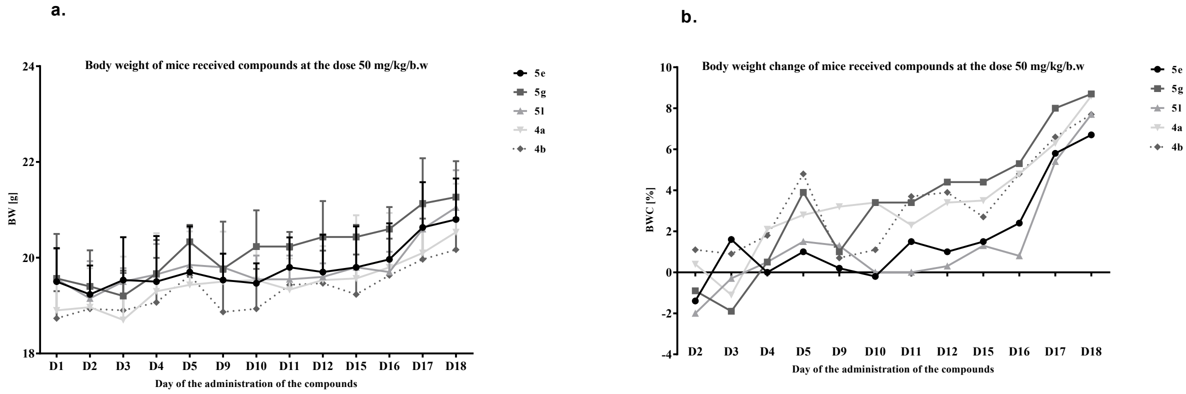

Figure S3. Body weight (a) and changes in body weight (b) after administration of tested STS inhibitors at the dose of 50 mg/kg.

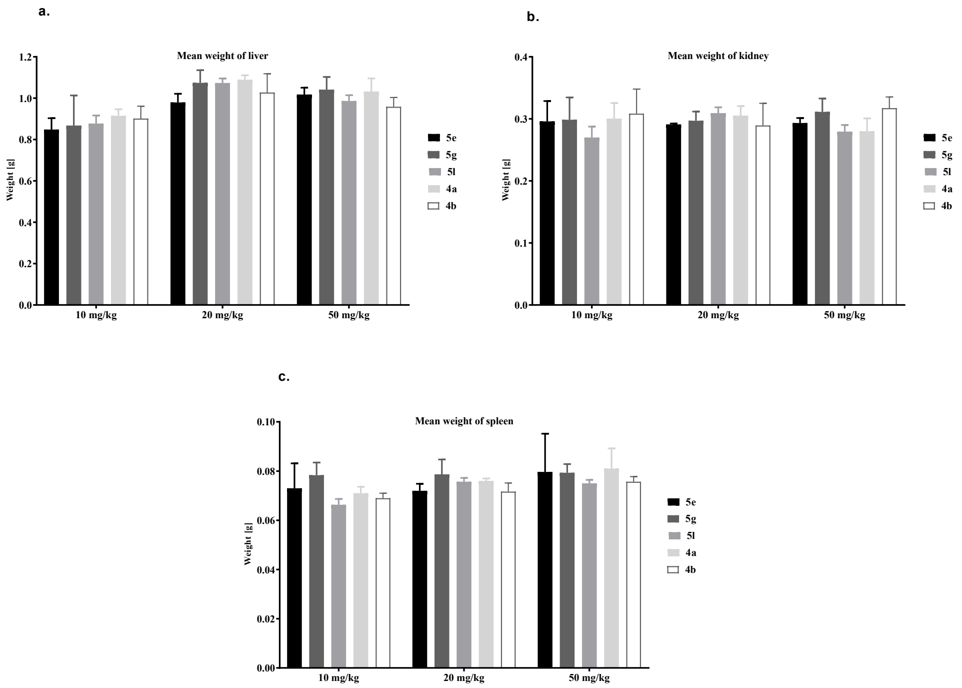

Figure S4. Mice organ weight after compounds administration at the doses of 10, 20 or 50 mg/kg b.w. – MTD study.

Table S1. Blood morphology of mice receiving tested compounds at a dose of 50 mg/kg.

Group	WBC	LIMF	MON	GRAN
	10x3/uL	*%*	*%*	*%*
Healthy mice	*5,9±1,5*	*80,3±5,4*	*4,4±1*	*15,3±6*
**5e**	4,0±0,4	85,6±2,6	3,3±1	11,1±1,9
**5g**	4,9±1,3	84,3±2,3	3,4±0,6	12,4±1,7
**5l**	4,2±0,6	86,6±0,2	2,9±0,4	10,6±0,2
**4a**	4,5±0,3	83,5±4,1	3,3±0,6	13,2±3,6
**4b**	5,1±1,6	85,7±1,7	3,3±0,5	11,0±1,7

Group	RBC	HGB	HCT	MCV	MCH	MCHC	RDW	PLT
	10x6/uL	*g/dL*	*%*	*fL*	*pg*	*g/dL*	*%*	*10x3/uL*
Healthy mice	*8,5±0,6*	*14,1±1,1*	*39,2±1,6*	*48,3±0,9*	*17±0,3*	*35,3±0,5*	*16,8±1*	*482±27*
**5e**	9,2±0,4	15,7±0,5	42,3±1,3	46±0,5	17,1±0,2	37,2±0,7	17,6±0,7	511±30
**5g**	8,8±0,01	15,2±0,2	41,1±0,5	46,9±0,3	17,2±0,2	36,7±0,2	17,4±0,3	522±33
**5l**	8,9±0,4	15,4±0,5	42±1,9	47,2±0,2	17,3±0,1	36,6±0,4	17,3±0,2	596±64
**4a**	8,8±0,1	15,1±0,2	41±0,2	46,8±0,2	17,3±0,3	36,9±0,5	17,9±0,6	518±49
**4b**	9,4±0,4	16,4±0,6	44,1±1,3	46,9±0,5	17,4±0,01	37,1±0,4	17,5±0,6	533±34

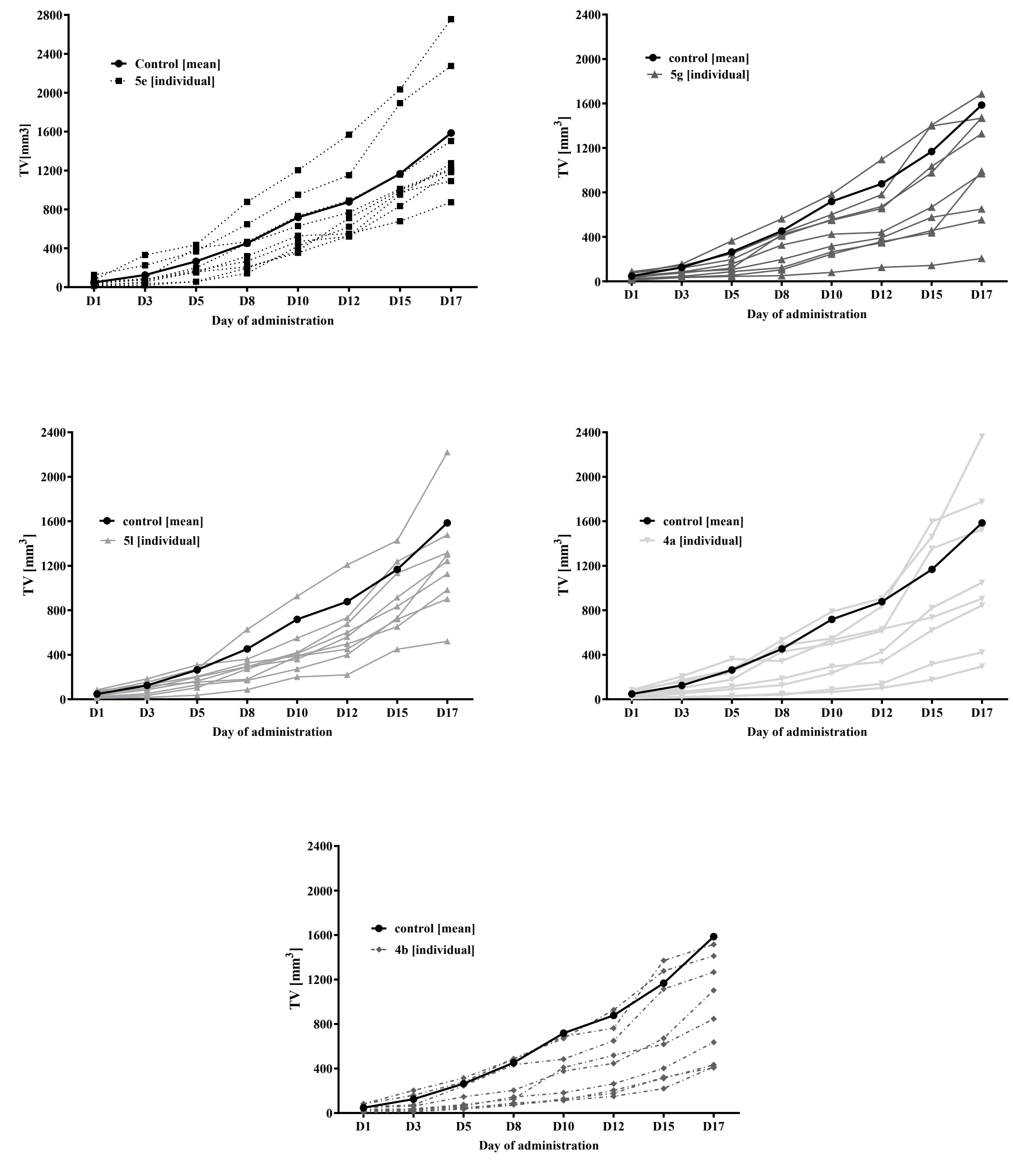

Figure S5. Antitumor activity of STS inhibitors at a dose of 50 mg/kg: individual plots for groups

Table S2. Blood morphology of mice receiving tested compounds at a dose of 50 mg/kg

Group	WBC	LIMF	MON	GRAN	LIMF	MON	GRAN
	x10³/uL	*x10³/uL*	*x10³/uL*	*x10³/uL*	%	%	%
Healthy mice	*5,9±1,5*	*4,7±1*	*0,2±0,1*	*0,9±0,6*	*80,3±5,4*	*4,4±1*	*15,3±6*
**Control**	7,7±3,5	5,2±2,7	0,7±0,2	1,9±0,7	66±5,2	9,3±2	24,7±3,8
**5e**	6,1±2,1	4,4±1,8	0,5±0,1	1,3±0,4*	70,7±6,3	8,5±2,1	20,8±5,1
**5g**	5,2±1,8	3,7±1,2	0,5±0,2	1,1±0,6*	71,5±8	9±2,5	19,6±5,9
**5l**	4,8±1,1*	3,3±0,7	0,5±0,1*	1,1±0,5*	67,6±7,4	9,7±2,2	22,7±7
**4a**	5,2±1	3,7±0,7	0,5±0,1	1±0,4*	72,1±7,6	9,2±2,1	18,7±5,8*
**4b**	5,2±1,4	3,7±1,3	0,5±0,1*	1,1±0,5*	70,3±9,3	8,6±1,4	21,1±8,2

Group	RBC	HGB	HCT	MCV	MCH	MCHC	RDW
	x10⁶/uL	*g/dL*	*%*	*fL*	*pg*	*g/dL*	*%*
Healthy mice	*8,5±0,6*	*14,1±1,1*	*39,2±1,6*	*48,3±0,9*	*17±0,3*	*35,3±0,5*	*16,8±1*
**Control**	7,7±0,5	13,9±0,6	35,6±1,8	46,4±0,8	18,2±0,4	39,2±0,6	18,9±0,6
**5e**	7,4±0,4	13,4±0,6	34,6±1,6	47±1,2	18,2±0,4	38,6±0,5	19,2±1,2
**5g**	7,4±0,7	13,5±0,9	35±2,4	47,4±2,8	18,3±0,9	38,6±0,6	19,1±1,5
**5l**	7,5±0,5	13,3±0,8	34,7±2,1	46,4±0,7	17,8±0,3	38,3±0,4*	18,4±1
**4a**	7,6±0,8	13,5±1,1	35,9±3,2	47,1±1	17,7±0,5*	37,6±0,7*	18±0,5*
**4b**	7,8±0,7	13,7±0,9	36,6±2,7	46,8±0,9	17,6±0,5*	37,6±0,6*	18,2±0,5*

Group	PLT	MPV	PDW	PCT
	x10³/uL	*fL*	*fL*	*%*
Healthy mice	*482±27*	*5,7±0,1*	*26±2,4*	*0,276±0,02*
**Control**	516,1±169,5	5±0,3	31,4±8,7	0,254±0,08
**5e**	573,1±95,6	4,9±0,1	25,6±3,8	0,280±0,04
**5g**	508,6±69	5±0,1	26,9±4,8	0,255±0,04
**5l**	463,1±74	5±0,1	29,3±4,6	0,233±0,035
**4a**	503,3±59,8	4,9±0,1	24,9±3	0,250±0,03
**4b**	512,2±72,3	4,9±0,2	27,1±3,2	0,248±0,03

**p<0.05, statistical significance*

Table S3. Blood biochemistry of mice receiving tested compounds at a dose of 50 mg/kg.

Group	ALT	AST	creatinine	urea
	U/L	*U/L*	*umol/L*	*mmol/L*
Healthy mice	*27,2±12,7*	*106,9±42,2*	*8,3±0,91*	*6,1±2,08*
**Control**	25,4±5,2	96,3±22,7	5,87±1,53	6,26±0,89
**5e**	31,1±10,7	97,4±24,9	5,33±1,21	7,65±0,54*
**5g**	30,3±4,3*	102,2±23,1	6,51±1,49	8,83±1,32*
**5l**	31,5±5,7*	107,2±21,4	5,6±1,65	8,1±0,7*
**4a**	27,3±3,9	91,7±15,4	5,75±1,52	7,92±0,66*
**4b**	27,1±3,8	86,6±18,5	4,53±1,48	6,94±0,69

**p<0.05, statistical significance*

Figure S6. Blood biochemistry of mice receiving tested compounds at a dose of 50 mg/kg. Hepatic (a) and renal (b) parameters.

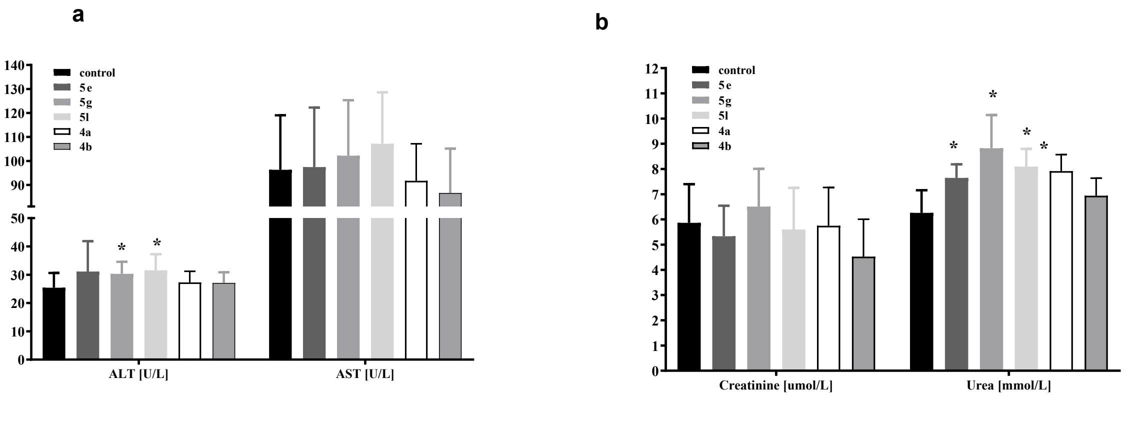

Level of biochemistry parameters in plasma of mice with 67NR tumor treated *per os* with tested compounds at the dose 50 mg/kg b.w. a) level of alanine aminotransferase (ALT) and aspartate aminotransferase (AST), b) creatinine and urea concentration; N=9; statistical analysis: Mann-Whitney U test, *p<0.05 vs control group

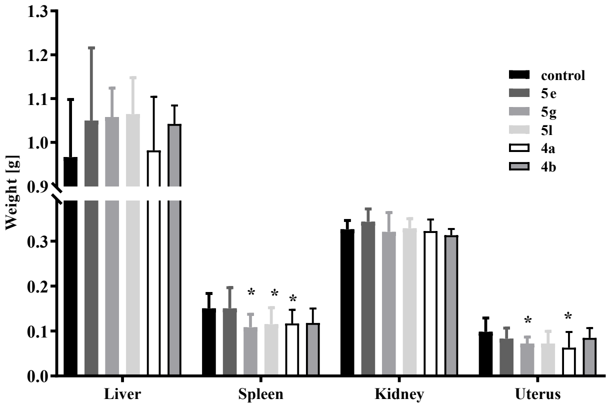

Figure S7. Weight of internal organs of mice with 67NR tumor treated *per os* with tested compounds at the dose 50 mg/kg b.w. N=9; statistical analysis: Mann-Whitney U test, *p<0.05 vs control group

4a

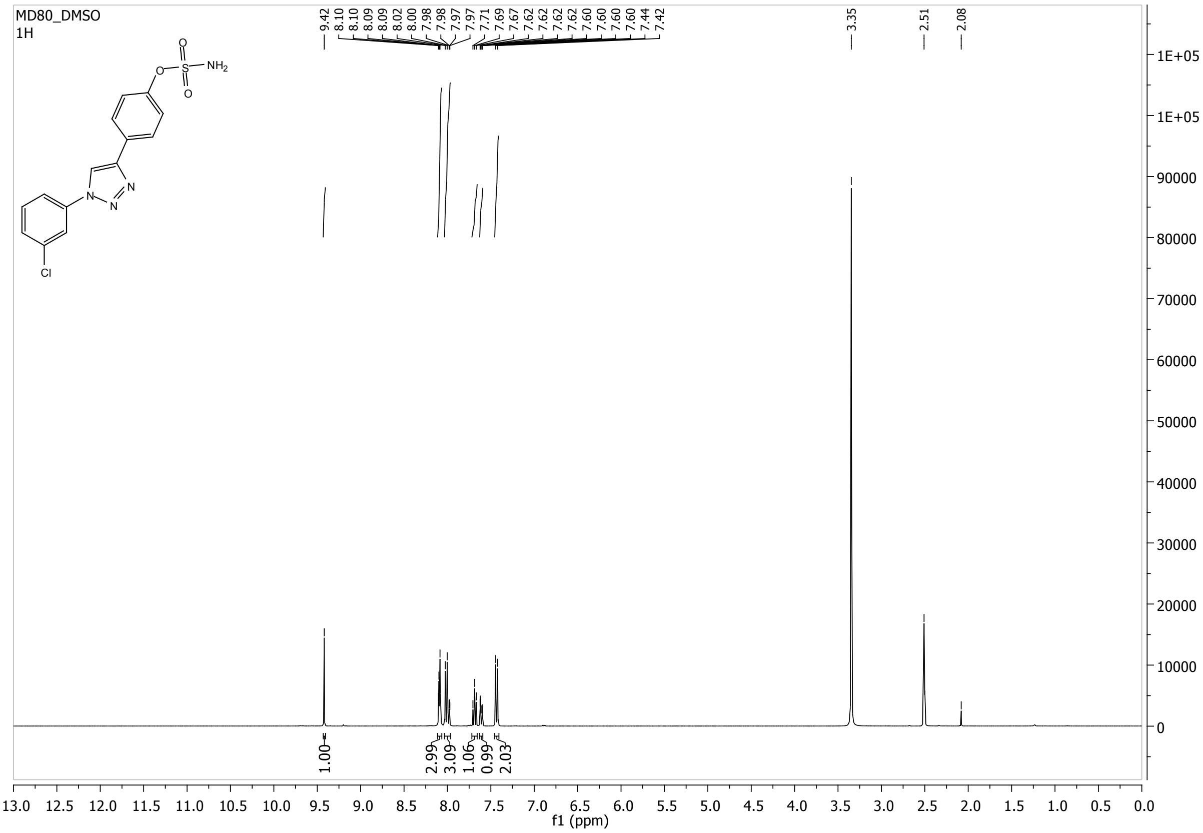
MD80_DMSO
1H
9.42
8.10
8.10
8.09
8.09
8.02
8.00
7.98
7.98
7.97
7.97
7.71
7.69
7.67
7.62
7.62
7.62
7.62
7.60
7.60
7.60
7.60
7.44
7.42
3.35
2.51
2.08
NH2
O
S
N
Cl
1.00
2.99
3.09
1.06
0.99
2.03
f1 (ppm)

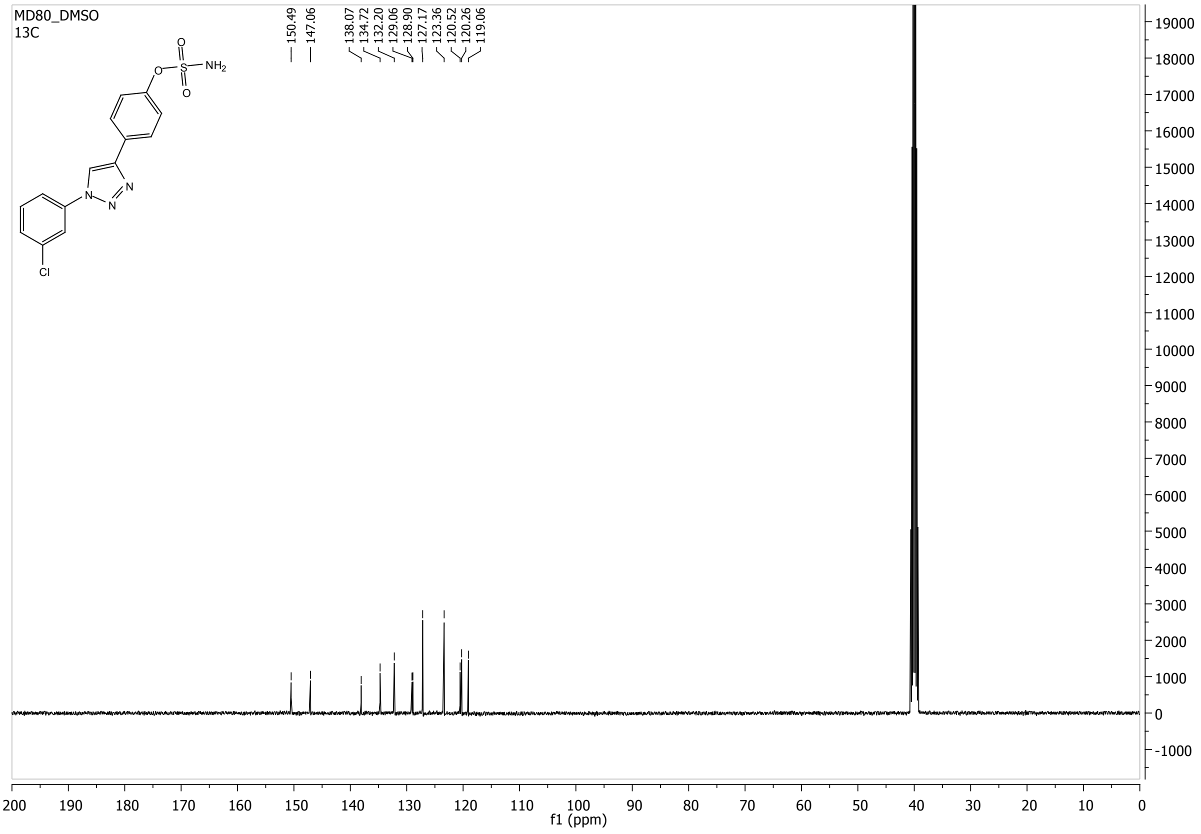
MD80_DMSO
13C
150.49
147.06
138.07
134.72
132.20
129.06
128.90
127.17
123.36
120.52
120.26
119.06
NH2
O
S
N
Cl
f1 (ppm)

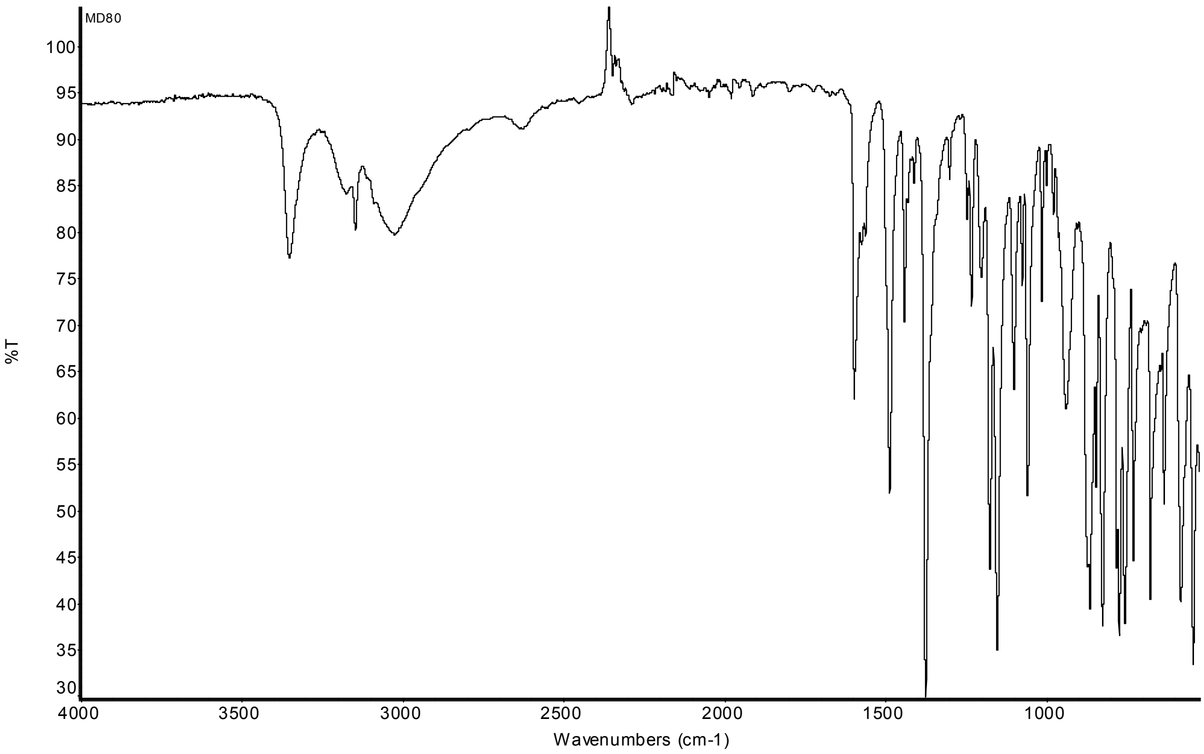

Acq. Time: 09:42
Acq. Date: Saturday, April 07, 2018

Batch Name: ManualTune.bat
Acq. File: MT20180407094219.wiff

Scan Mode: Zero Width
Polarity/Scan Type: Negative

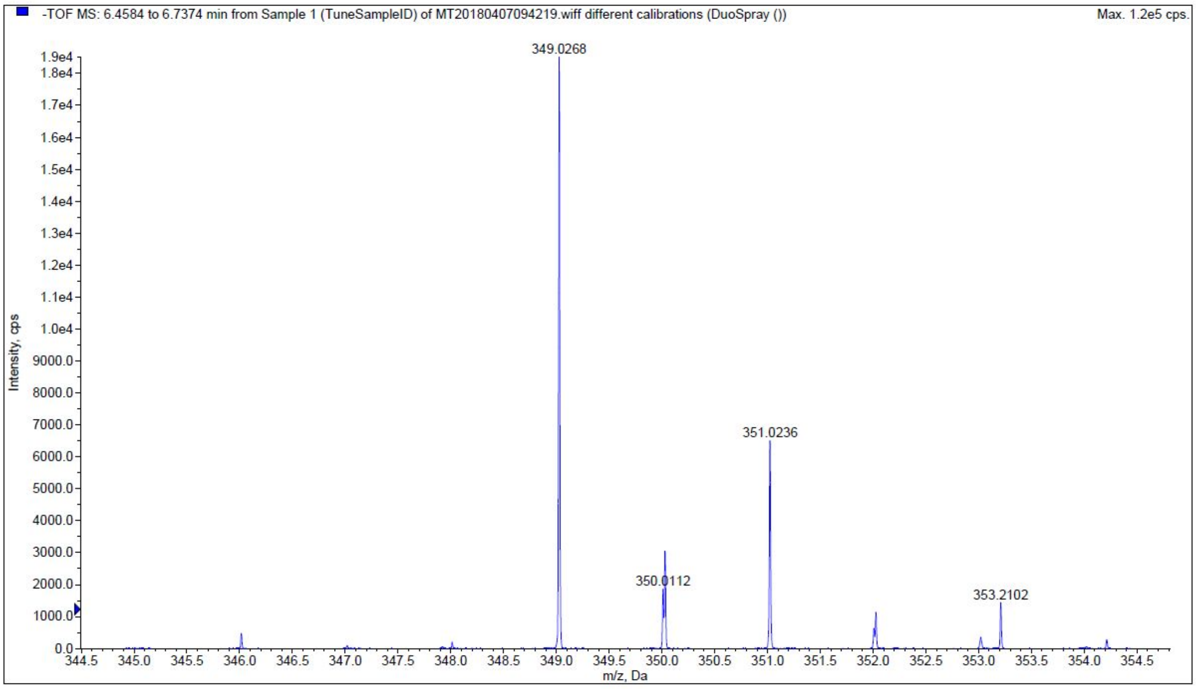

Printing Time: 9:53:27 AM
Printing Date: Saturday, April 07, 2018

Workstation: TRIPLETOF5600
Operator: Uniwersytet Gdanski

Analyst Version: 1.7.1
Page 1 of 1

HPLC purity 99.619 %

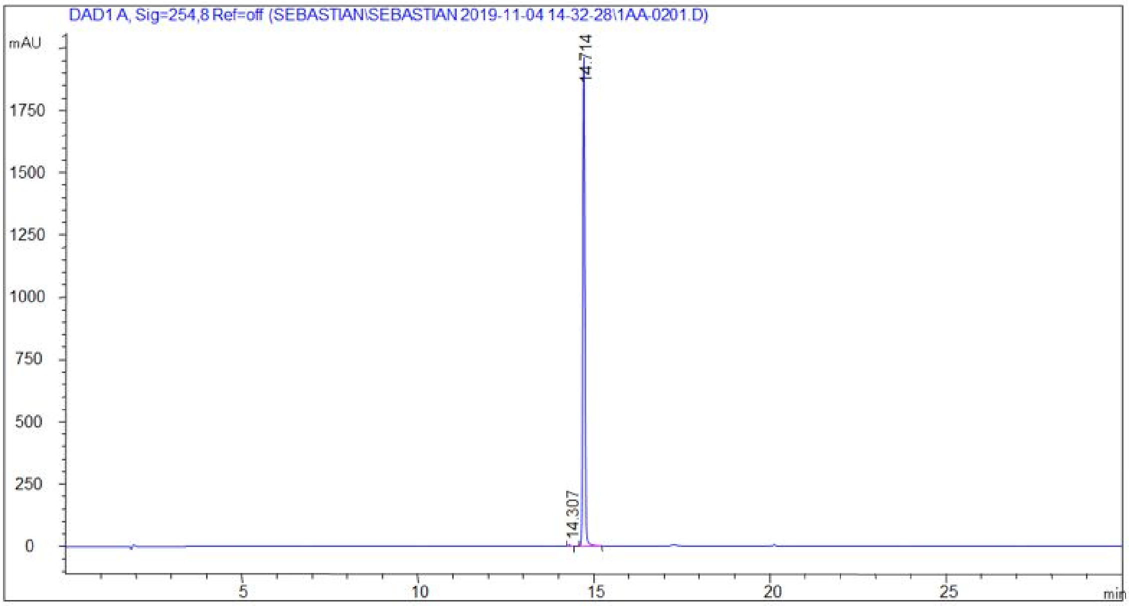

4b

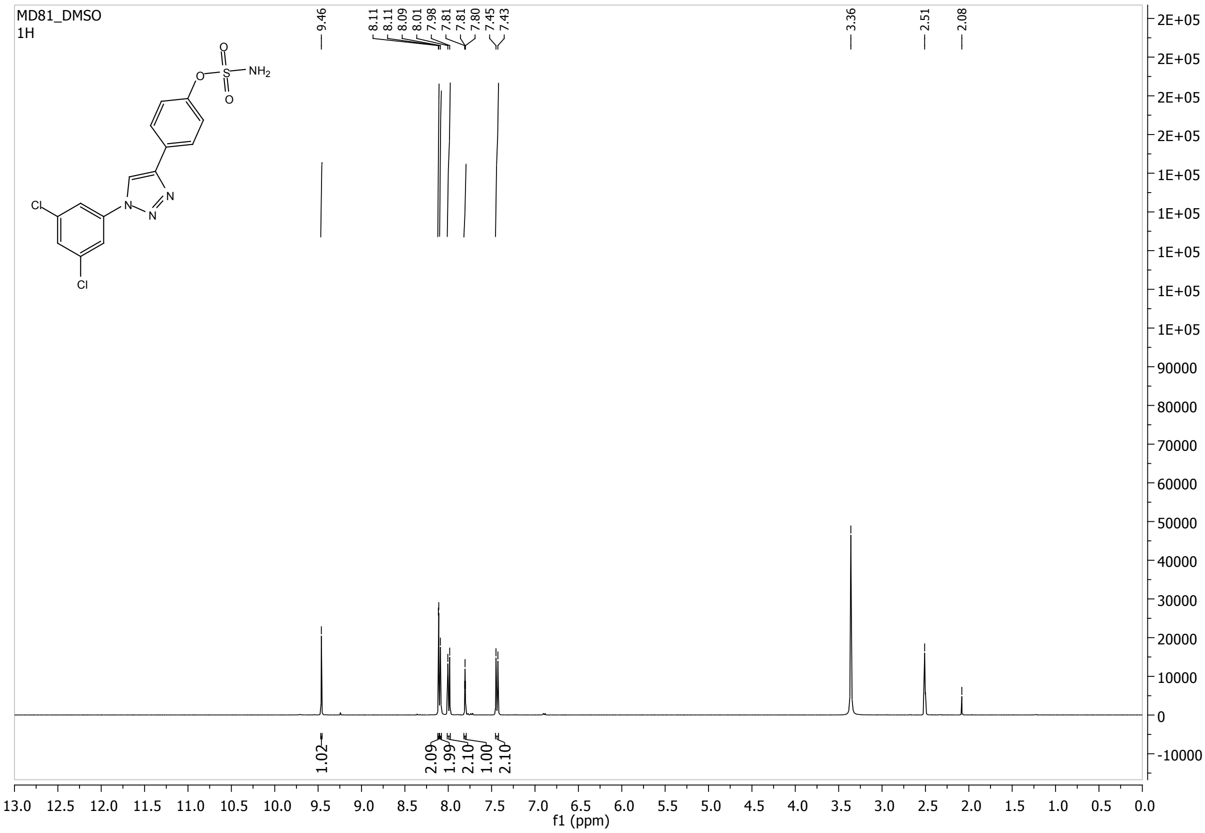
MD81_DMSO
1H
9.46
8.11
8.11
8.09
8.01
7.98
7.81
7.81
7.80
7.45
7.43
3.36
2.51
2.08
1.02
2.09
1.99
2.10
1.00
2.10
f1 (ppm)

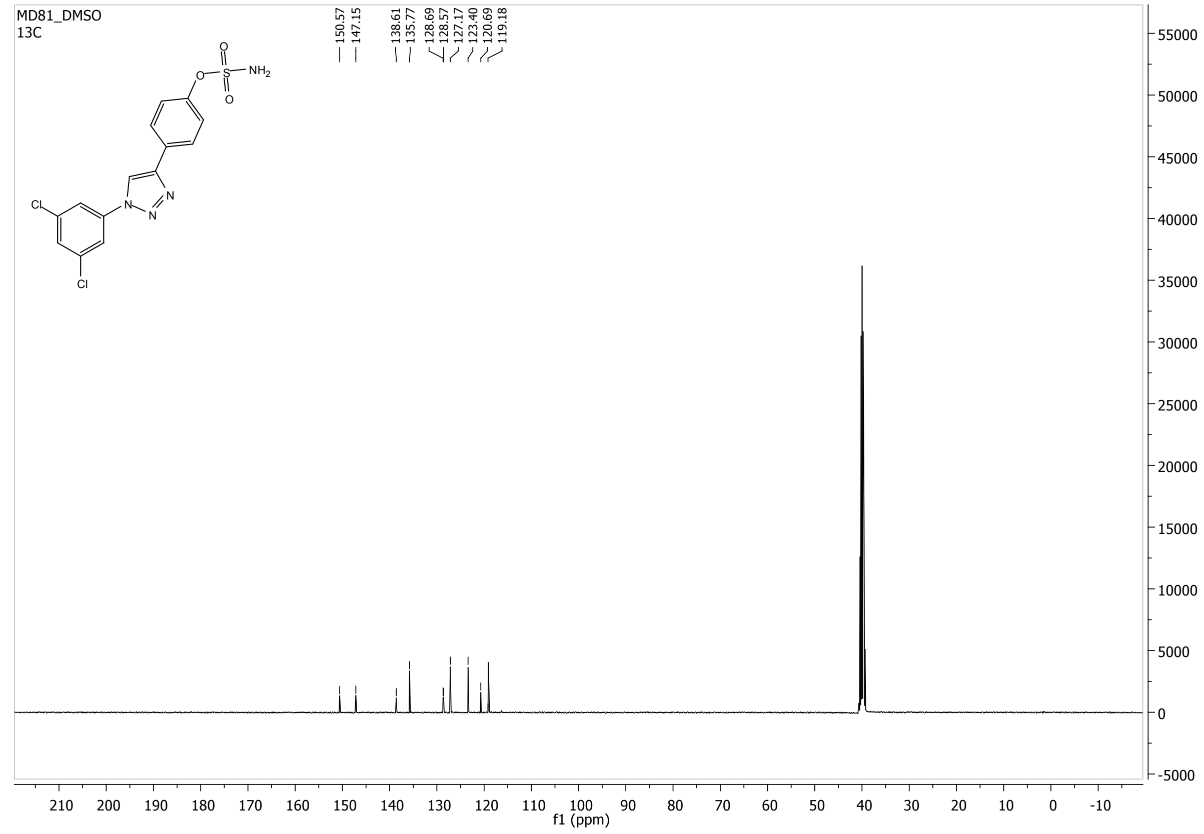
MD81_DMSO
13C
150.57
147.15
138.61
135.77
128.69
128.57
127.17
123.40
120.69
119.18
f1 (ppm)

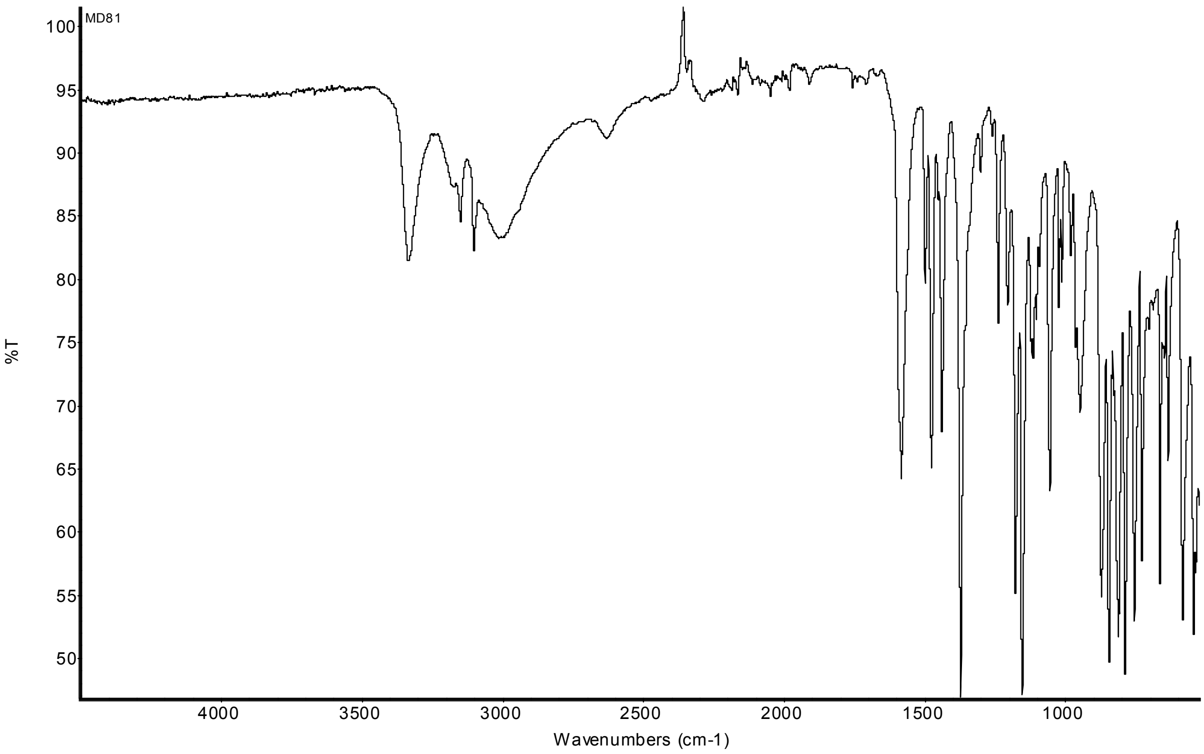

Acq. Time: 09:42
Acq. Date: Saturday, April 07, 2018

Batch Name: ManualTune.bat
Acq. File: MT20180407094219.wiff

Scan Mode: Zero Width
Polarity/Scan Type: Negative

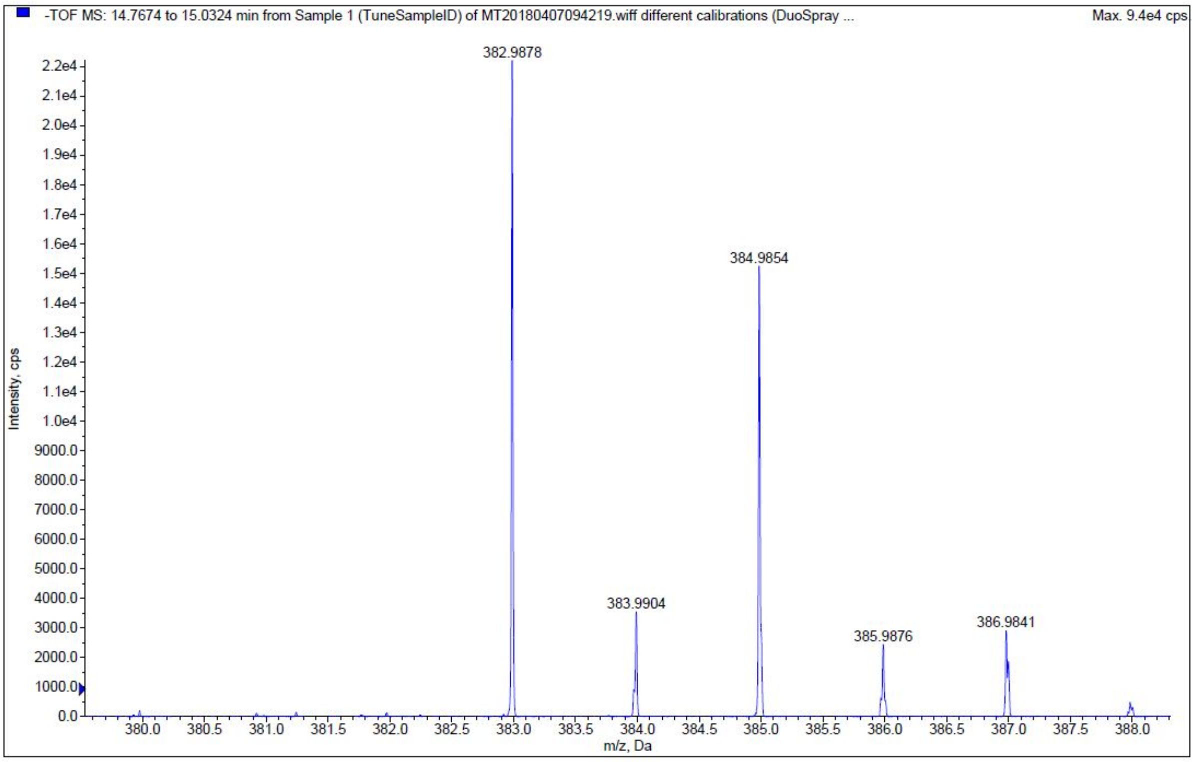

Printing Time: 9:57:42 AM
Printing Date: Saturday, April 07, 2018

Workstation: TRIPLETOF5600
Operator: Uniwersytet Gdanski

Analyst Version: 1.7.1
Page 1 of 1

HPLC purity 96.778 %

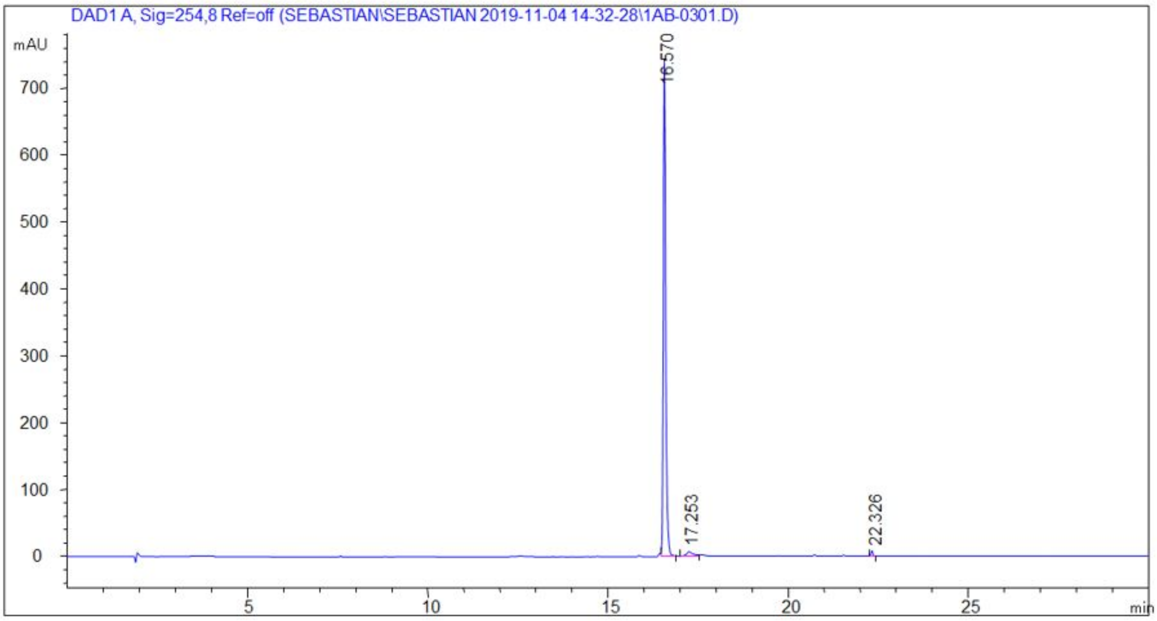

4c

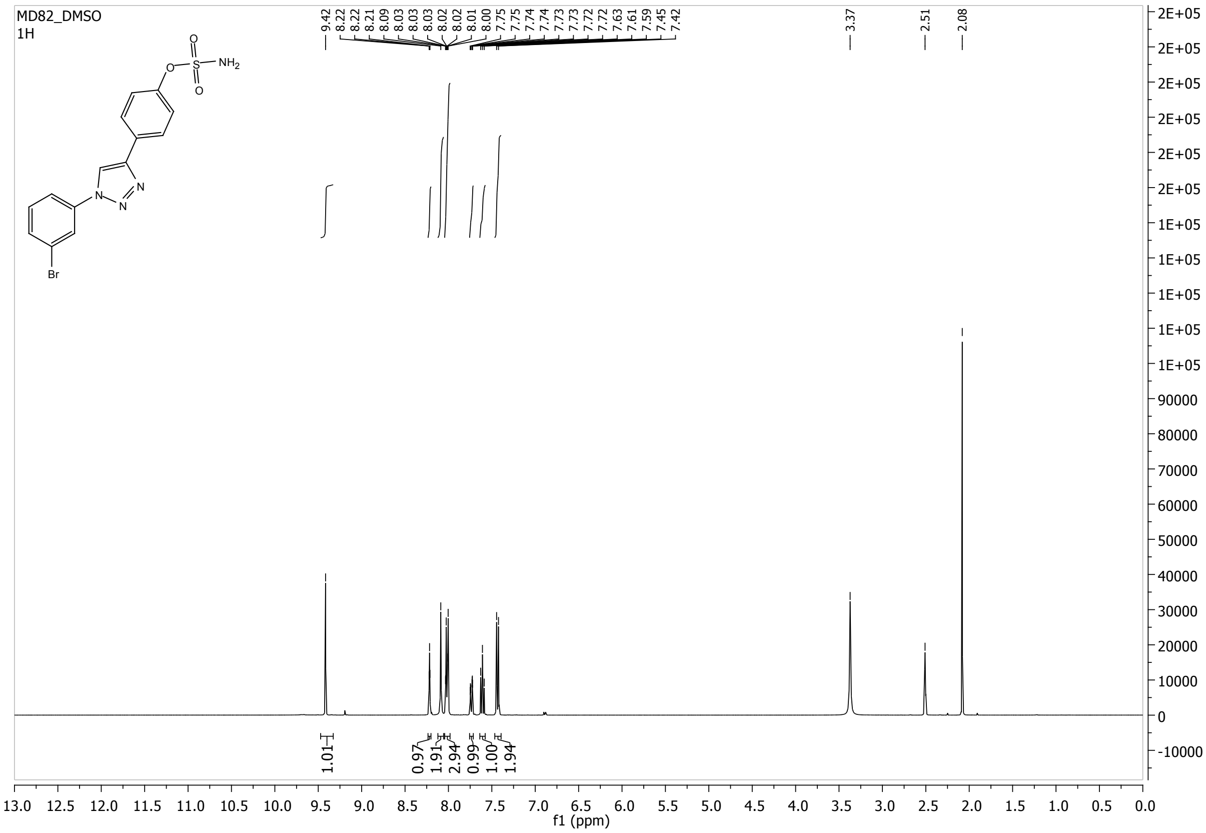
MD82_DMSO
1H
9.42
8.22
8.22
8.21
8.09
8.03
8.03
8.03
8.02
8.02
8.01
8.00
7.75
7.75
7.74
7.74
7.73
7.73
7.72
7.72
7.63
7.61
7.59
7.45
7.42
3.37
2.51
2.08
1.01
0.97
1.91
2.94
0.99
1.00
1.94
NH2
Br
f1 (ppm)

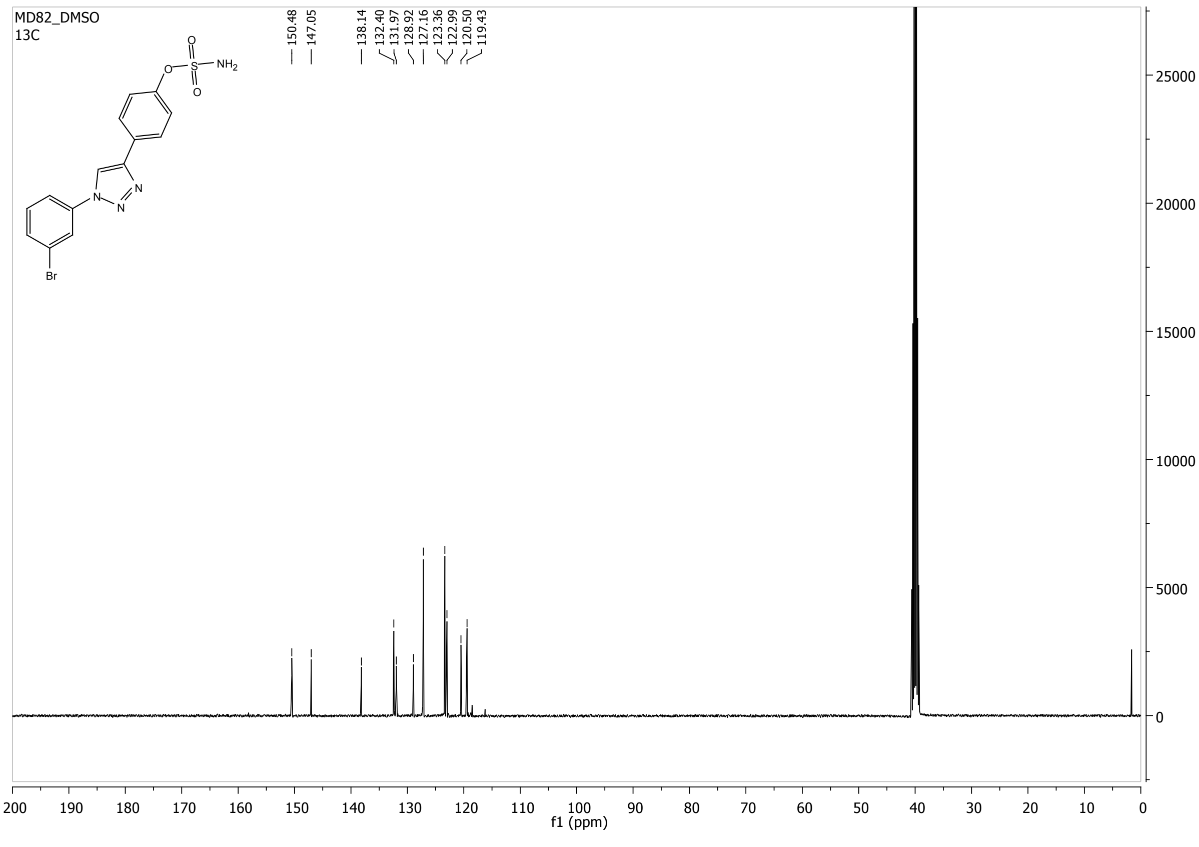
MD82_DMSO
13C
150.48
147.05
138.14
132.40
131.97
128.92
127.16
123.36
122.99
120.50
119.43
NH2
Br
f1 (ppm)

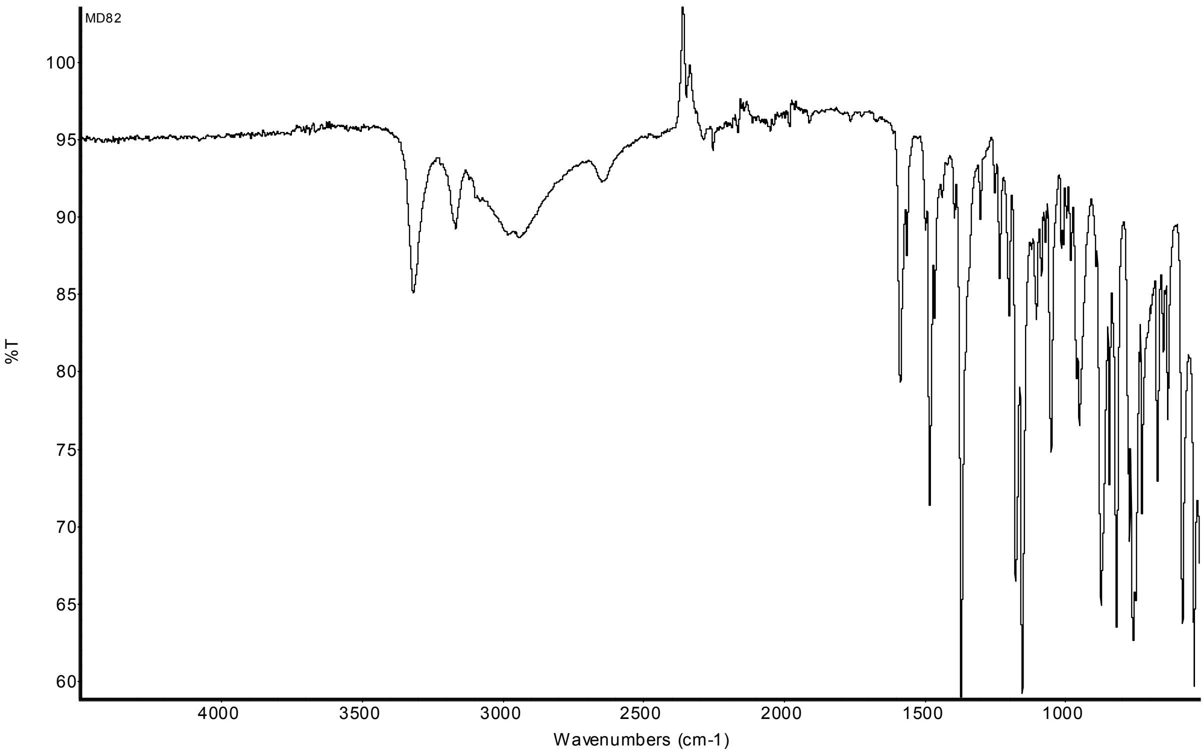

Acq. Time: 09:42
Acq. Date: Saturday, April 07, 2018

Batch Name: ManualTune.bat
Acq. File: MT20180407094219.wiff

Scan Mode: Zero Width
Polarity/Scan Type: Negative

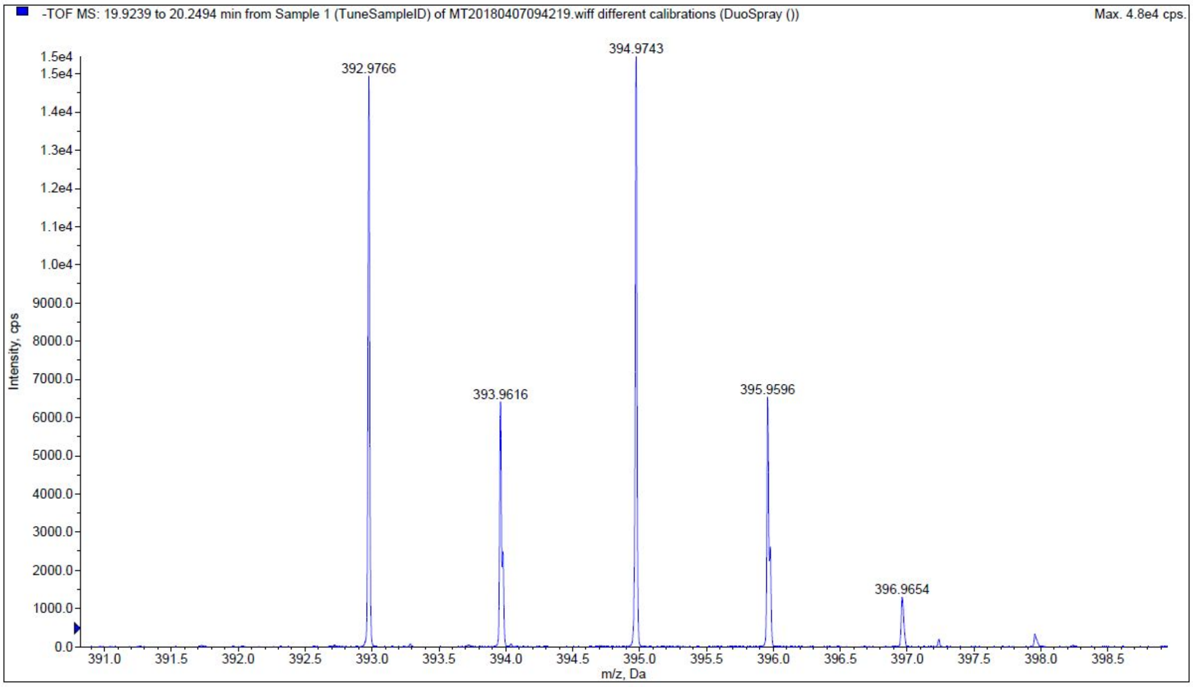

Printing Time: 10:03:40 AM
Printing Date: Saturday, April 07, 2018

Workstation: TRIPLETOF5600
Operator: Uniwersytet Gdanski

Analyst Version: 1.7.1
Page 1 of 1

HPLC purity 98.203 %

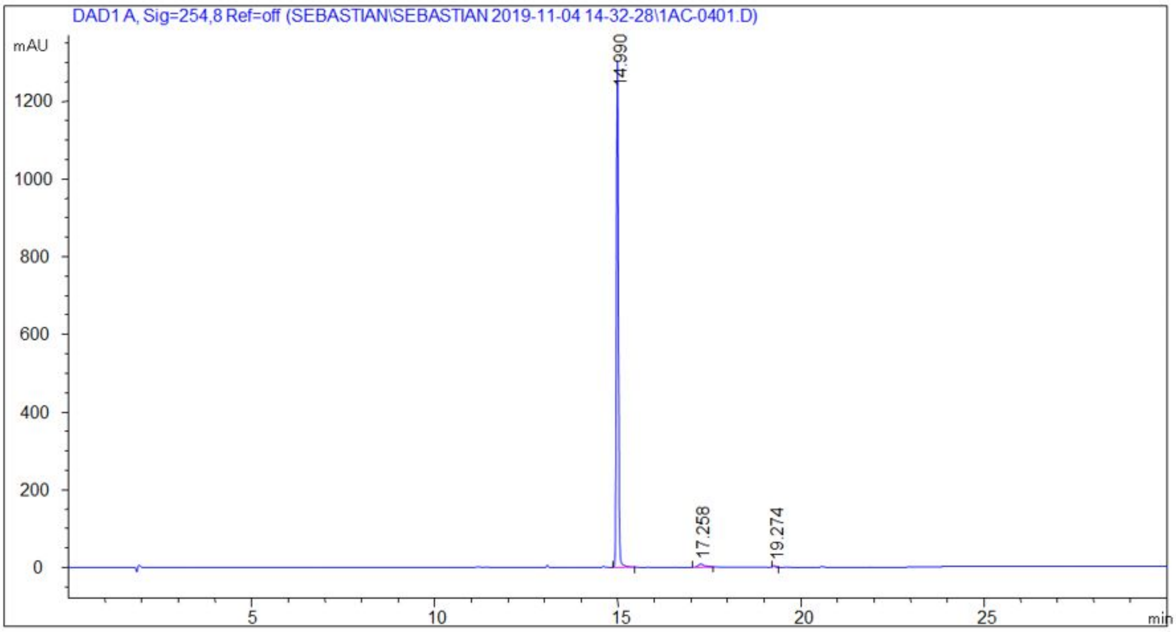

4d

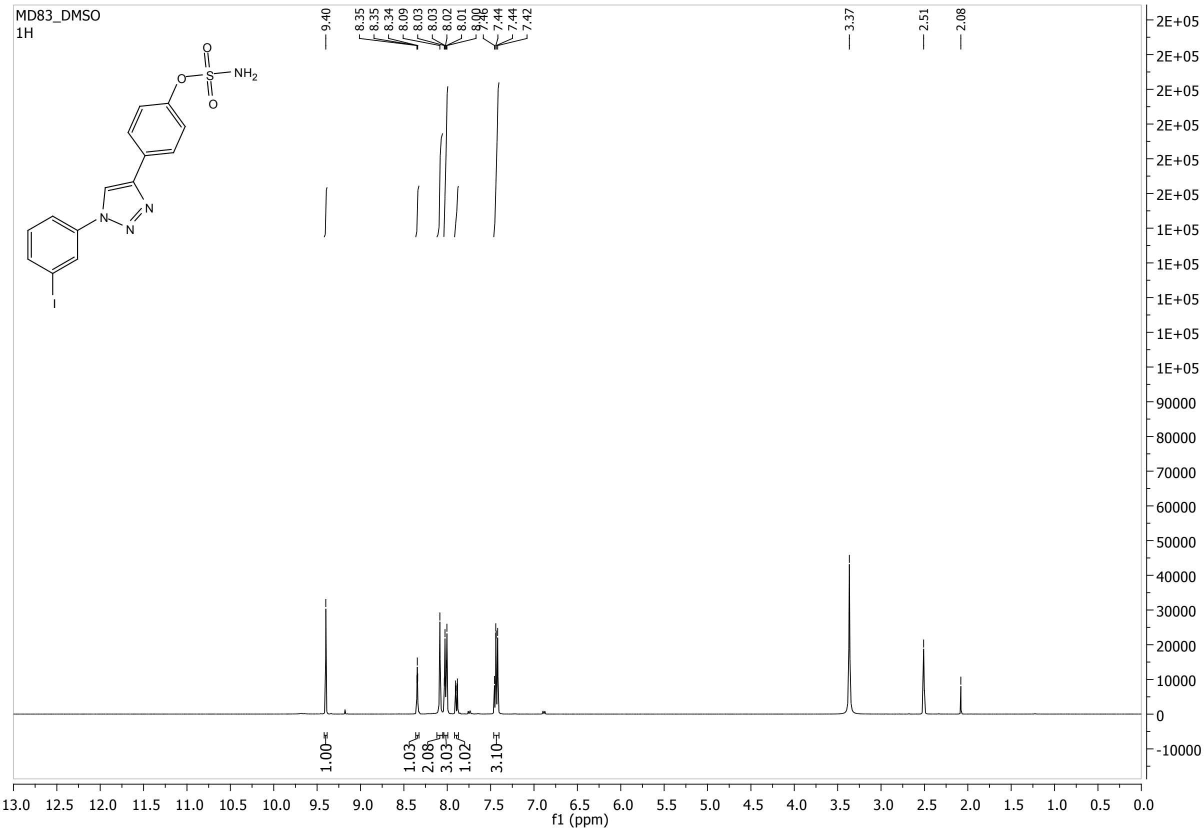

MD83_DMSO
1H
9.40
8.35
8.35
8.34
8.09
8.03
8.03
8.02
8.01
7.44
7.44
7.42
3.37
2.51
2.08
1.00
1.03
2.08
3.03
1.02
3.10
f1 (ppm)

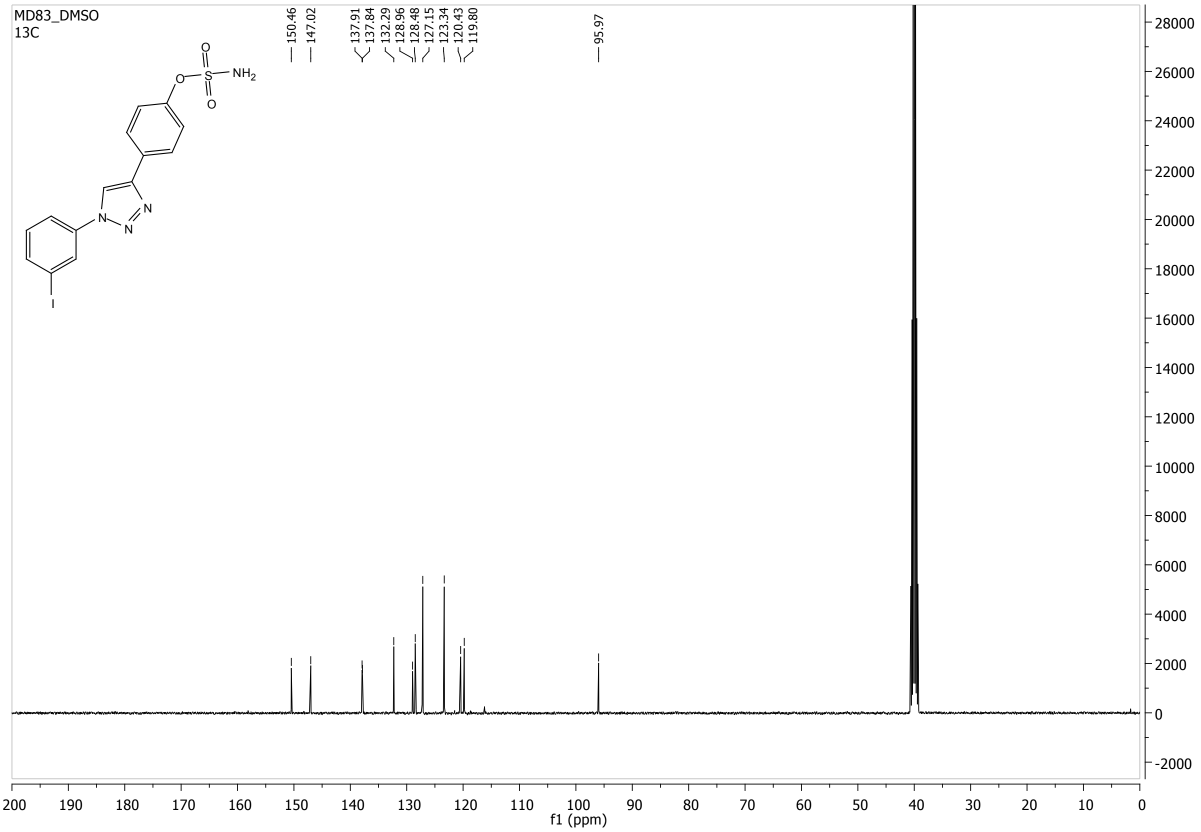

MD83_DMSO
13C
150.46
147.02
137.91
137.84
132.29
128.96
128.48
127.15
123.34
120.43
119.80
95.97
f1 (ppm)

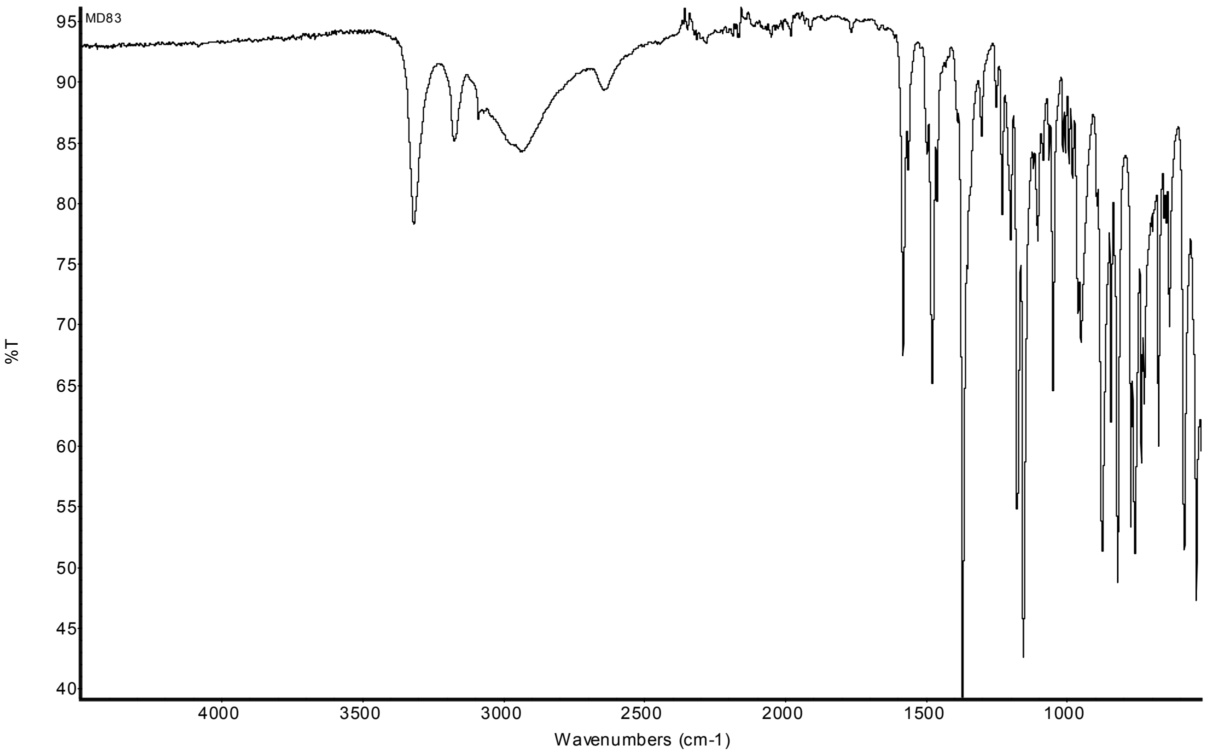

Acq. Time: 10:13
Acq. Date: Saturday, April 07, 2018

Batch Name: ManualTune.bat
Acq. File: MT20180407101353.wiff

Scan Mode: Zero Width
Polarity/Scan Type: Negative

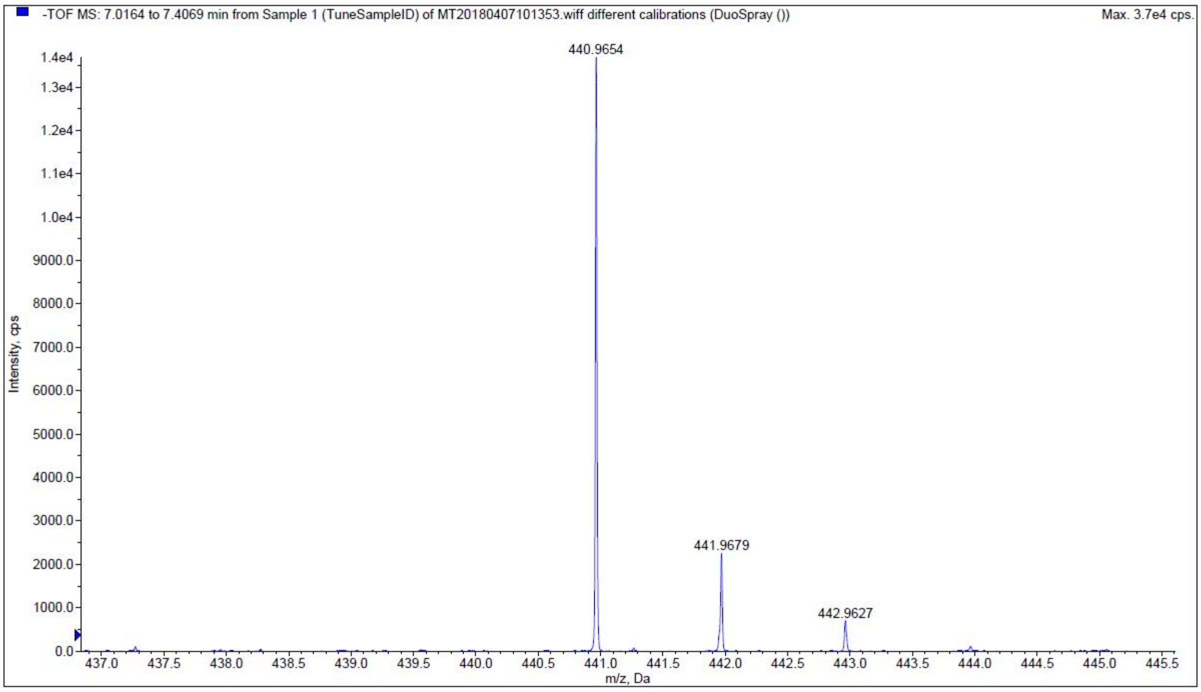

Printing Time: 10:22:35 AM
Printing Date: Saturday, April 07, 2018

Workstation: TRIPLETOF5600
Operator: Uniwersytet Gdanski

Analyst Version: 1.7.1
Page 1 of 1

HPLC purity 97.084 %

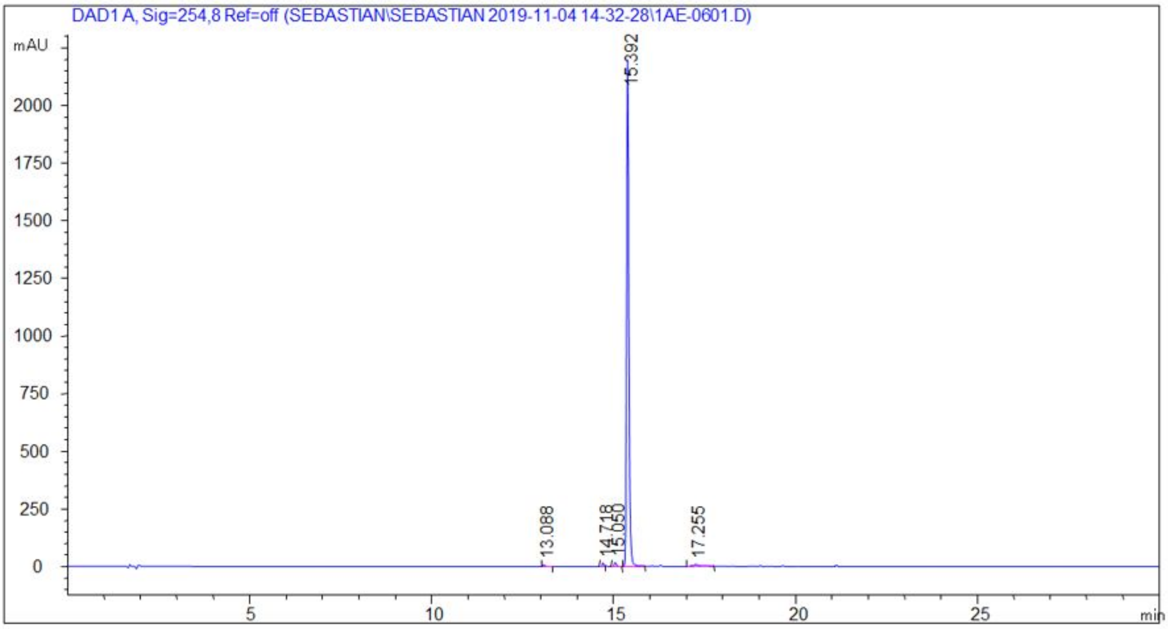

4e

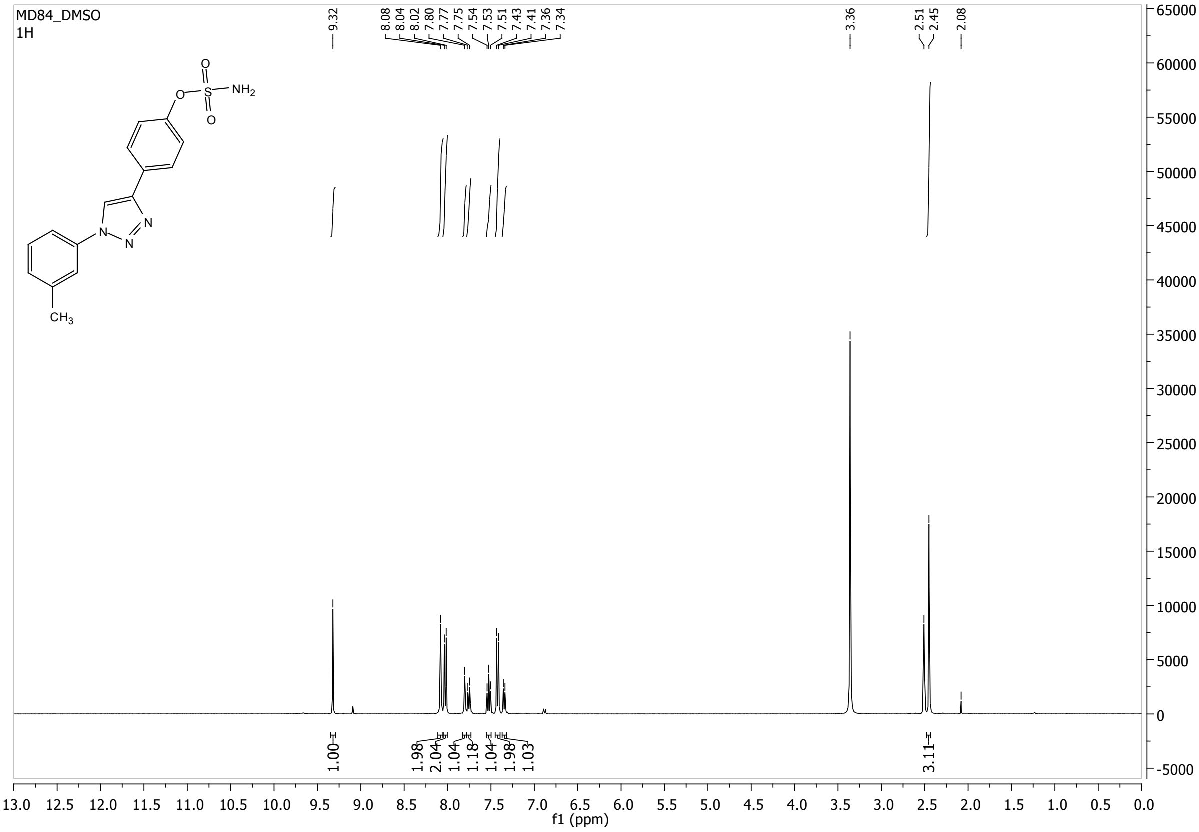
MD84_DMSO
1H
9.32
8.08 8.04 8.02 7.80 7.77 7.75 7.54 7.53 7.51 7.43 7.41 7.36 7.34
3.36
2.51 2.45
2.08
1.00
1.98 2.04 1.04 1.18 1.04 1.98 1.03
3.11
f1 (ppm)

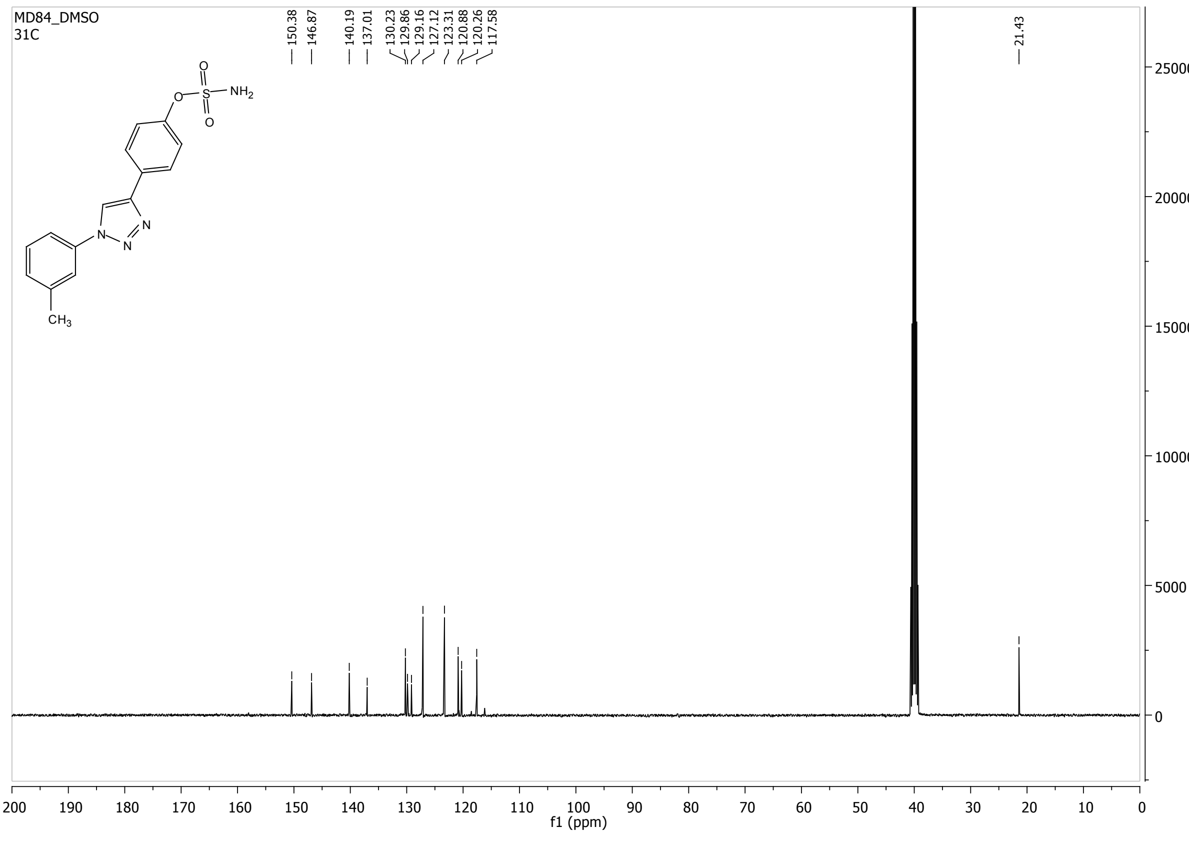
MD84_DMSO
31C
150.38 146.87 140.19 137.01 130.23 129.86 129.16 127.12 123.31 120.88 120.26 117.58
21.43
f1 (ppm)

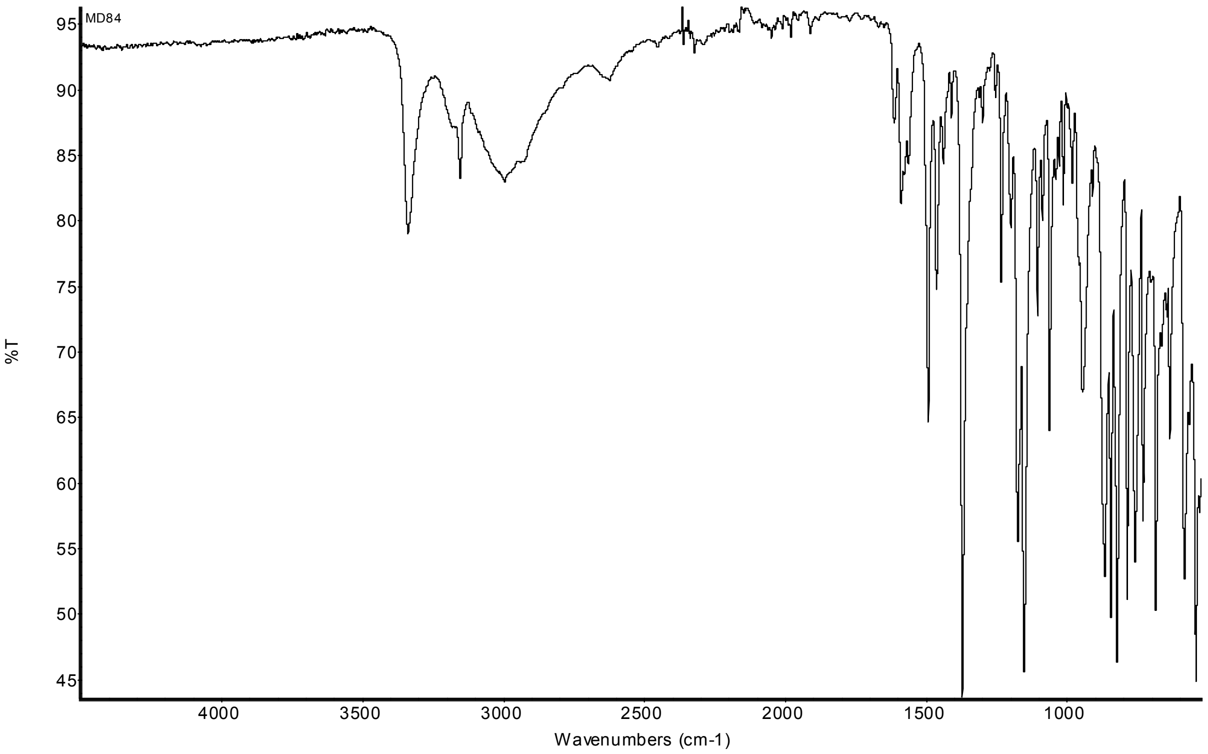

Acq. Time: 10:30
Acq. Date: Saturday, April 07, 2018

Batch Name: ManualTune.bat
Acq. File: MT20180407103028.wiff

Scan Mode: Zero Width
Polarity/Scan Type: Negative

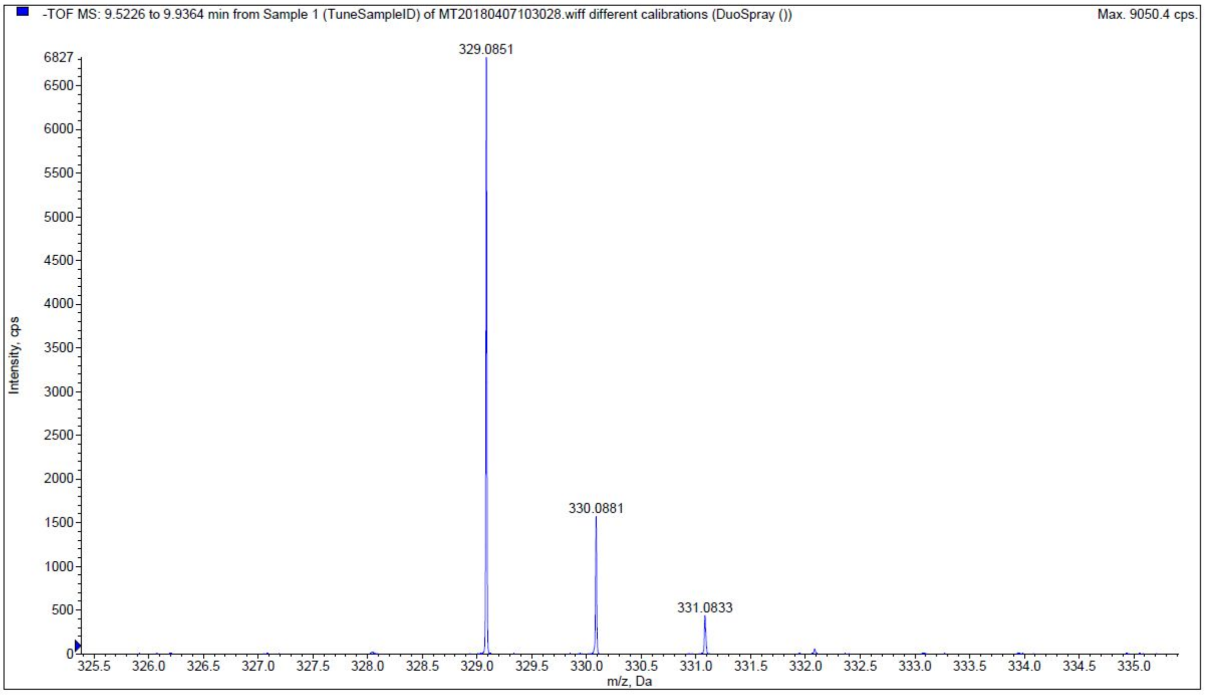

Printing Time: 10:41:33 AM
Printing Date: Saturday, April 07, 2018

Workstation: TRIPLETOF5600
Operator: Uniwersytet Gdanski

Analyst Version: 1.7.1
Page 1 of 1

HPLC purity 99.230 %

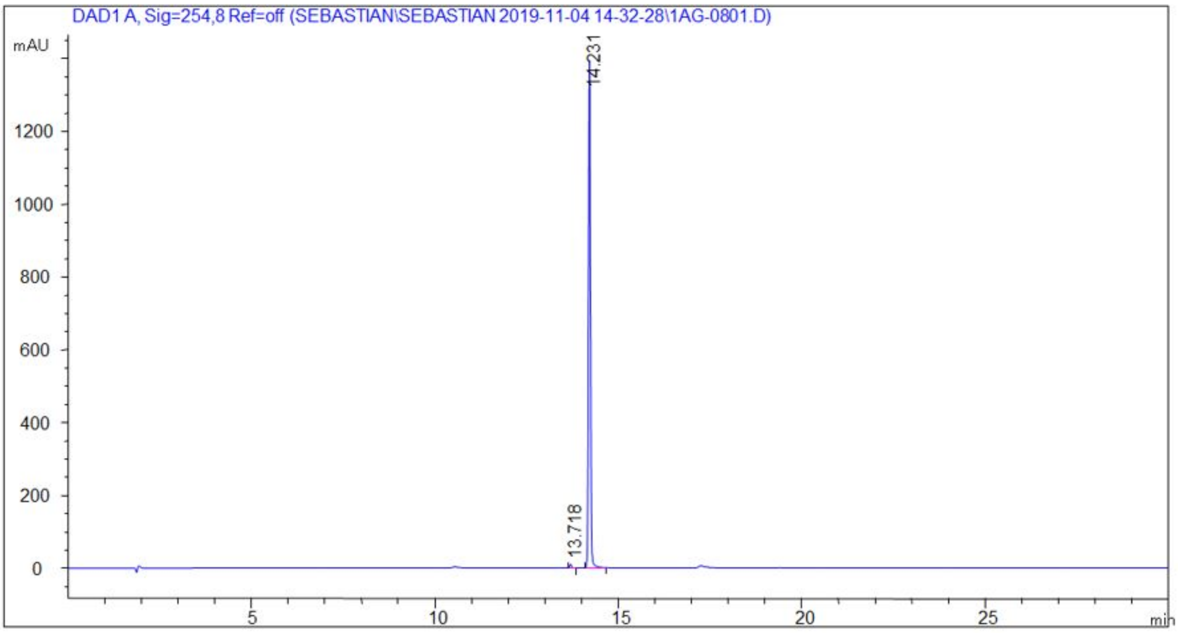

4f

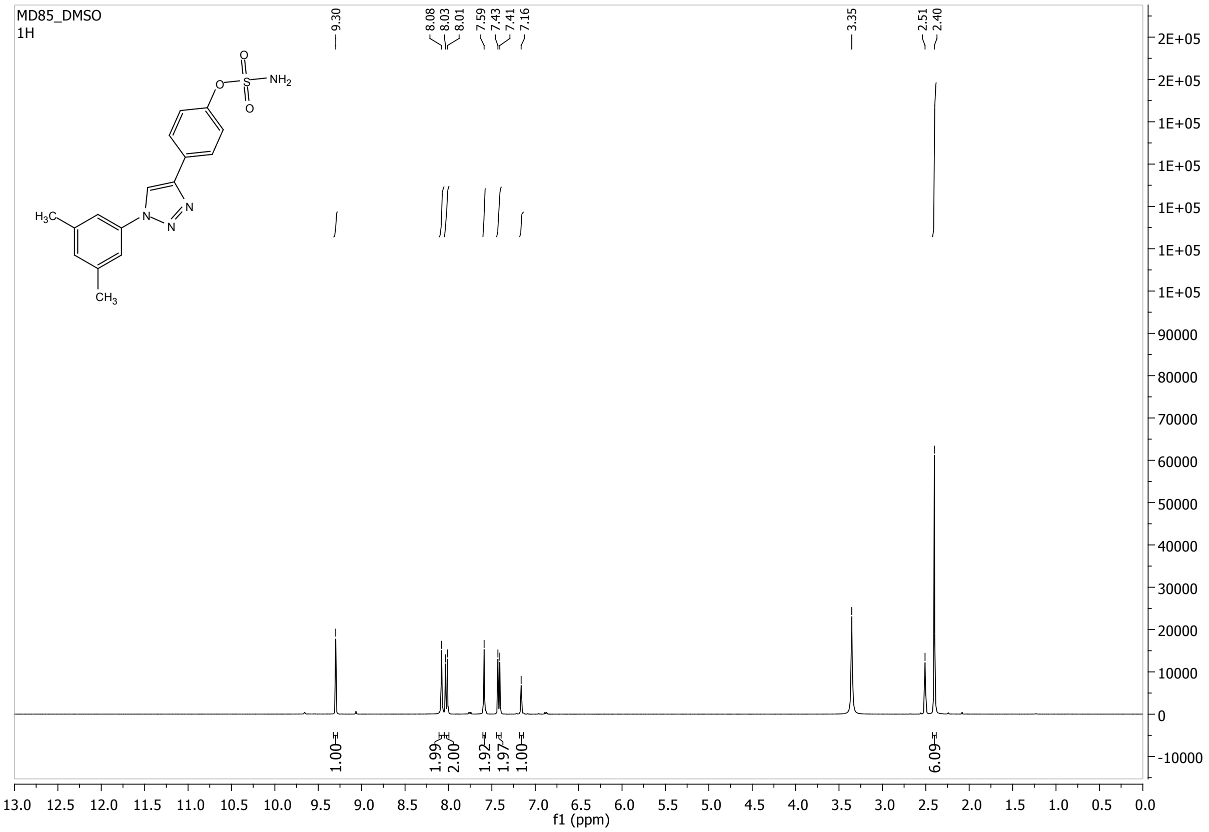
MD85_DMSO
1H
9.30
8.08
8.03
8.01
7.59
7.43
7.41
7.16
3.35
2.51
2.40
1.00
1.99
2.00
1.92
1.97
1.00
6.09
f1 (ppm)

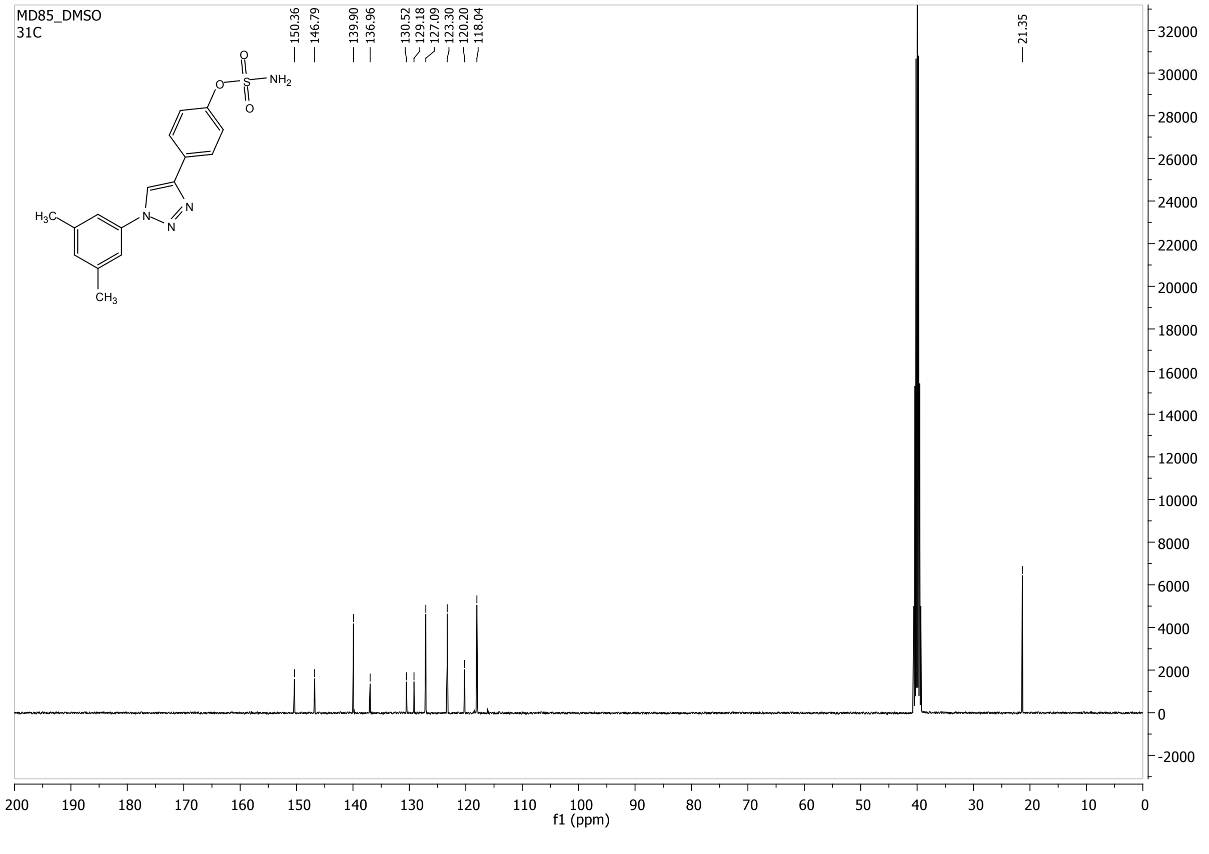
MD85_DMSO
31C
150.36
146.79
139.90
136.96
130.52
129.18
127.09
123.30
120.20
118.04
21.35
f1 (ppm)

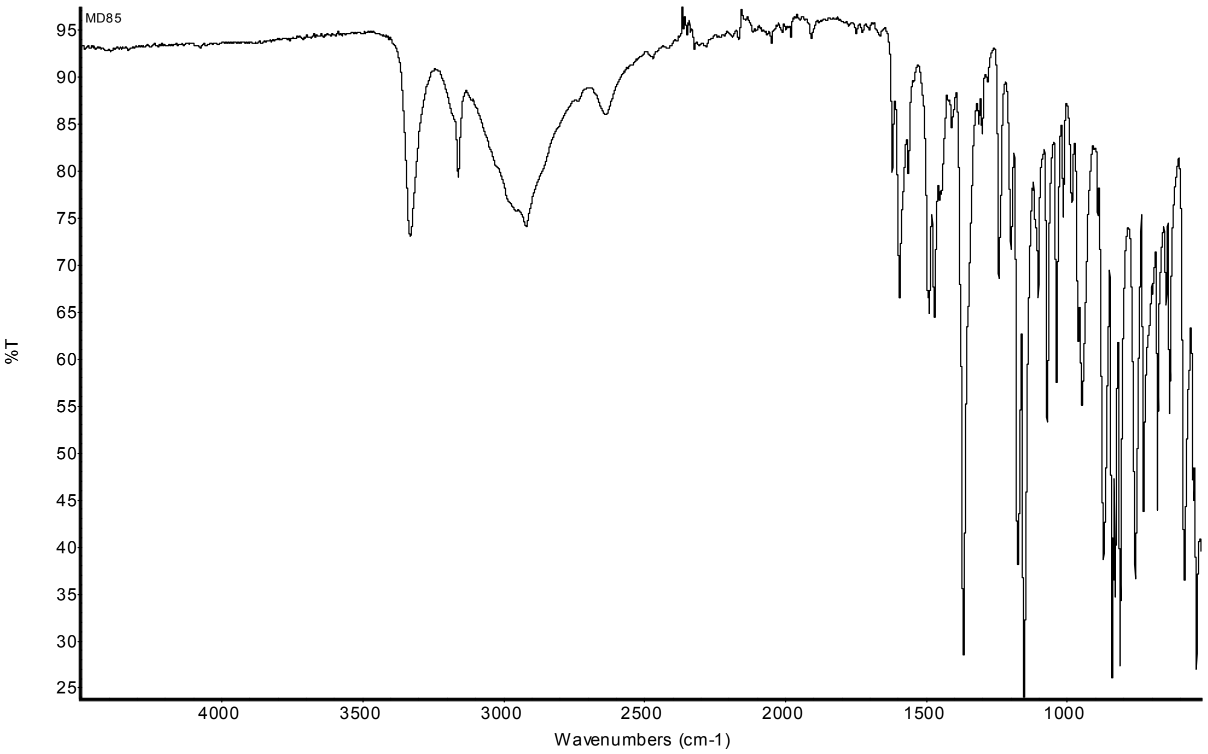

Acq. Time: 10:30
Acq. Date: Saturday, April 07, 2018

Batch Name: ManualTune.bat
Acq. File: MT20180407103028.wiff

Scan Mode: Zero Width
Polarity/Scan Type: Negative

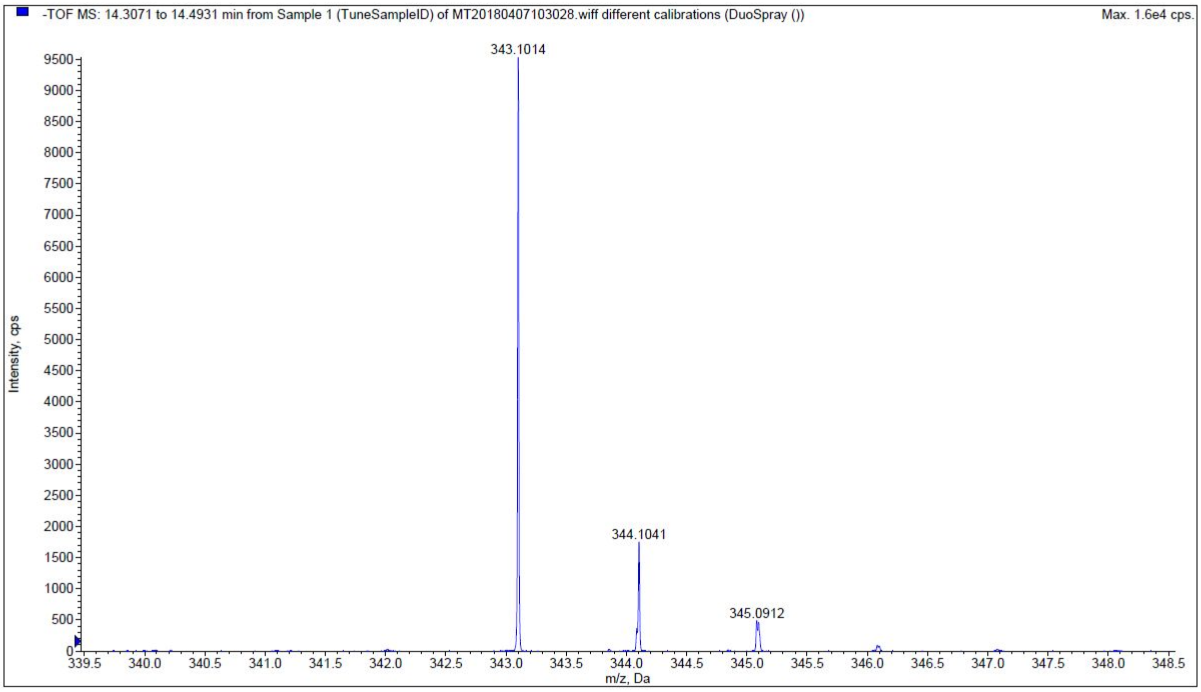

Printing Time: 10:46:10 AM
Printing Date: Saturday, April 07, 2018

Workstation: TRIPLETOF5600
Operator: Uniwersytet Gdanski

Analyst Version: 1.7.1
Page 1 of 1

HPLC purity 99.725 %

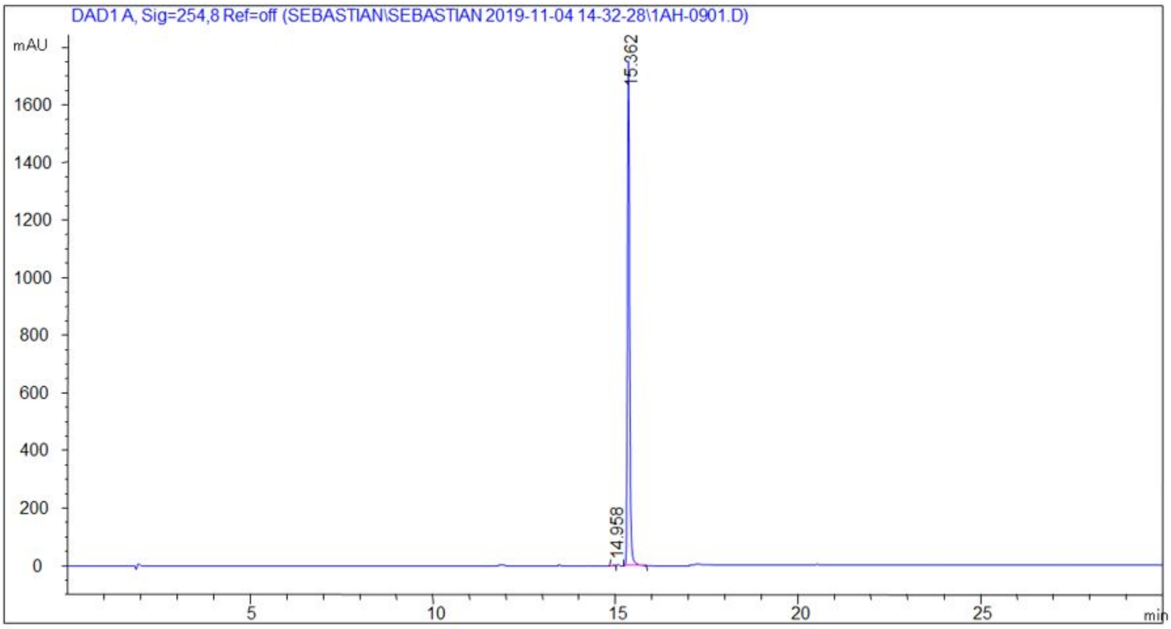

4g

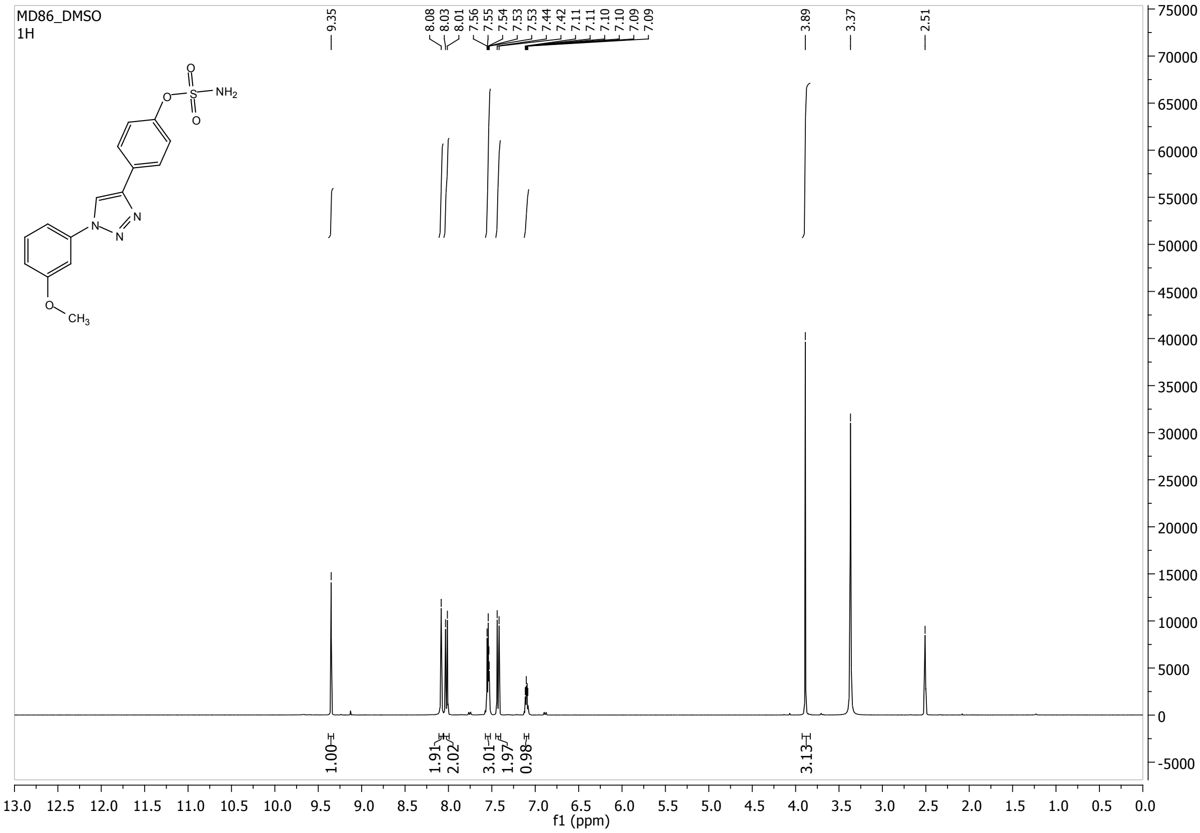
MD86_DMSO
1H
9.35
8.08
8.03
8.01
7.56
7.55
7.54
7.53
7.53
7.44
7.42
7.11
7.11
7.10
7.09
7.09
3.89
3.37
2.51
1.00
1.91
2.02
3.01
1.97
0.98
3.13
f1 (ppm)

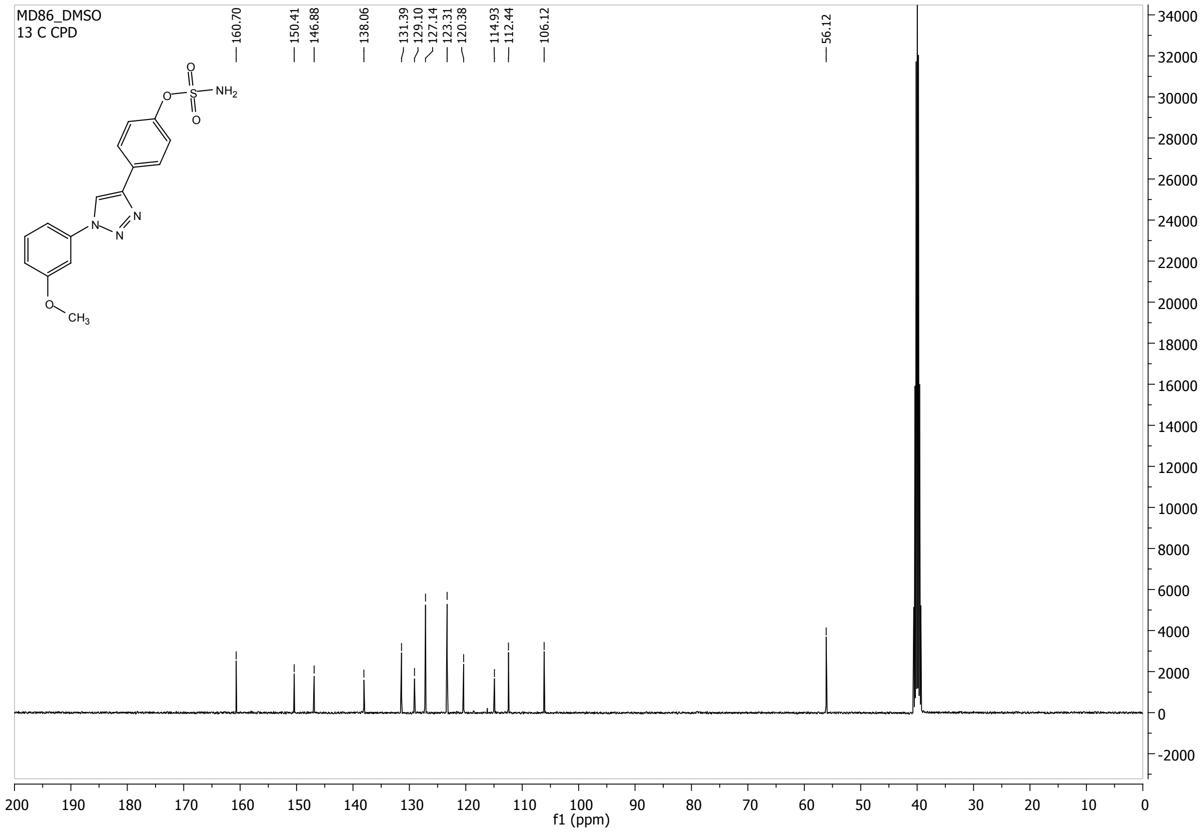
MD86_DMSO
13 C CPD
160.70
150.41
146.88
138.06
131.39
129.10
127.14
123.31
120.38
114.93
112.44
106.12
56.12
f1 (ppm)

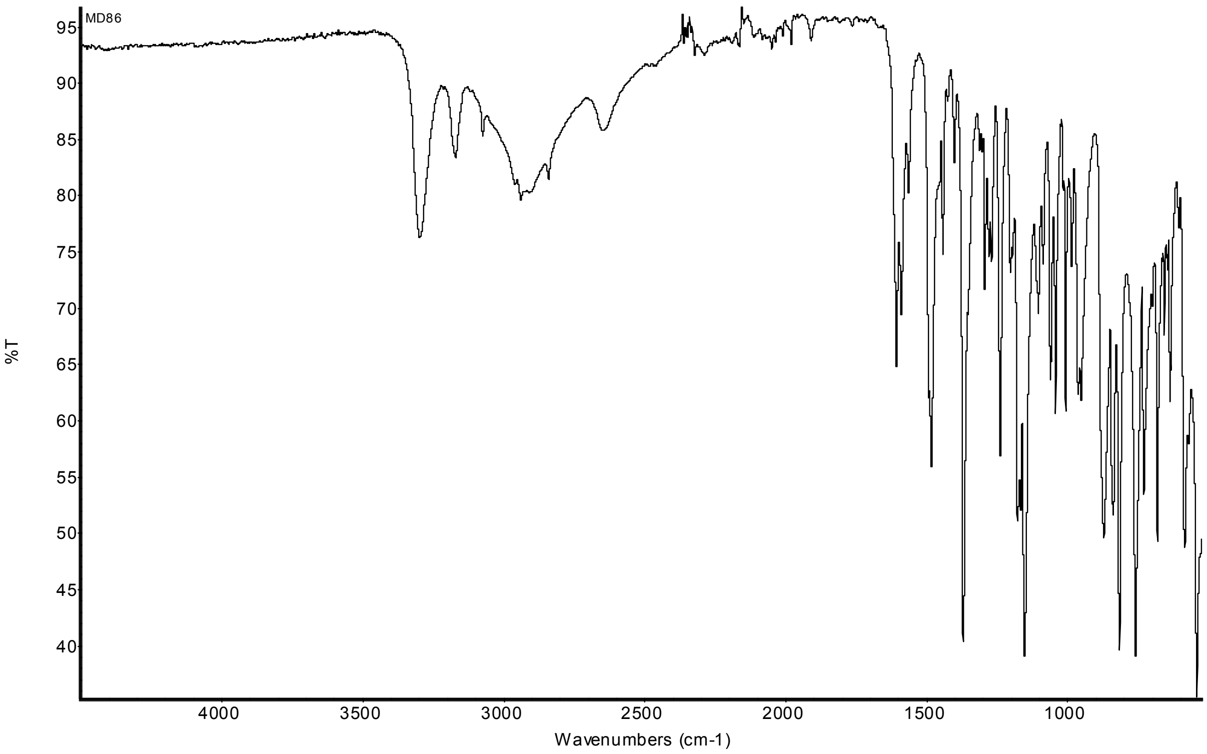

Acq. Time: 10:30
Acq. Date: Saturday, April 07, 2018

Batch Name: ManualTune.bat
Acq. File: MT20180407103028.wiff

Scan Mode: Zero Width
Polarity/Scan Type: Negative

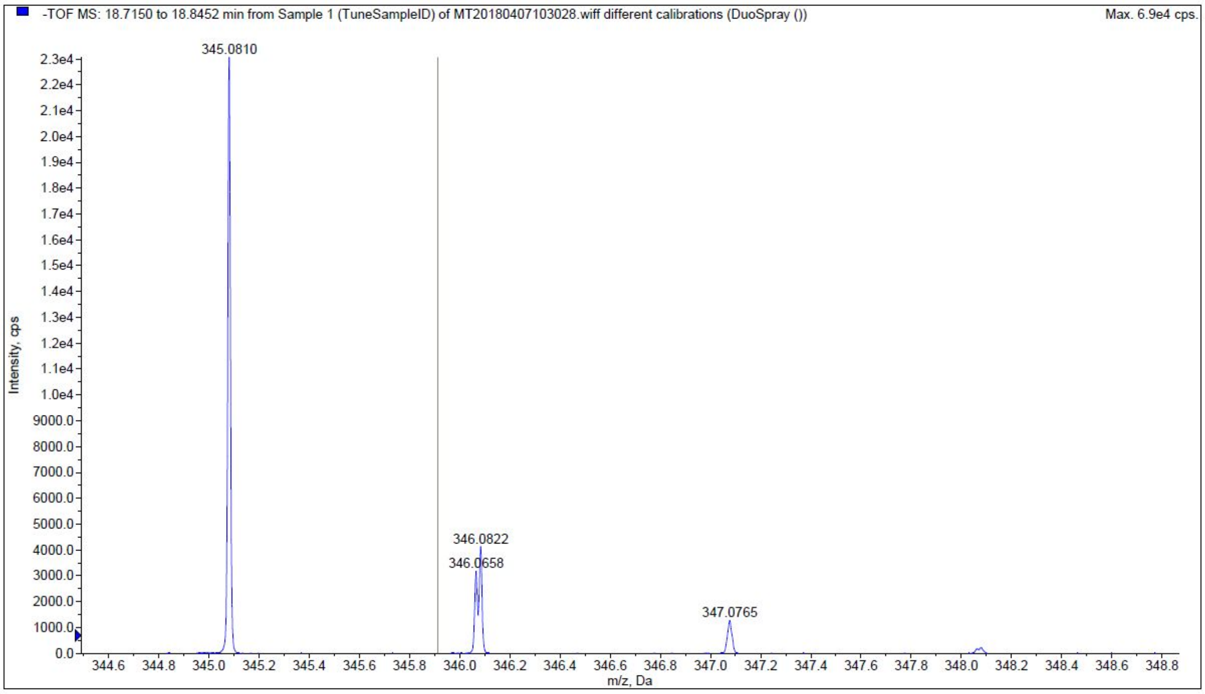

Printing Time: 10:50:33 AM
Printing Date: Saturday, April 07, 2018

Workstation: TRIPLETOF5600
Operator: Uniwersytet Gdanski

Analyst Version: 1.7.1
Page 1 of 1

HPLC purity 97.586 %

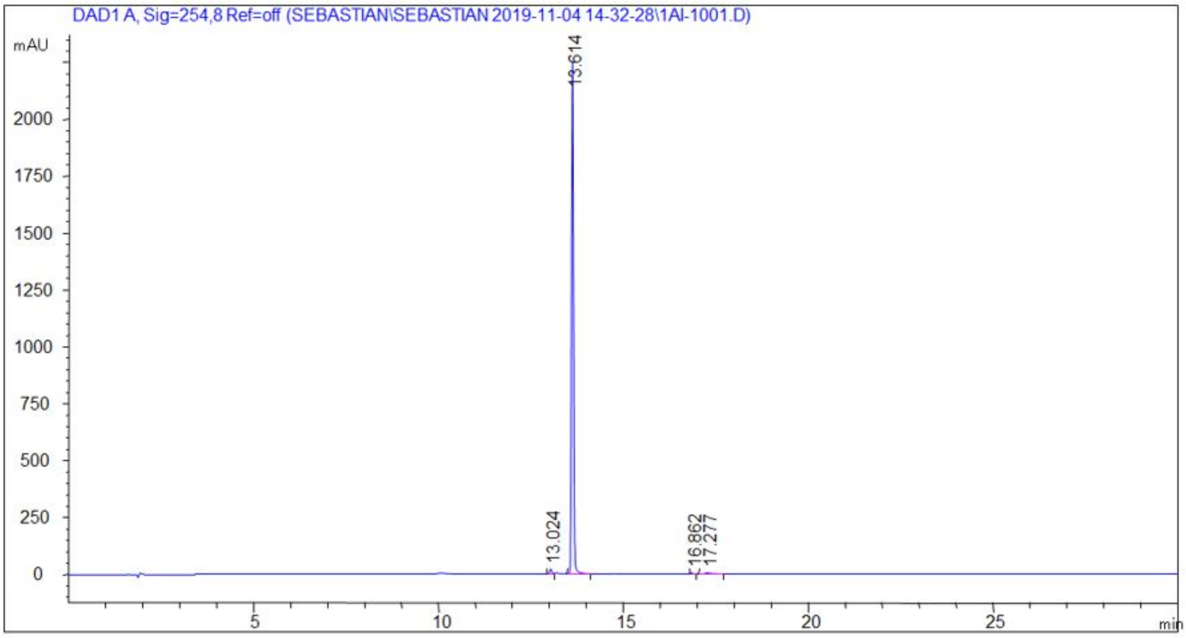

4h

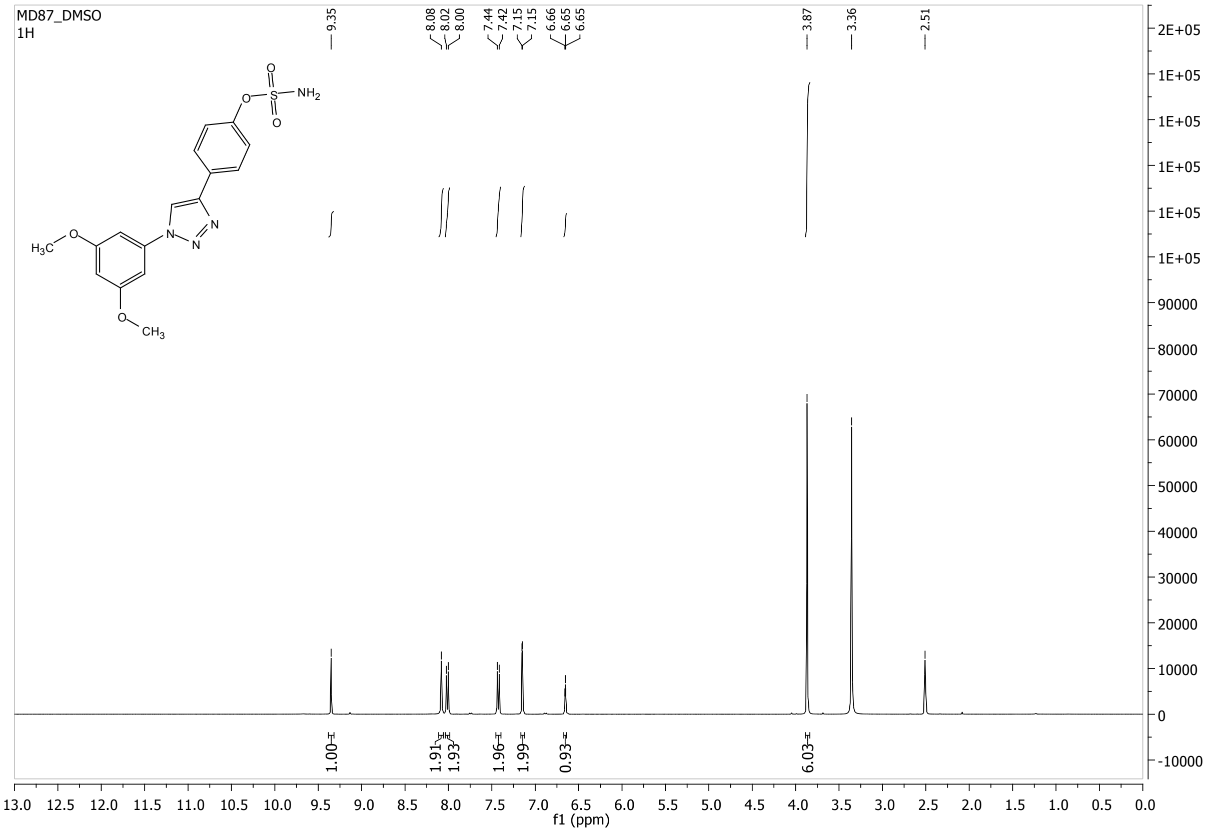
MD87_DMSO
1H
9.35
8.08
8.02
8.00
7.44
7.42
7.15
7.15
6.66
6.65
6.65
3.87
3.36
2.51
1.00
1.91
1.93
1.96
1.99
0.93
6.03
f1 (ppm)

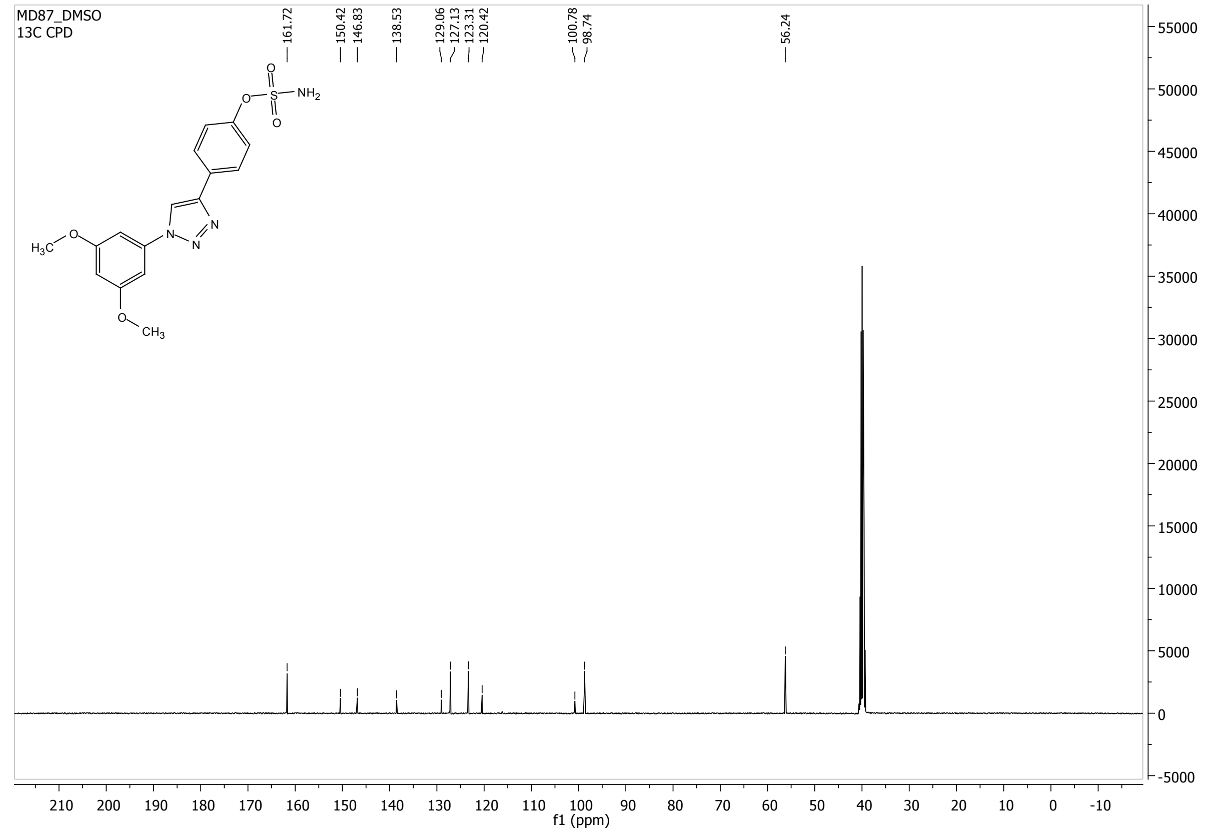
MD87_DMSO
13C CPD
161.72
150.42
146.83
138.53
129.06
127.13
123.31
120.42
100.78
98.74
56.24
f1 (ppm)

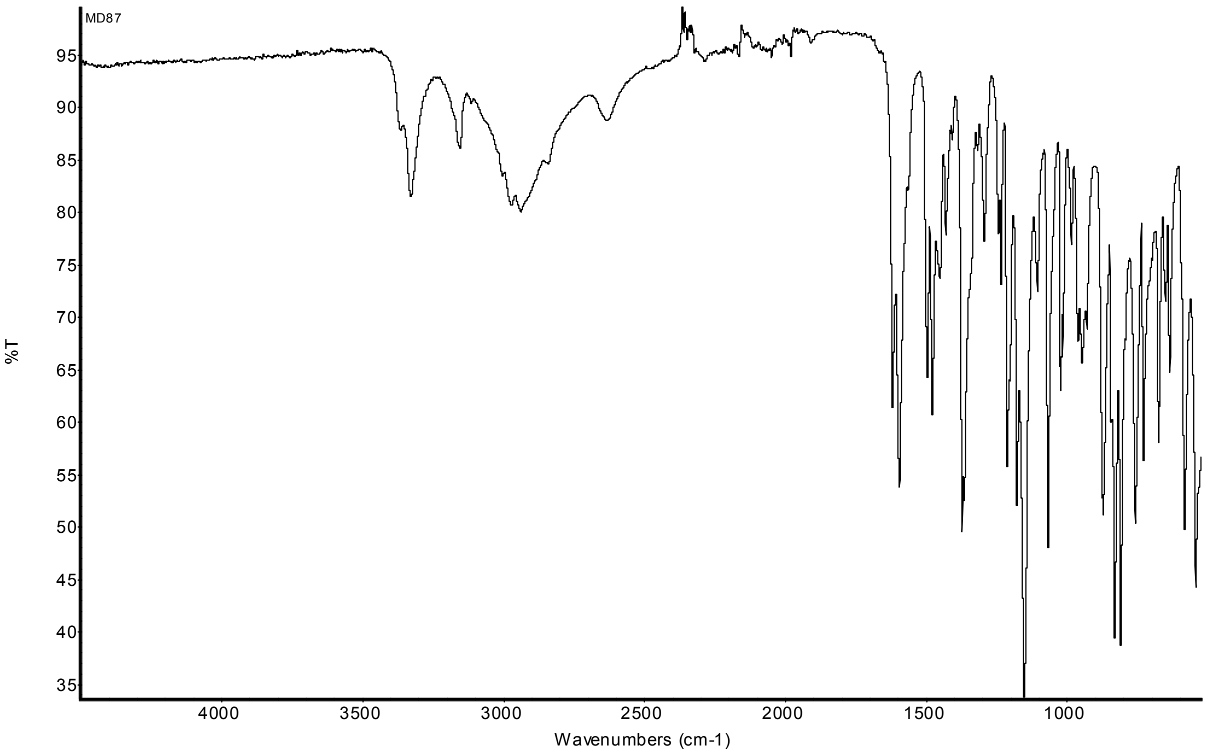

Acq. Time: 10:30
Acq. Date: Saturday, April 07, 2018

Batch Name: ManualTune.bat
Acq. File: MT20180407103028.wiff

Scan Mode: Zero Width
Polarity/Scan Type: Negative

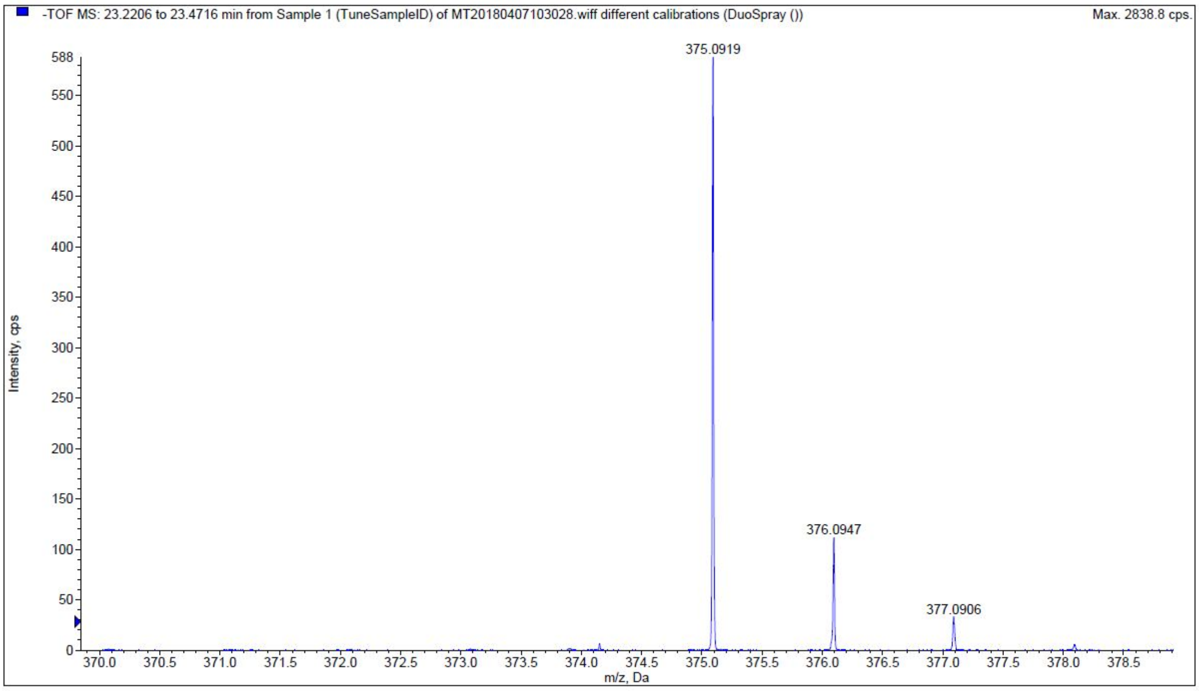

Printing Time: 10:55:01 AM
Printing Date: Saturday, April 07, 2018

Workstation: TRIPLETOF5600
Operator: Uniwersytet Gdanski

Analyst Version: 1.7.1
Page 1 of 1

HPLC purity 98.573 %

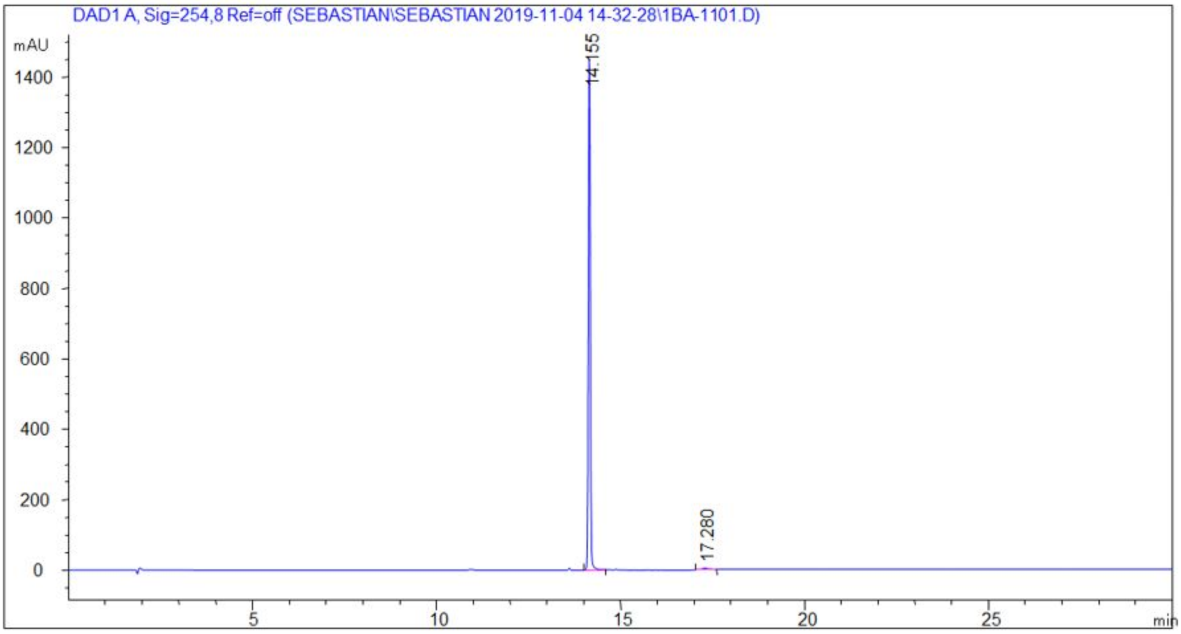

4i

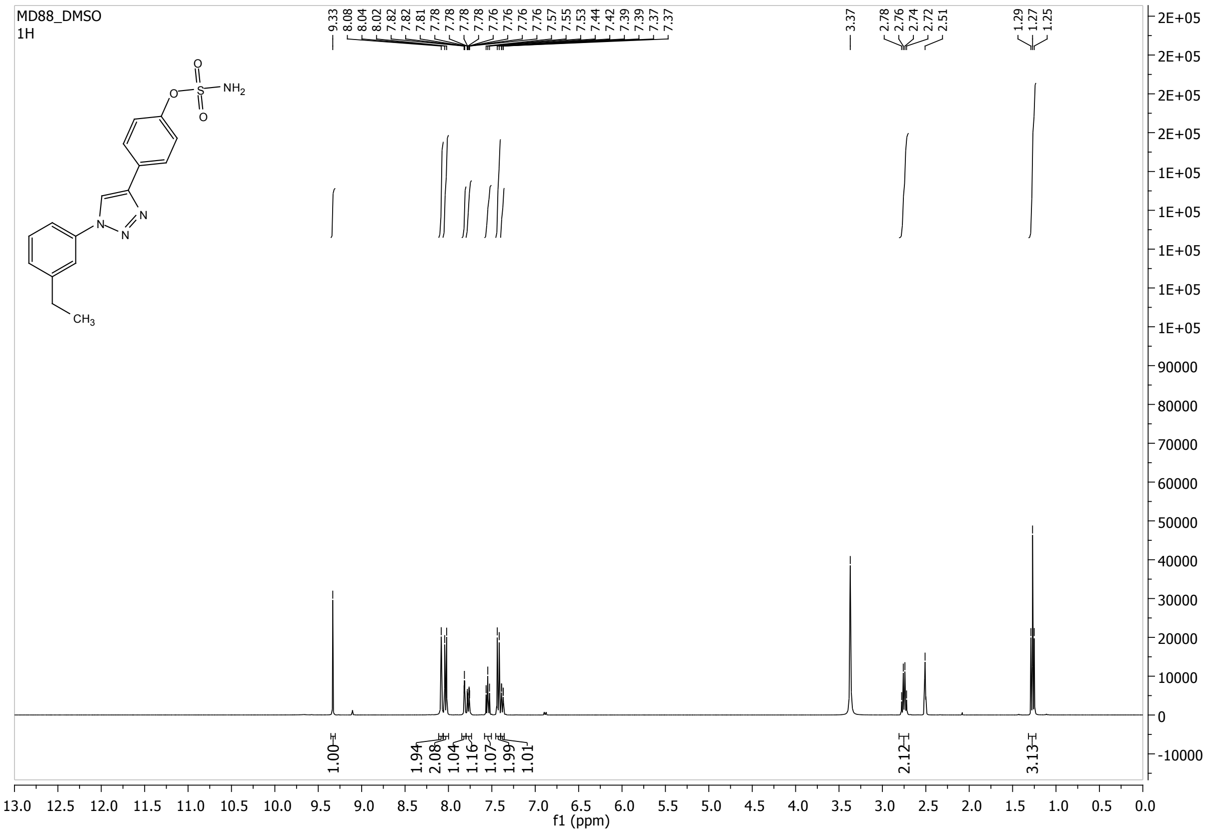
MD88_DMSO
1H
9.33
8.08
8.04
8.02
7.82
7.82
7.81
7.78
7.78
7.78
7.78
7.76
7.76
7.76
7.76
7.57
7.55
7.53
7.44
7.42
7.39
7.39
7.37
7.37
3.37
2.78
2.76
2.74
2.72
2.51
1.29
1.27
1.25
1.00
1.94
2.08
1.04
1.16
1.07
1.99
1.01
2.12
3.13
NH2
CH3
f1 (ppm)

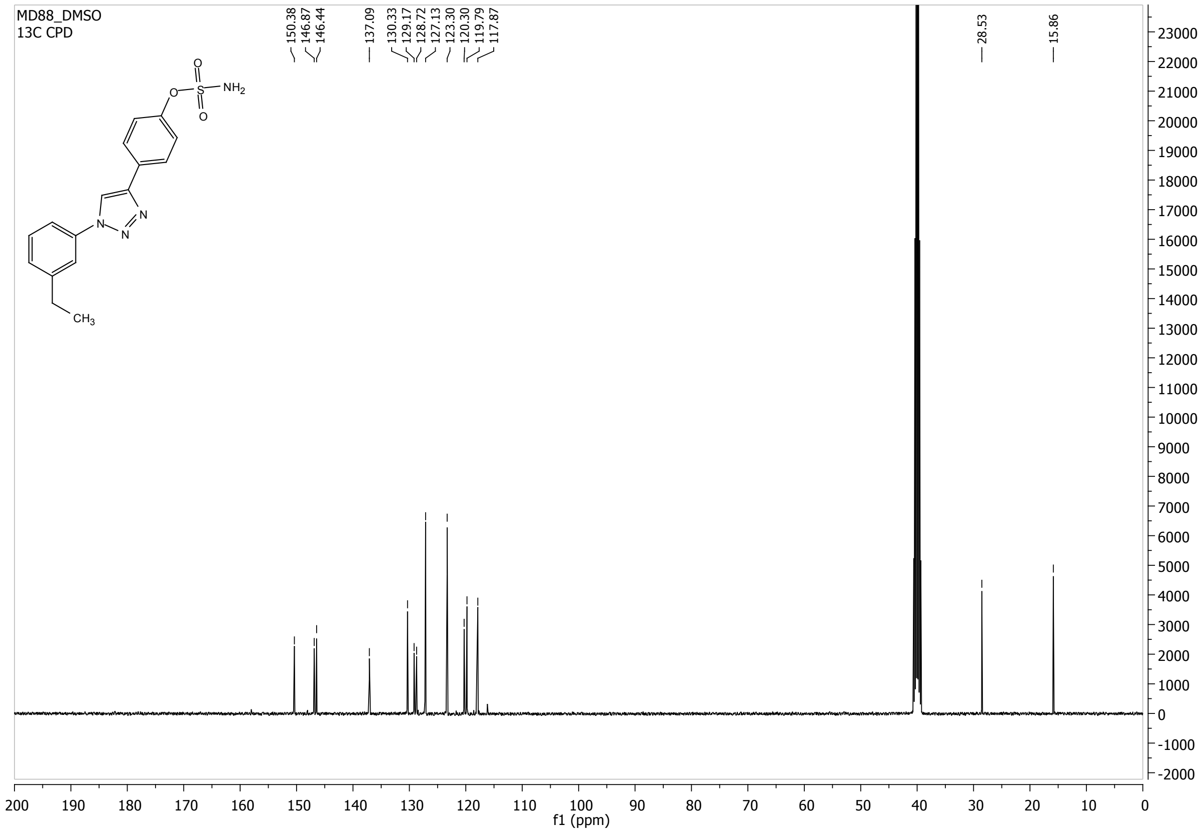
MD88_DMSO
13C CPD
150.38
146.87
146.44
137.09
130.33
129.17
128.72
127.13
123.30
120.30
119.79
117.87
28.53
15.86
NH2
CH3
f1 (ppm)

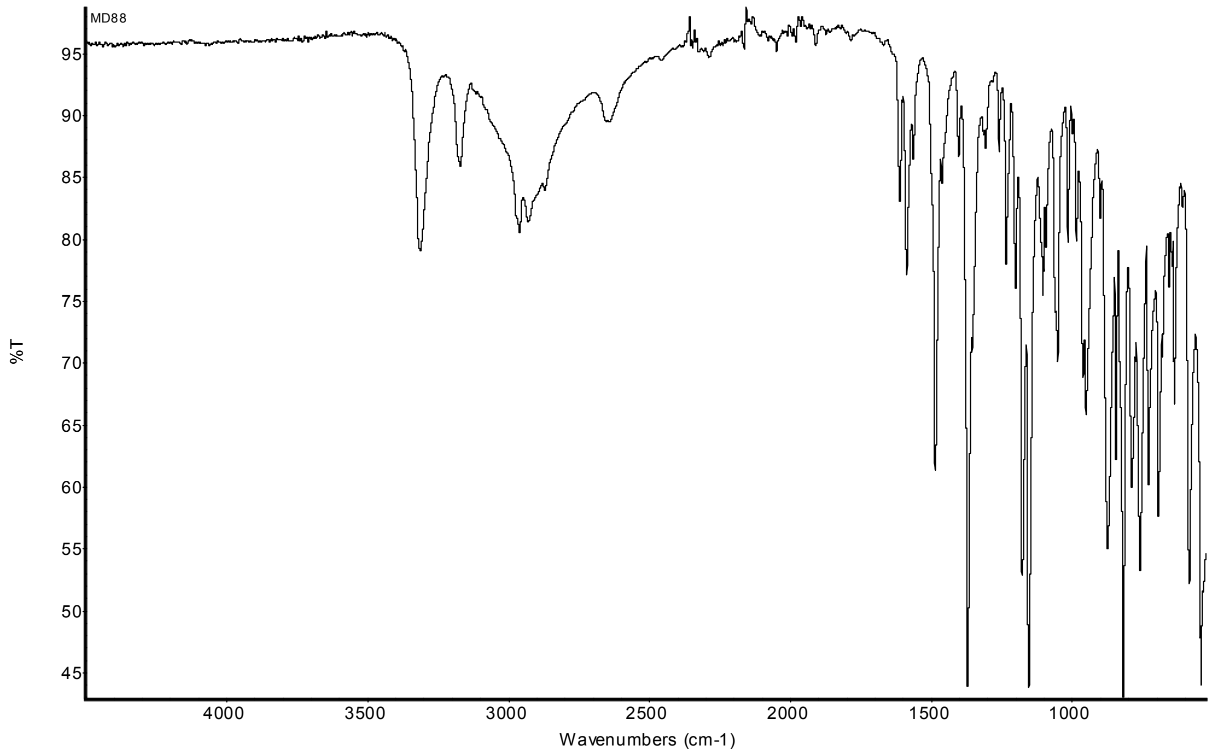

Acq. Time: 10:30
Acq. Date: Saturday, April 07, 2018

Batch Name: ManualTune.bat
Acq. File: MT20180407103028.wiff

Scan Mode: Zero Width
Polarity/Scan Type: Negative

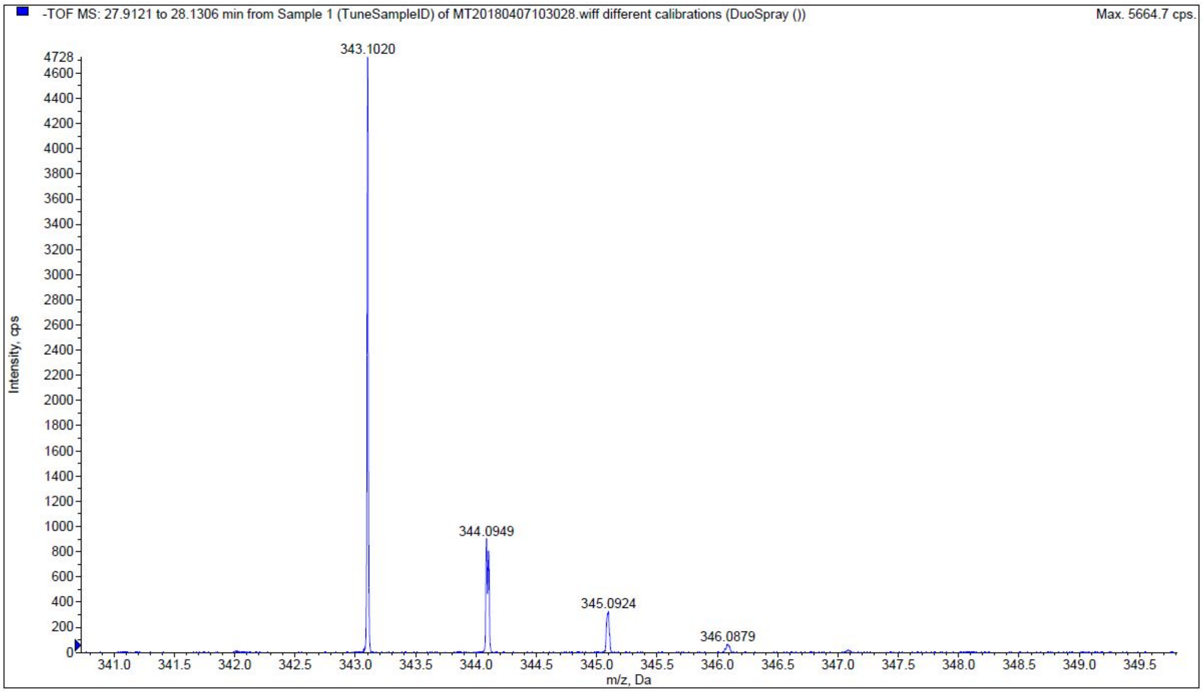

Printing Time: 11:00:04 AM
Printing Date: Saturday, April 07, 2018

Workstation: TRIPLETOF5600
Operator: Uniwersytet Gdanski

Analyst Version: 1.7.1
Page 1 of 1

HPLC purity 100.000 %

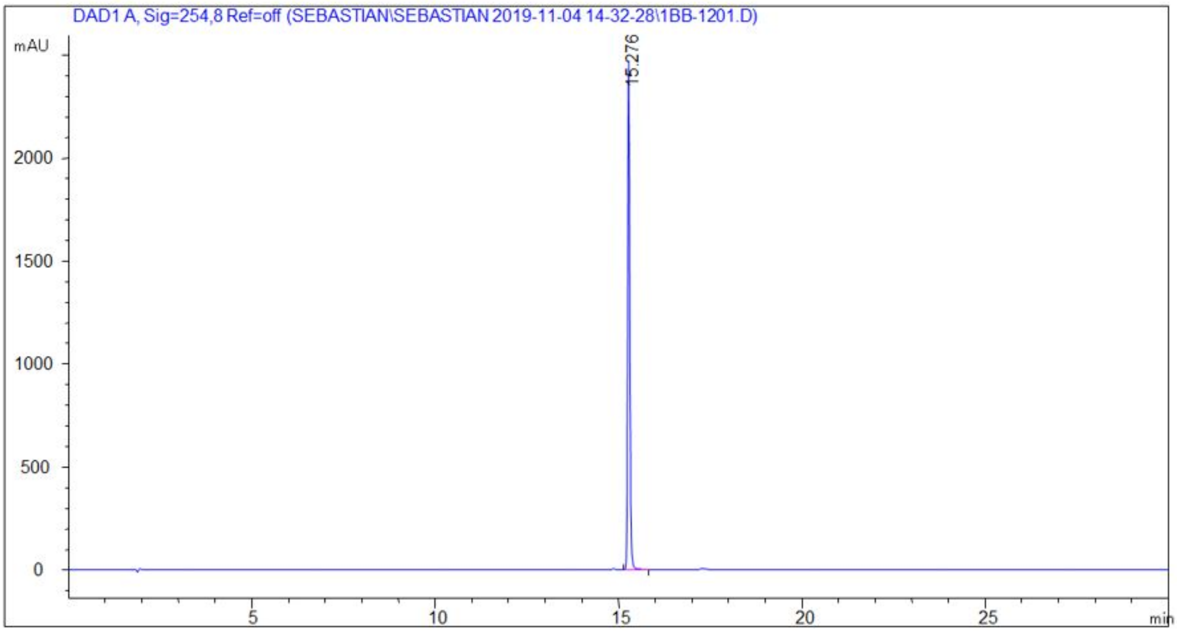

4j

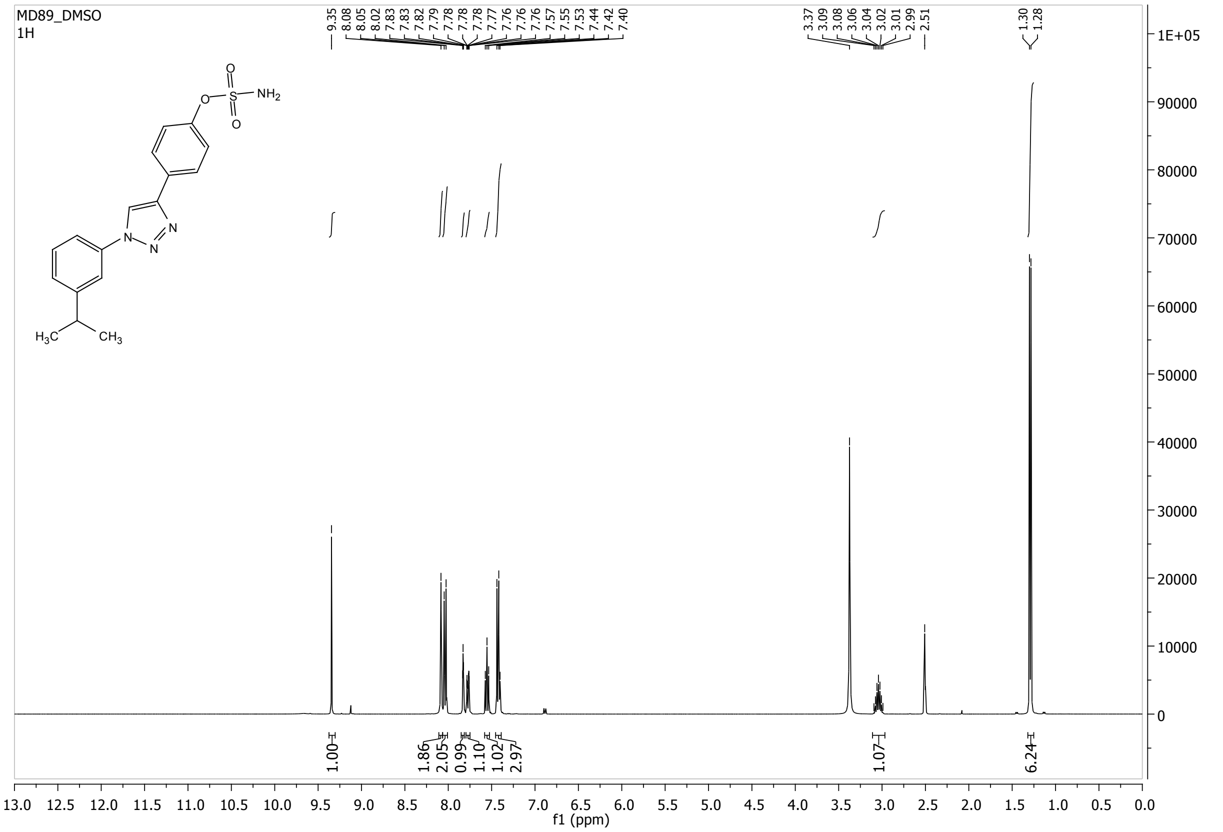
MD89_DMSO
1H
9.35
8.08
8.05
8.02
7.83
7.83
7.82
7.79
7.78
7.78
7.78
7.77
7.76
7.76
7.76
7.57
7.55
7.53
7.44
7.42
7.40
3.37
3.09
3.08
3.06
3.04
3.02
3.01
2.99
2.51
1.30
1.28
1.00
1.86
2.05
0.99
1.10
1.02
2.97
1.07
6.24
f1 (ppm)

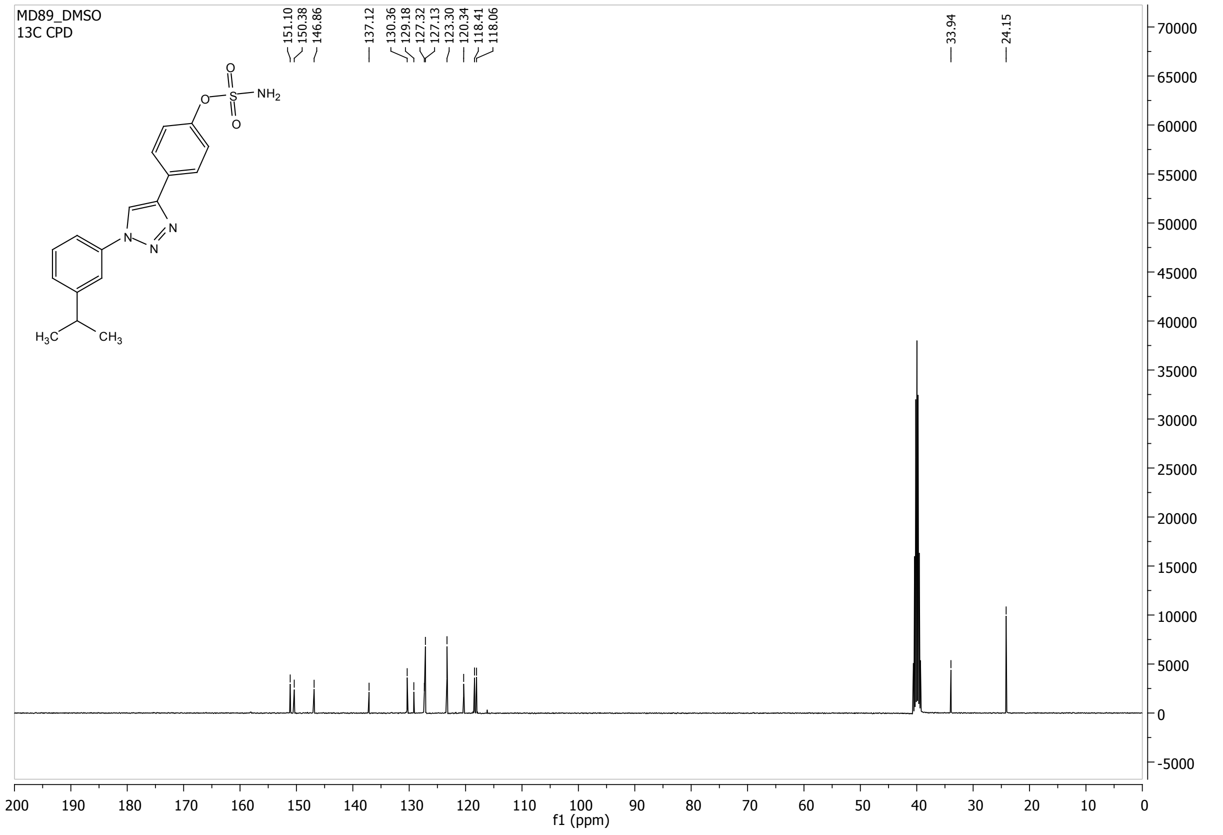
MD89_DMSO
13C CPD
151.10
150.38
146.86
137.12
130.36
129.18
127.32
127.13
123.30
120.34
118.41
118.06
33.94
24.15
f1 (ppm)

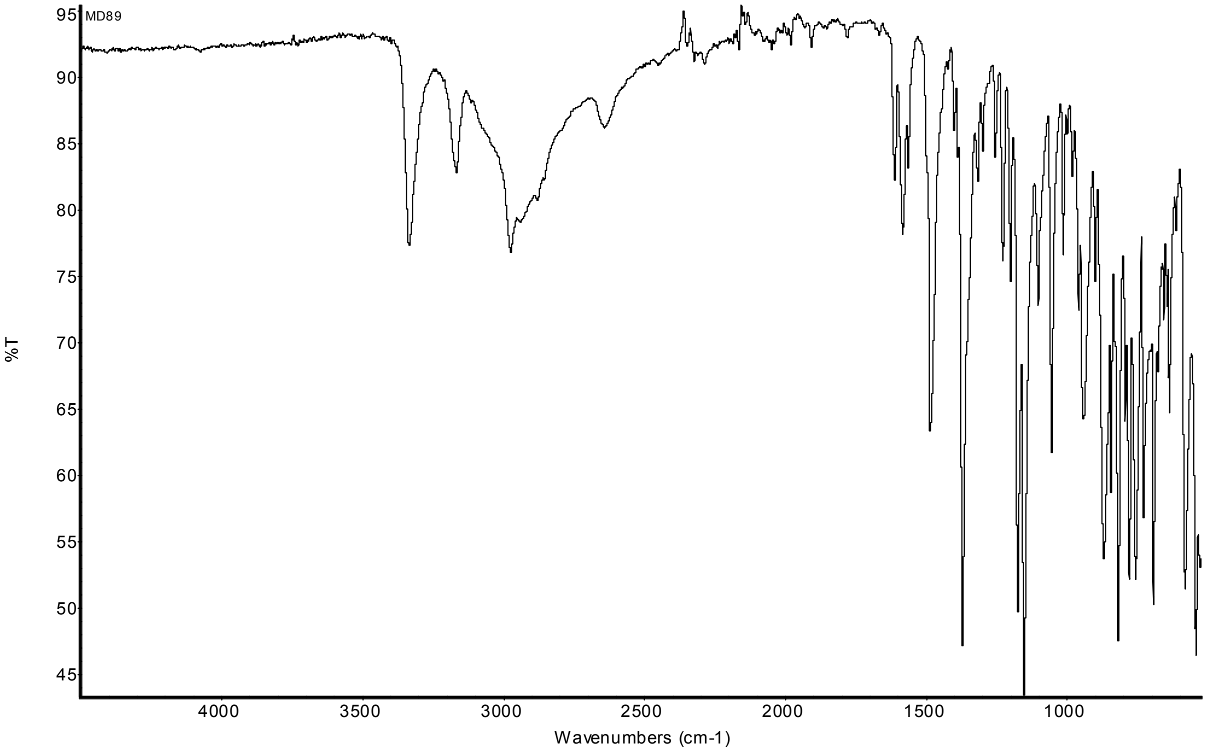

Acq. Time: 10:30
Acq. Date: Saturday, April 07, 2018

Batch Name: ManualTune.bat
Acq. File: MT20180407103028.wiff

Scan Mode: Zero Width
Polarity/Scan Type: Negative

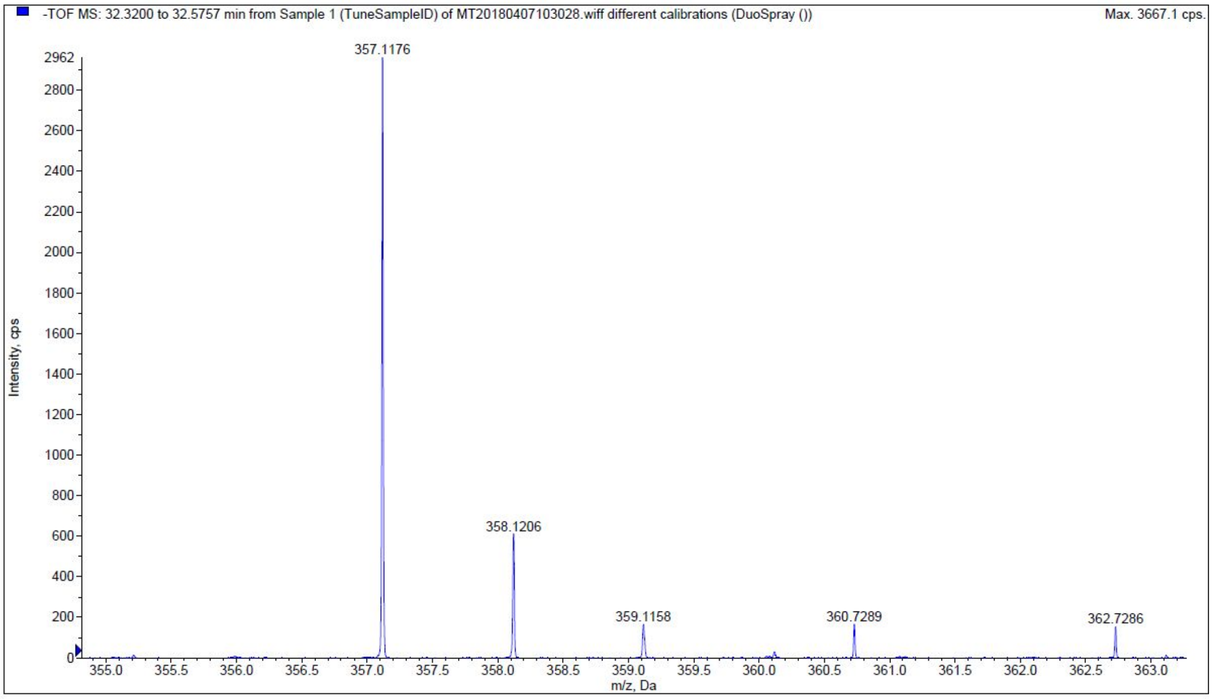

Printing Time: 11:04:21 AM
Printing Date: Saturday, April 07, 2018

Workstation: TRIPLETOF5600
Operator: Uniwersytet Gdanski

Analyst Version: 1.7.1
Page 1 of 1

HPLC purity 99.219 %

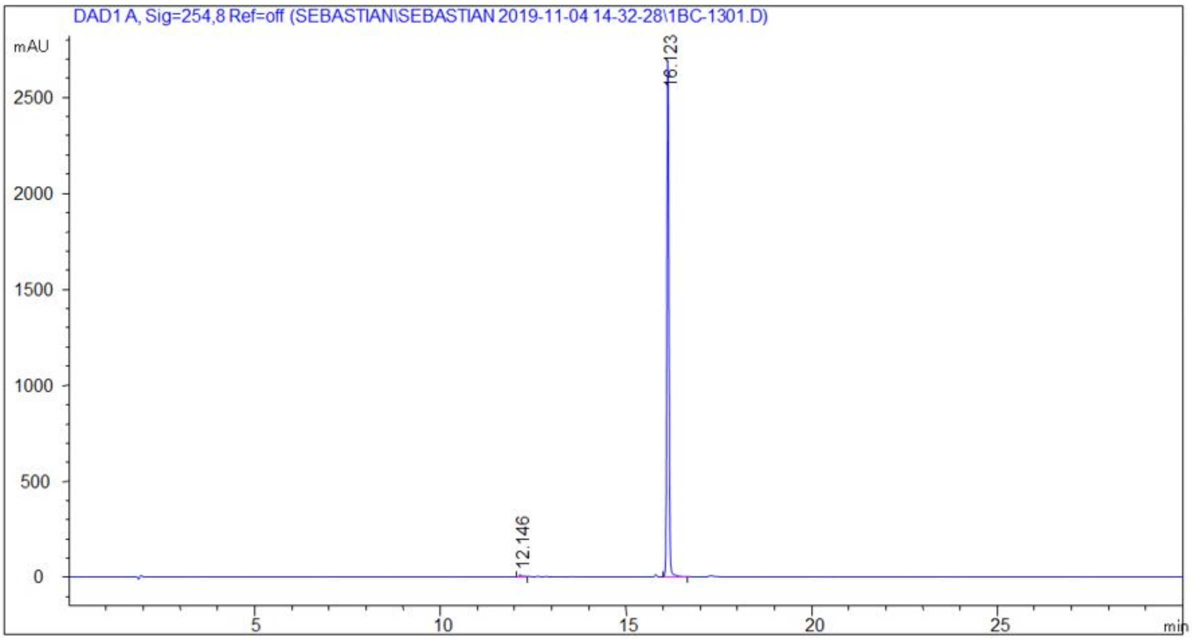

4k

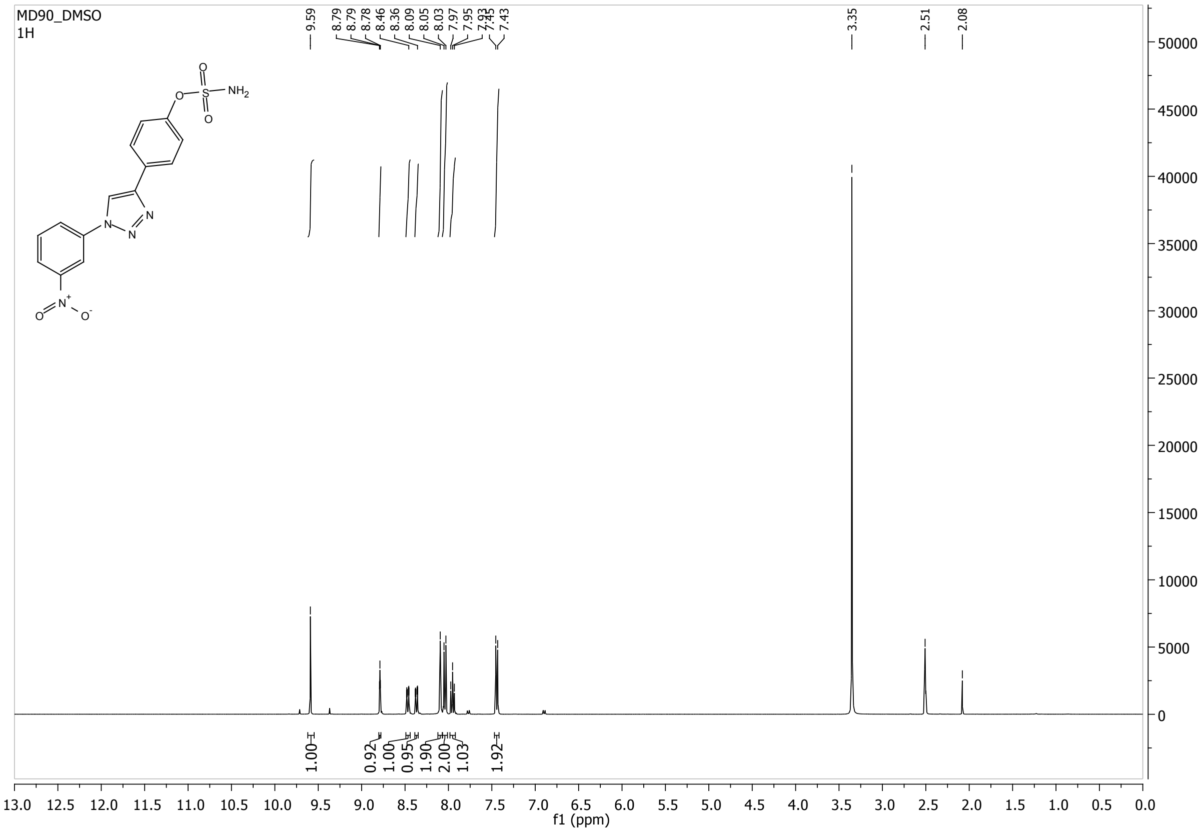
MD90_DMSO
1H
9.59
8.79
8.79
8.78
8.46
8.36
8.09
8.05
8.03
7.97
7.95
7.93
7.45
7.43
3.35
2.51
2.08
NH2
1.00
0.92
1.00
0.95
1.90
2.00
1.03
1.92
f1 (ppm)

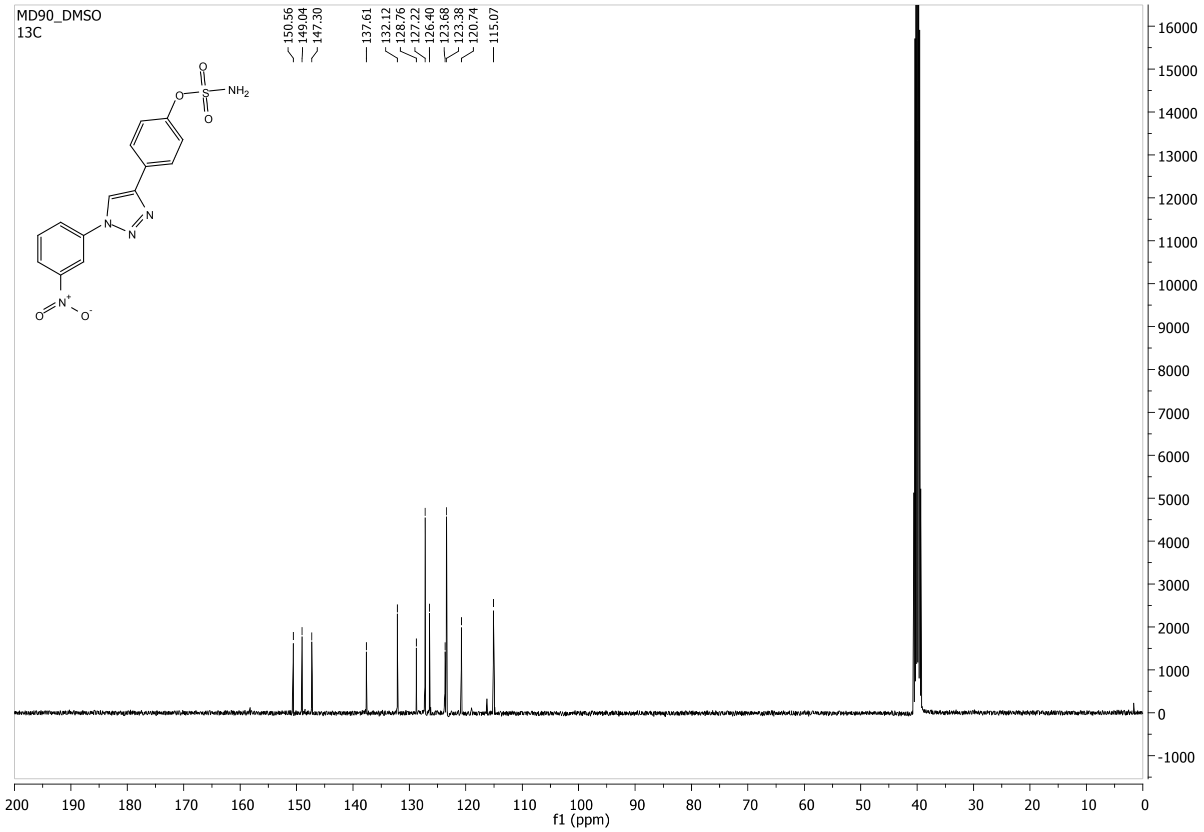
MD90_DMSO
13C
150.56
149.04
147.30
137.61
132.12
128.76
127.22
126.40
123.68
123.38
120.74
115.07
NH2
f1 (ppm)

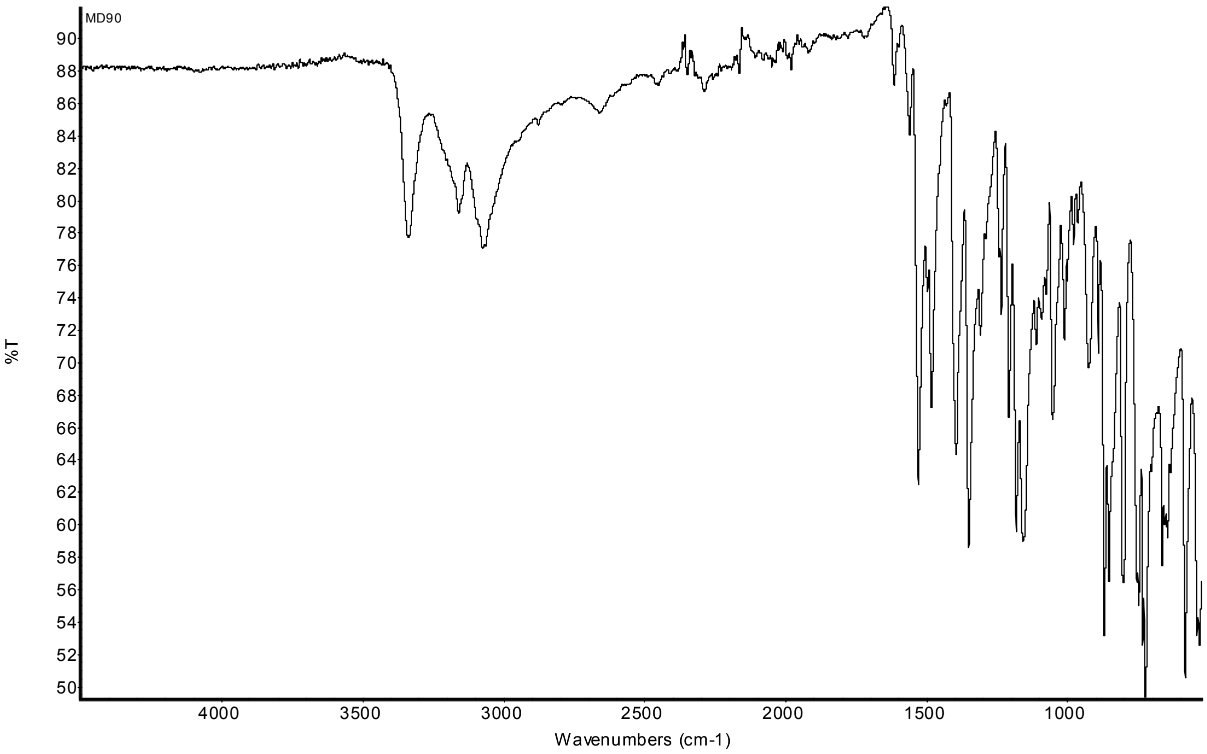

Acq. Time: 11:03
Acq. Date: Saturday, April 07, 2018

Batch Name: ManualTune.bat
Acq. File: MT20180407110356.wiff

Scan Mode: Zero Width
Polarity/Scan Type: Negative

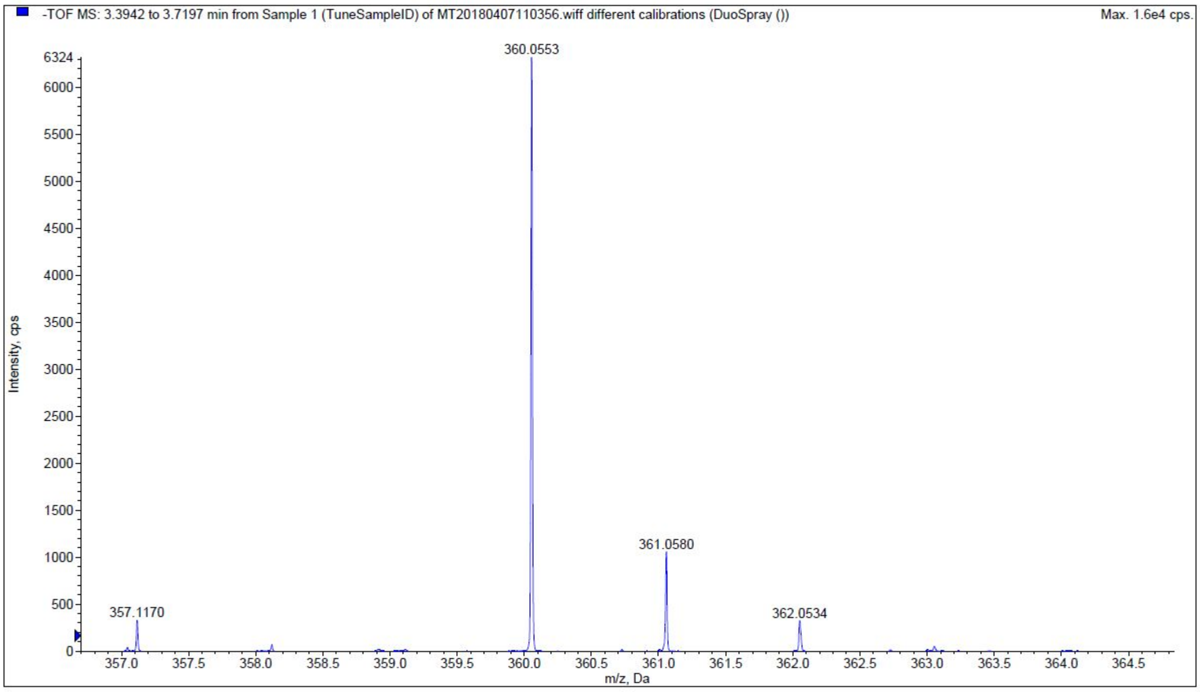

Printing Time: 11:09:00 AM
Printing Date: Saturday, April 07, 2018

Workstation: TRIPLETOF5600
Operator: Uniwersytet Gdanski

Analyst Version: 1.7.1
Page 1 of 1

HPLC purity 92.663 %

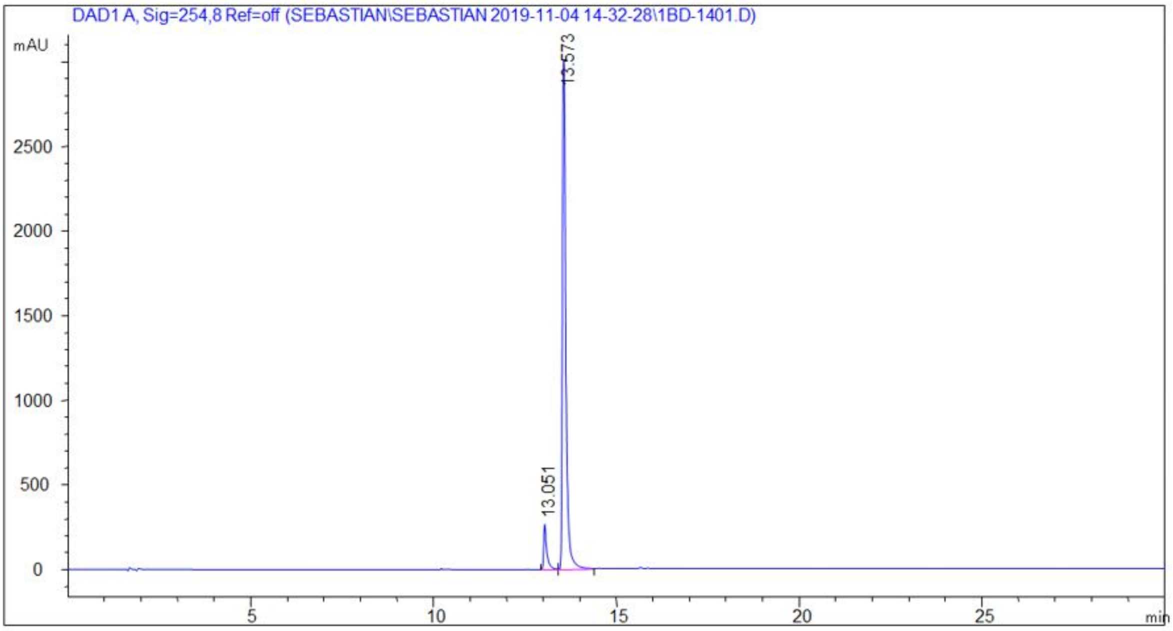

4l

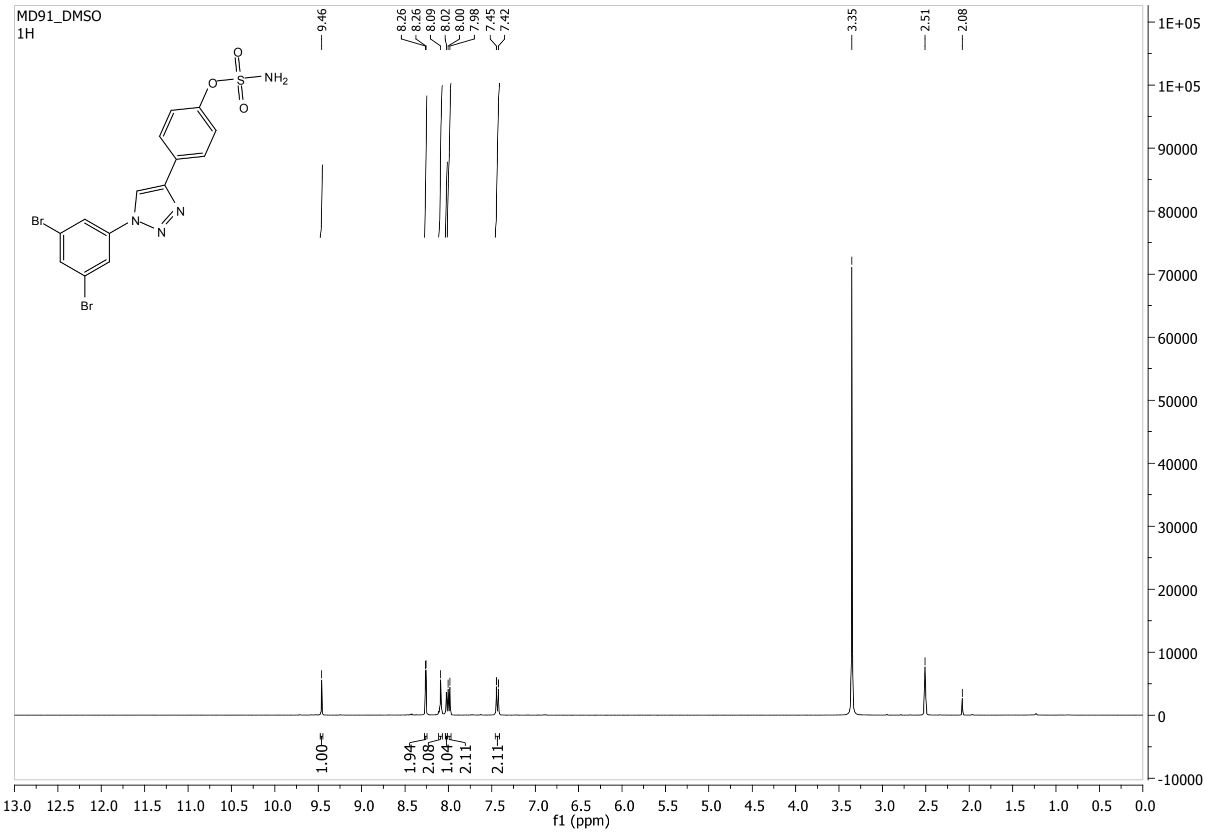
MD91_DMSO
1H
9.46
8.26
8.26
8.09
8.02
8.00
7.98
7.45
7.42
3.35
2.51
2.08
1.00
1.94
2.08
1.04
2.11
2.11
f1 (ppm)

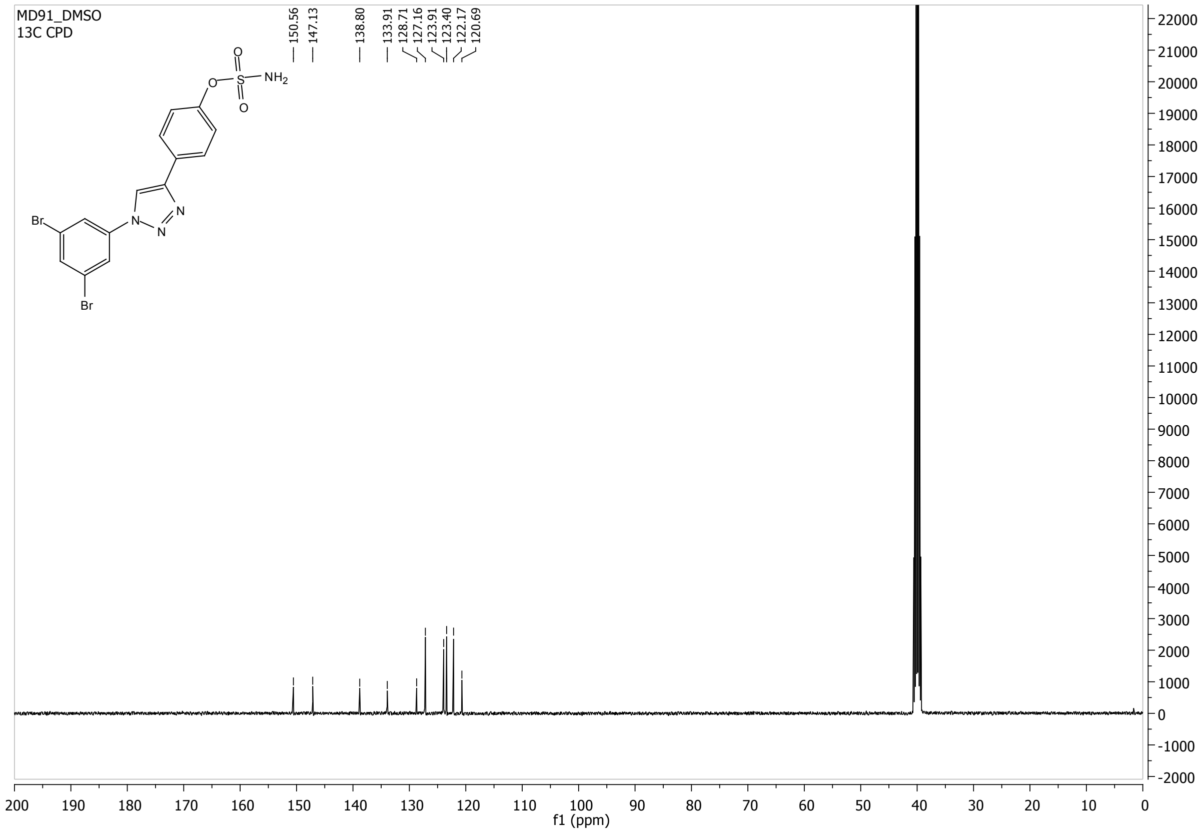
MD91_DMSO
13C CPD
150.56
147.13
138.80
133.91
128.71
127.16
123.91
123.40
122.17
120.69
f1 (ppm)

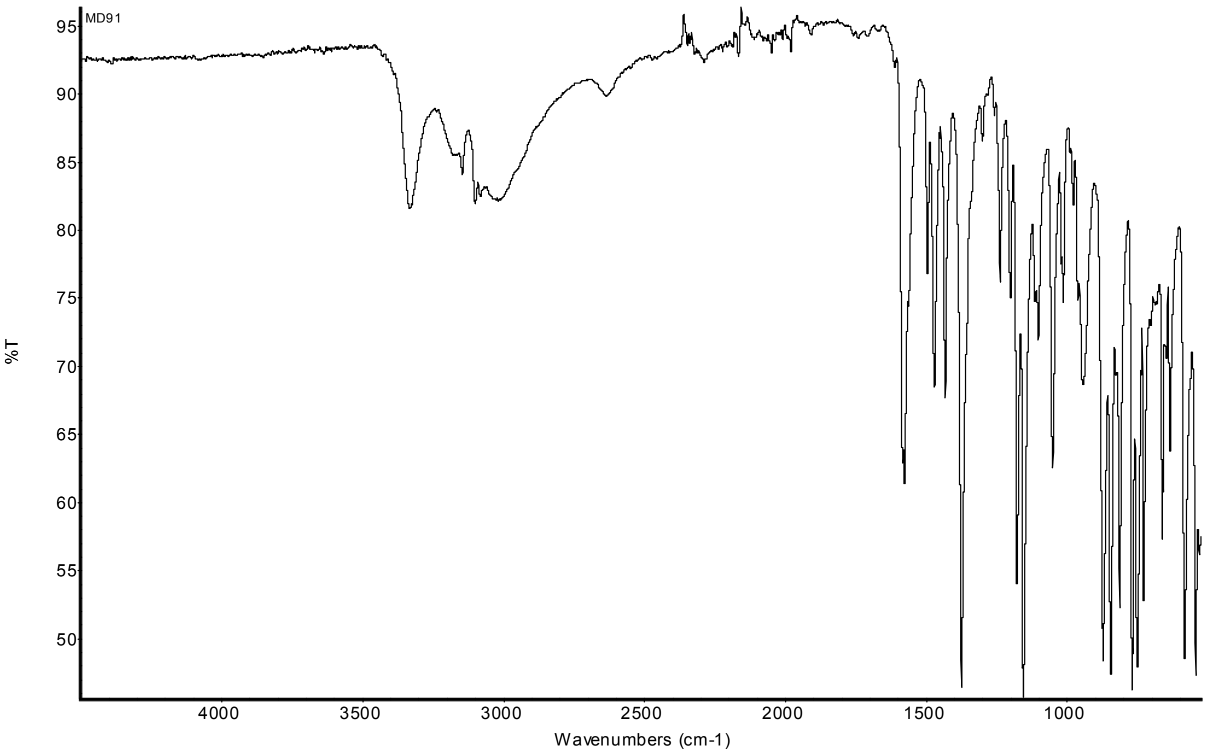

Acq. Time: 10:13
Acq. Date: Saturday, April 07, 2018

Batch Name: ManualTune.bat
Acq. File: MT20180407101353.wiff

Scan Mode: Zero Width
Polarity/Scan Type: Negative

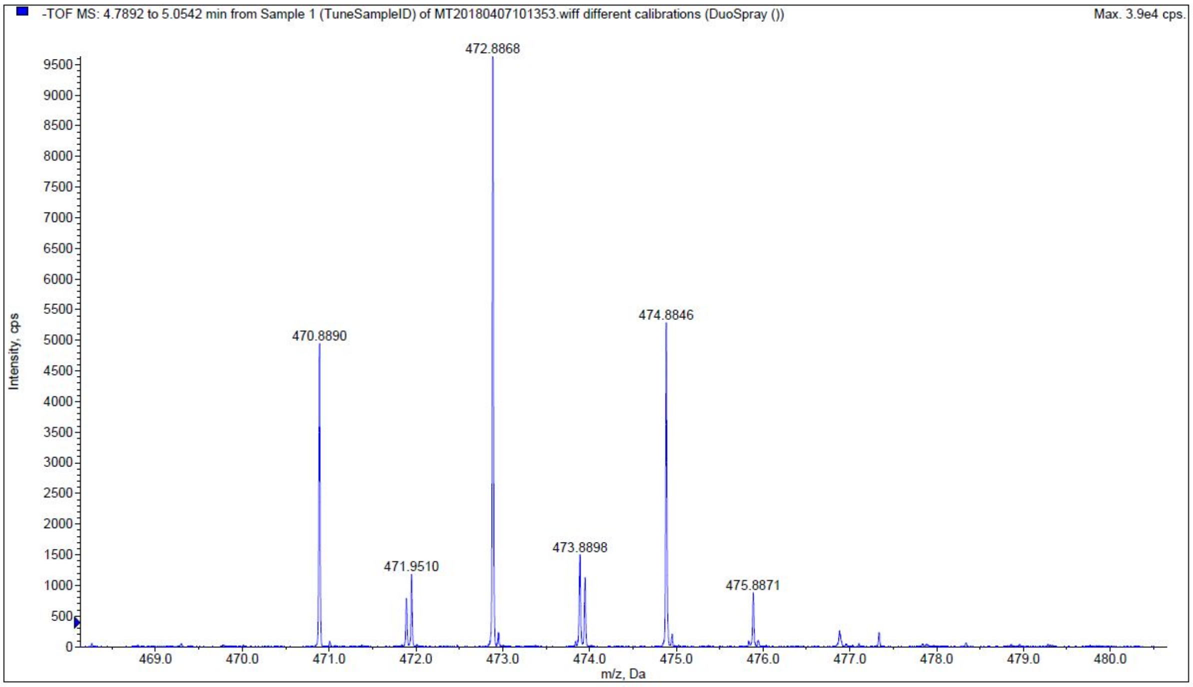

Printing Time: 10:20:13 AM
Printing Date: Saturday, April 07, 2018

Workstation: TRIPLETOF5600
Operator: Uniwersytet Gdanski

Analyst Version: 1.7.1
Page 1 of 1

HPLC purity 95.559 %

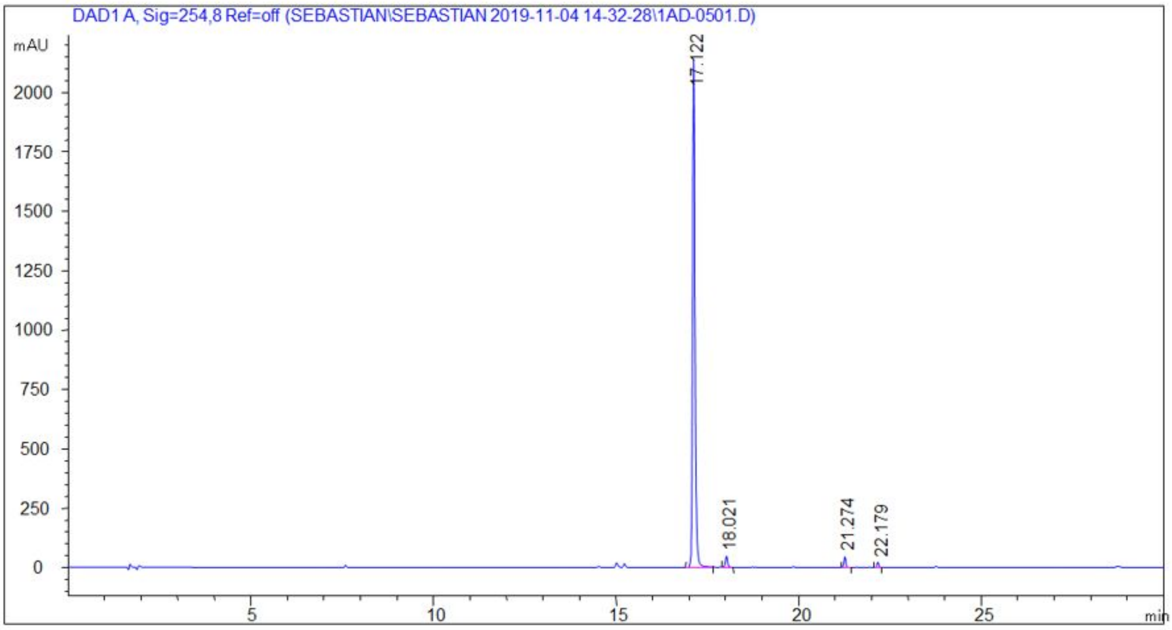

4m

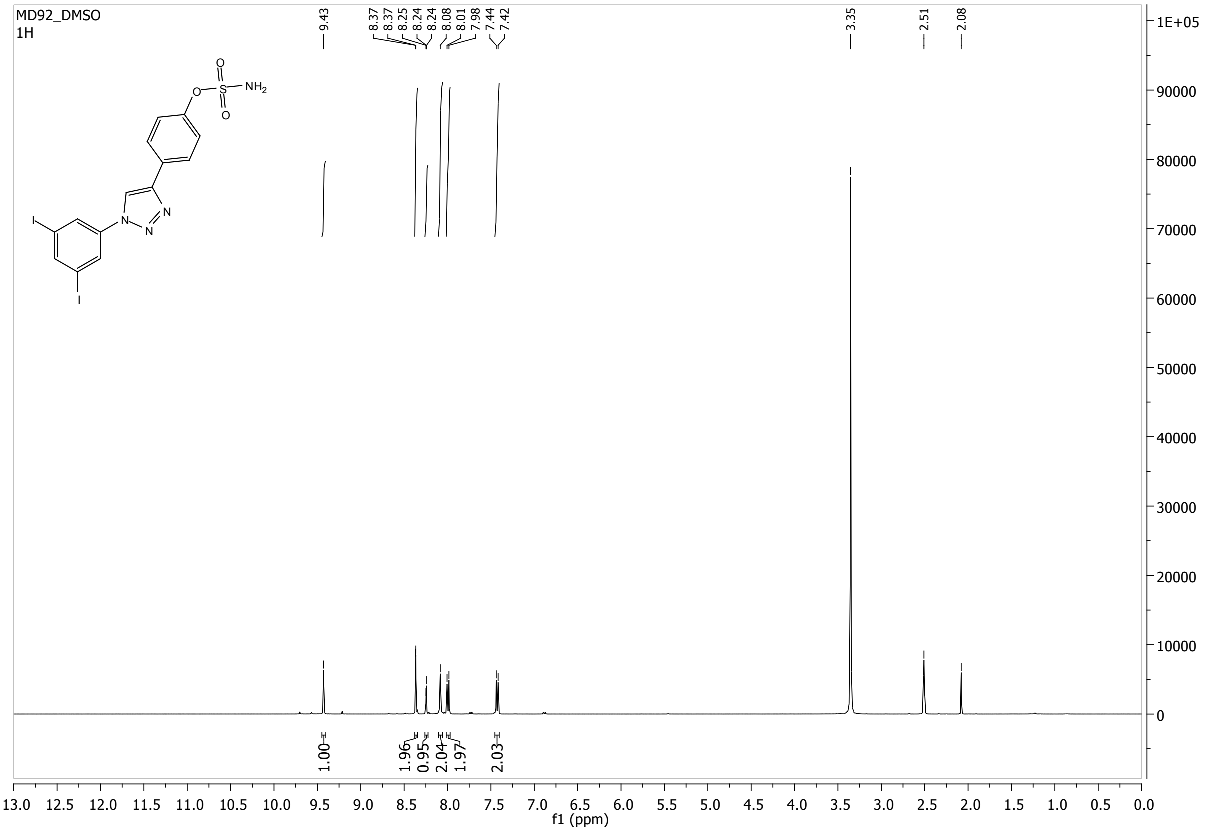

MD92_DMSO
1H
9.43
8.37
8.37
8.25
8.24
8.24
8.08
8.01
7.98
7.44
7.42
3.35
2.51
2.08
1.00
1.96
0.95
2.04
1.97
2.03
f1 (ppm)

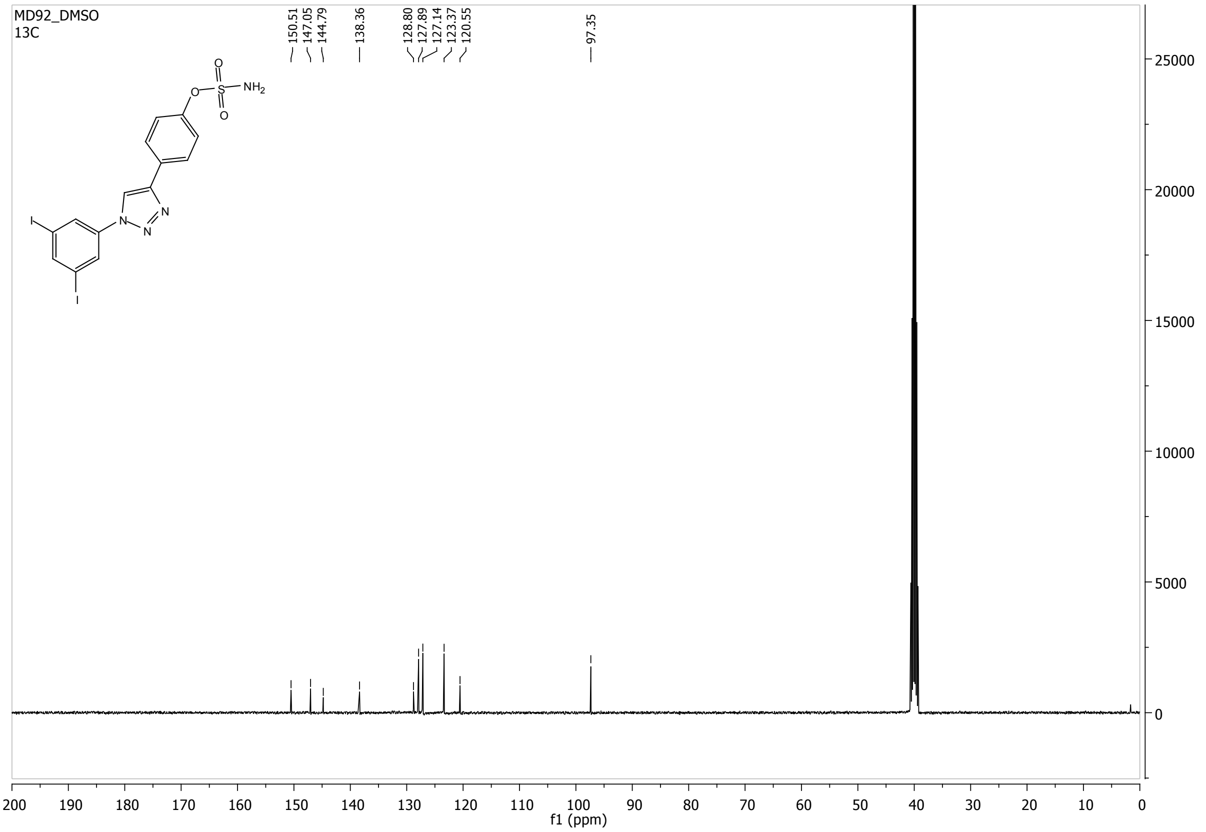

MD92_DMSO
13C
150.51
147.05
144.79
138.36
128.80
127.89
127.14
123.37
120.55
97.35
f1 (ppm)

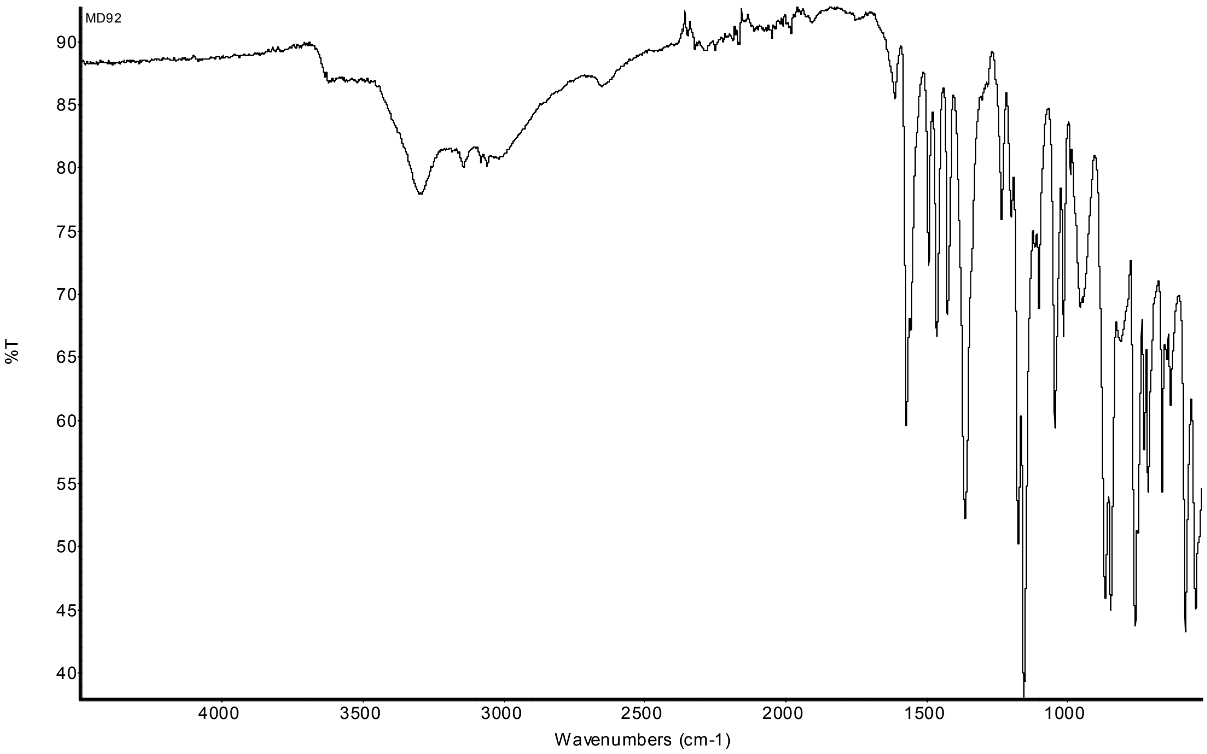

Acq. Time: 10:30
Acq. Date: Saturday, April 07, 2018
Batch Name: ManualTune.bat
Acq. File: MT20180407103028.wiff
Scan Mode: Zero Width
Polarity/Scan Type: Negative

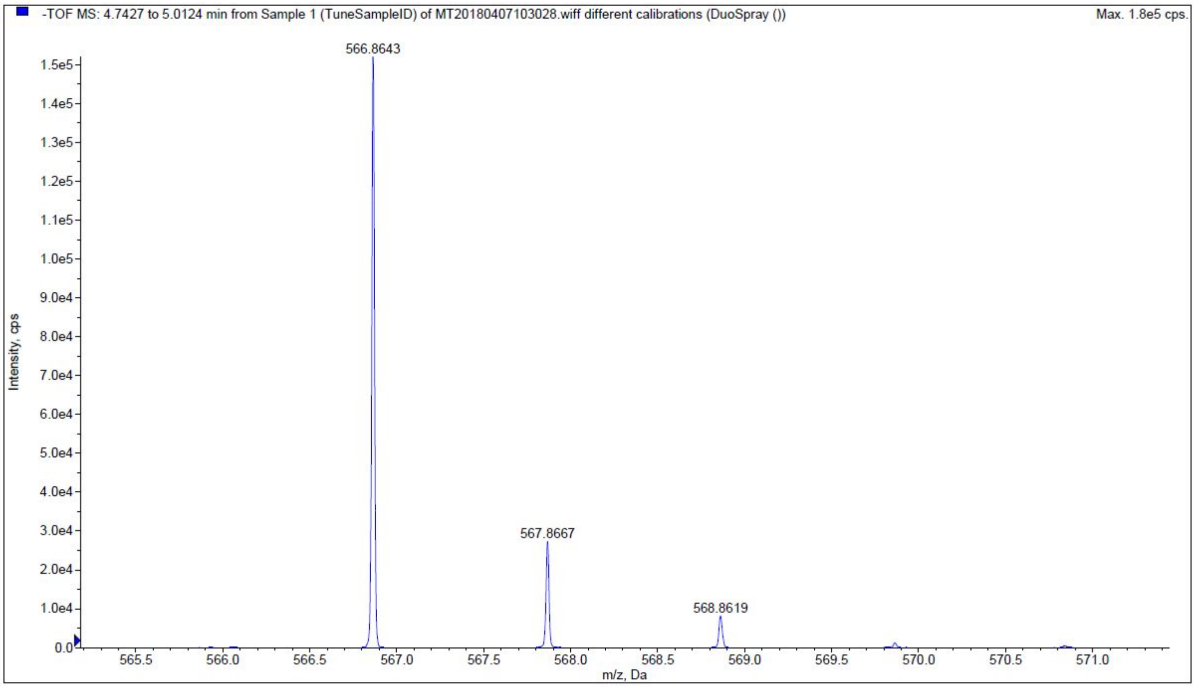

Printing Time: 10:36:39 AM
Printing Date: Saturday, April 07, 2018
Workstation: TRIPLETOF5600
Operator: Uniwersytet Gdanski
Analyst Version: 1.7.1
Page 1 of 1

HPLC purity 95.061 %

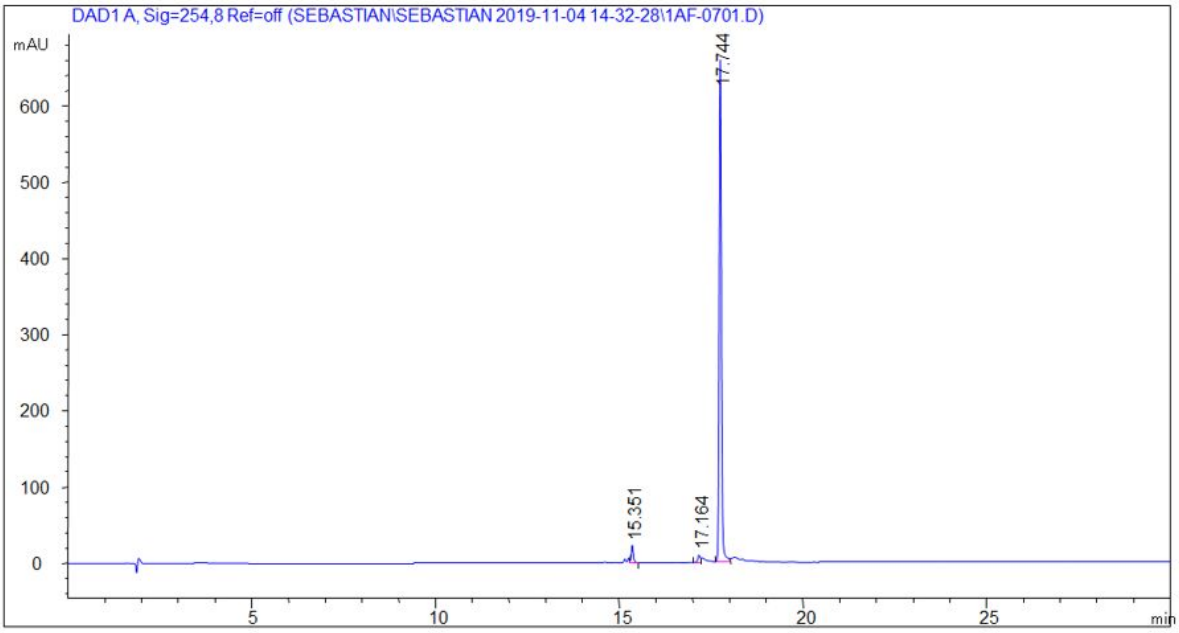

5e

HPLC purity 99.3 %

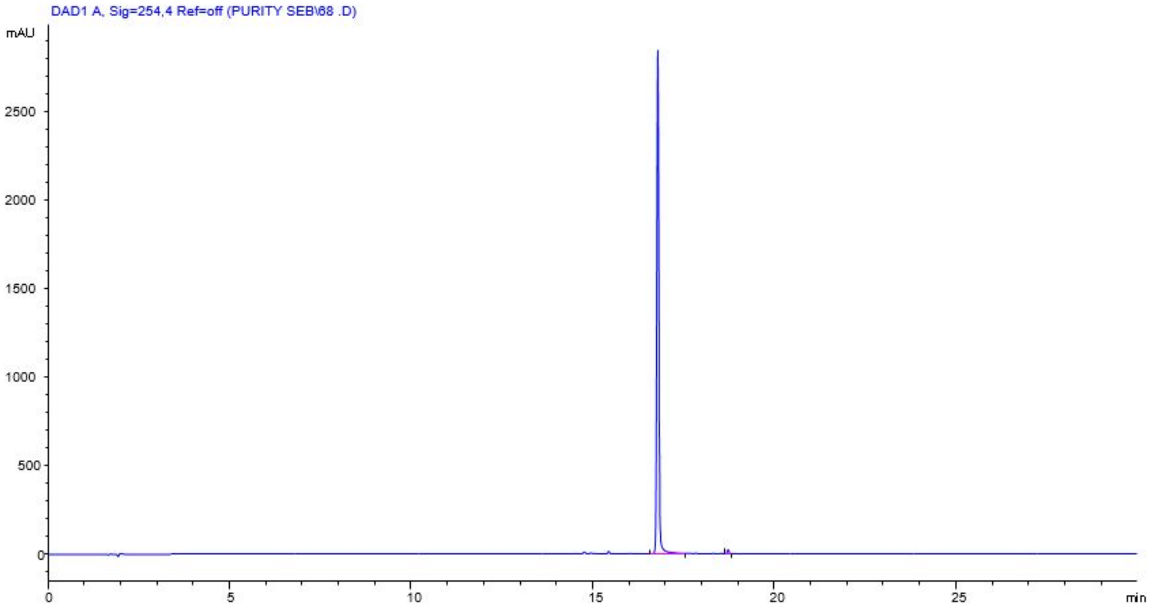

5g

HPLC purity 98.2 %

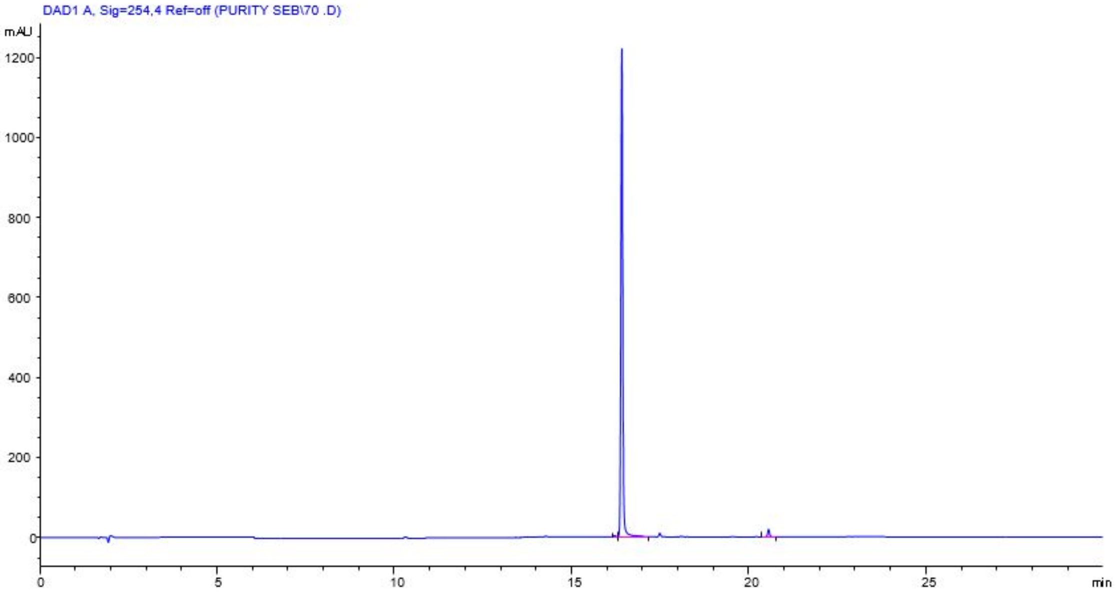

5l

HPLC purity 99.4 %

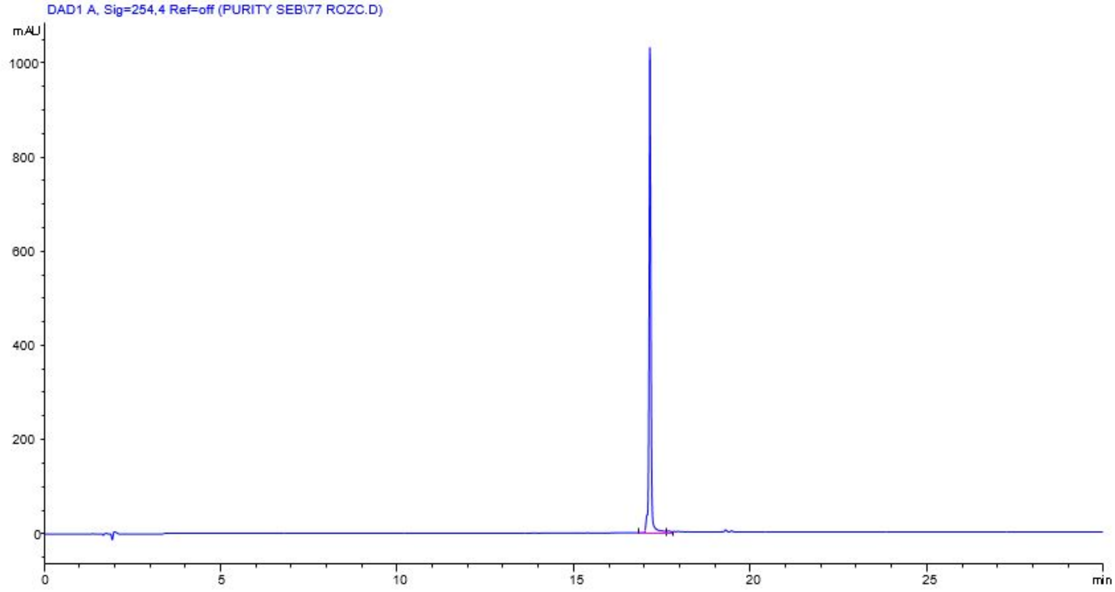

Supplement: Supplementary file 2 — jm1c02220_si_002.pdf [file jm1c02220_si_002.pdf]
